# 3′UTR-directed, kinase-proximal mRNA decay inhibits C/EBPβ phosphorylation/activation to suppress senescence in tumor cells

**DOI:** 10.1016/j.isci.2026.117039

**Published:** 2026-08-04

**Authors:** Jacqueline Salotti, Nida Asif, Srikanta Basu, Aniruddha Das, Linshan Hu, Mei Yang, Baktiar Karim, Karen Saylor, Nancy Martin, David A. Scheiblin, Sweta Misra, Brian T. Luke, Thorkell Andresson, Ming Yi, Mélissa Galloux, Stephen Lockett, Lino Tessarollo, Peter F. Johnson

**Affiliations:** 1 Mouse Cancer Genetics Program, Center for Cancer Research, National Cancer Institute, National Institutes of Health, Frederick, MD 21702, USA; 2 Molecular Histopathology Laboratory, Laboratory Animal Sciences Program, Frederick National Laboratory for Cancer Research, Frederick, MD 21702, USA; 3 Laboratory Animal Sciences Program, Frederick National Laboratory for Cancer Research, Frederick, MD, USA; 4 Optical Microscopy and Image Analysis Laboratory, Cancer Research Technology Program, Frederick National Laboratory for Cancer Research, Frederick, MD 21702, USA; 5 Advanced Biomedical Computational Science, Frederick National Laboratory for Cancer Research, Frederick, MD 21702, USA; 6 Protein Characterization Laboratory, Cancer Research Technology Program, Frederick National Laboratory for Cancer Research, Leidos Biomedical Research, Inc., Frederick, MD 21702, USA; 7 NCI RAS Initiative, Cancer Research Technology Program, Frederick National Laboratory for Cancer Research, Frederick, MD 21702, USA; 8 Independent Bioinformatician, Marseille, France

**Keywords:** 3′UTR, 3′UTR regulation of protein activity, oncogene-induced senescence, SASP cytokines, RAS signaling, cancer

## Abstract

C/EBPβ regulates oncogene-induced senescence (OIS) and the senescence-associated secretory phenotype (SASP) through activation by ERK1/2 and CK2. In tumor cells, C/EBPβ activity is suppressed by its 3′UTR via a mechanism termed 3′UTR regulation of protein activity (UPA), which spatially segregates *CEBPB* transcripts from kinase-rich perinuclear endosomes. Here, we identify kinase-proximal mRNA decay as the underlying mechanism. The mRNA decay factors UPF1 and STAU1/2 localize to perinuclear endosomes and promote degradation of *CEBPB* transcripts, thereby preventing C/EBPβ phosphorylation and activation. Disruption of this pathway restores C/EBPβ activity and induces senescence. *In vivo*, deletion of a G/U-rich regulatory element (GRE) in the 3′UTR impairs the progression of Kras-driven lung tumors and biases cells toward an AT2-like differentiation state with reduced EMT-associated transcriptional reprogramming. RAS-expressing *GRE*^*Δ/Δ*^ fibroblasts show enhanced OIS that requires upregulation of the pro-senescent cytokine S100a9. These findings identify perinuclear mRNA decay as a mechanism suppressing C/EBPβ activity and senescence in cancer.

## Highlights

• 3′UTR-directed perinuclear mRNA decay suppresses C/EBPβ activation in tumor cells
• UPF1 and STAU mediate decay of *CEBPB* transcripts at signaling endosomes
• Disrupting mRNA decay restores C/EBPβ-driven senescence
• Deletion of a key *Cebpb* 3′UTR element impairs malignant tumor progression *in vivo*

## Graphical abstract

**Figure:**
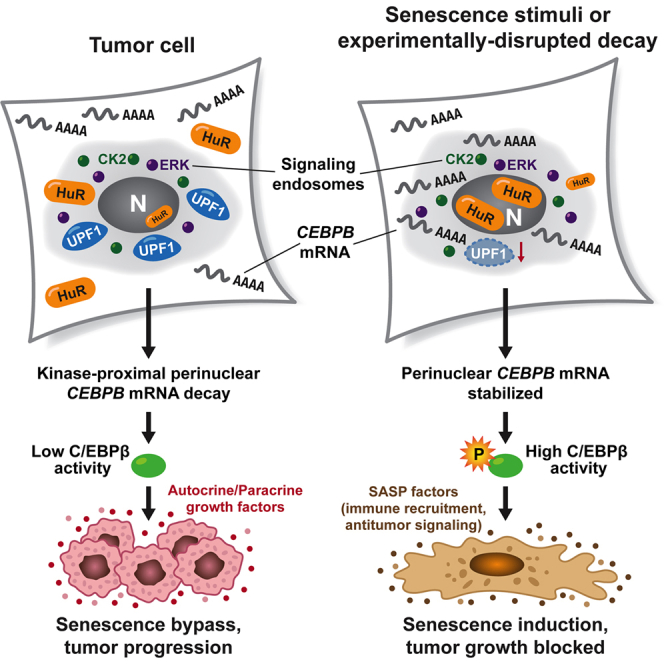

## Teaser

Molecular biology; Cell biology; Cancer

## Introduction

In addition to their well-established roles in mRNA stability, trafficking, and translation, 3′ untranslated regions (3′UTRs) can also regulate protein function. We previously showed that 3′UTR sequences suppress phosphorylation and activity of the pro-senescence regulator C/EBPβ in tumor cells.^1^ Subsequent studies demonstrated additional mechanisms by which 3′UTRs influence protein function. For example, the CD47 3′UTR directs trafficking of the encoded protein to the plasma membrane through recruitment of interacting factors.^2^ A cancer-associated 3′UTR splice variant of *CTNNB1* (β-catenin) enhances its oncogenic activity,^3^ and the 3′UTR of NET1 mRNA (encoding a guanine nucleotide exchange factor) localizes transcripts to cell protrusions, thereby regulating NET1 protein interactions and promoting cell migration.^4^

Senescence is a stable form of cell-cycle arrest induced by stress signals such as DNA damage, oxidative stress, and oncogene activation.^5^^,^^6^ Oncogene-induced senescence (OIS), triggered by activated *RAS* and other oncogenes, acts as a critical barrier to tumorigenesis.^7^^,^^8^ The OIS checkpoint imposes selective pressure for mutations that disable key pro-senescence pathways, including ARF-p53 and p16-RB, allowing premalignant cells to bypass senescence and progress toward malignancy. Senescent cells also express a characteristic set of proteins termed the senescence-associated secretory phenotype (SASP),^9^ which includes pro-inflammatory cytokines, their receptors, and matrix remodeling enzymes. SASP factors reinforce and propagate senescence through autocrine and paracrine signaling^10^^,^^11^^,^^12^ and promote immune-mediated clearance of senescent cells (senescence surveillance), an important tumor-suppressive mechanism *in vivo*.^13^

NF-κB and C/EBPβ are key transcriptional regulators of senescence and the SASP.^10^^,^^14^^,^^15^^,^^16^^,^^17^ Tumor cells, however, typically exhibit low expression of SASP genes, resulting in a relatively “cold” inflammatory phenotype that may contribute to immune evasion. This is paradoxical, as most cancer cells express C/EBPβ and activated NF-κB, which would be expected to induce senescence and SASP gene expression. We previously demonstrated that the pro-senescence function of C/EBPβ is inhibited in tumor cells through a 3′UTR-dependent mechanism that suppresses activating post-translational modifications.^1^^,^^18^ The 3′UTR also inhibits C/EBPβ-mediated activation of SASP genes. This mechanism, termed 3′UTR regulation of protein activity (UPA), involves spatial segregation of *CEBPB* transcripts away from a perinuclear cytoplasmic region enriched in the C/EBPβ kinases ERK1/2 and CK2. C/EBPβ translated outside this compartment is unable to access its activating kinases, resulting in reduced phosphorylation on Thr188 (ERK1/2) and Ser222 (CK2),^1^^,^^18^ which restrains the DNA-binding, homodimerization, and transactivation functions that are required for its senescence- and SASP-inducing activities.^18^^,^^19^^,^^20^^,^^21^

UPA is disabled in primary cells undergoing oncogenic RAS-induced senescence (RIS), enabling C/EBPβ activation in response to senescence signals. This occurs through an RAS-CaMKKβ-AMPK signaling pathway that promotes nuclear translocation of the RNA-binding protein HuR/ELAVL1.^22^ HuR binds a G/U-rich regulatory element (GRE) within the *Cebpb* 3′UTR^1^^,^^23^ and is required for UPA, as deletion of the GRE or depleting HuR disrupts transcript localization and relieves 3′UTR-mediated inhibition.^1^ Cytoplasmic HuR is frequently elevated in tumor cells and promotes malignancy by facilitating unrestrained proliferation and senescence bypass through multiple target mRNAs,^24^^,^^25^ including repression of C/EBPβ activity via UPA.

Although HuR and the GRE are essential for UPA, the mechanisms that control *CEBPB* mRNA localization and the full complement of factors required for this process are incompletely defined. Here, we identify additional components of the UPA machinery and uncover a role for localized mRNA decay in regulating C/EBPβ activity in tumor cells.

## Results

### Identification of *Cebpb* GRE-associated proteins

We hypothesized that a higher-order protein complex assembles on the GRE:HuR scaffold to establish UPA and inhibit C/EBPβ activity (Figure 1A). To identify additional *Cebpb* GRE-interacting factors, we performed an *in vitro* RNA affinity purification/mass spectrometry (MS) procedure previously used to characterize proteins binding A/U-rich elements (AREs)^26^ (Figure S1A). Proteins associating with a 102-nt *Cebpb* GRE RNA were purified from NIH 3T3 cytoplasmic lysates, as these cells are UPA-proficient and express the relevant factors. GRE-bound proteins were identified by comparison to those interacting with a control GFP RNA of similar length. The GRE-bound fraction displayed ∼50 visible bands on a Coomassie-stained gel (Figure 1B).

**Figure 1:**
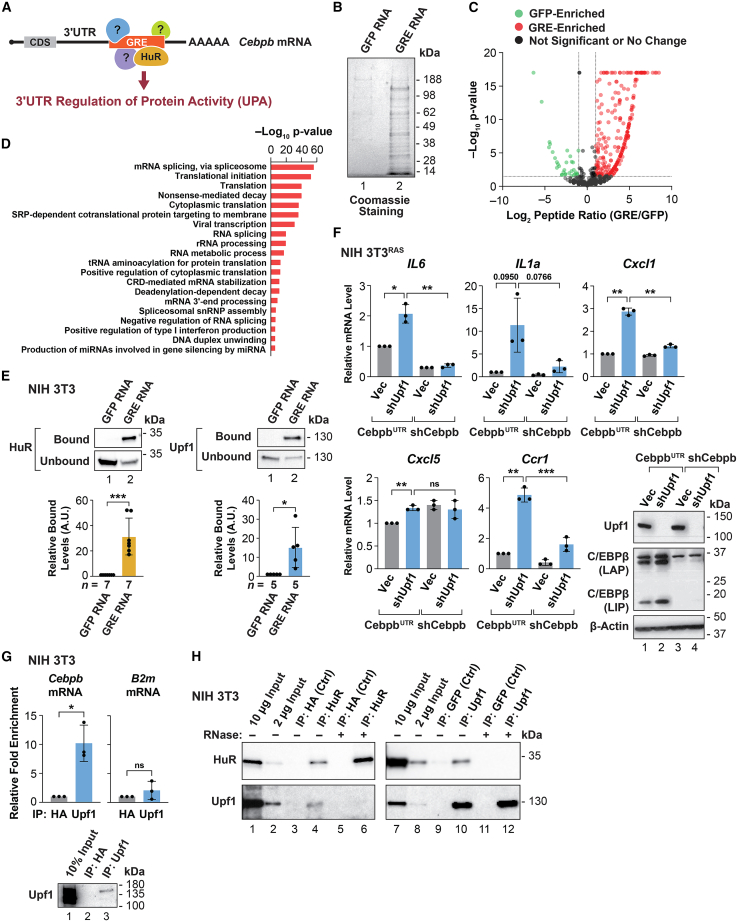
The *Cebpb* 3′UTR GRE motif associates with mRNA decay factor Upf1 (A) Depiction of HuR and other proteins binding to the *Cebpb* GRE to establish 3′UTR inhibition (UPA). (B) Coomassie-stained gel of proteins binding to the GRE RNA or a control GFP RNA. (C) Volcano plot of proteins displaying positive or negative enrichment for GRE binding, as determined by mass spectrometry. GRE/GFP peptide count ratios are the sum of three independent affinity purification experiments. Thresholds of log_2_ (GRE:GFP) ≥ 1 and −log_10_ (*p* value) ≥ 1.301 were used to identify 324 GRE-interacting proteins. 44 proteins had *p* values < 10^−17^ and were plotted as equal to 10^−17^ for graphing purposes only. (D) Gene Ontology analysis of proteins preferentially binding to the GRE. GRE-enriched proteins were analyzed for associations with pathways/biological processes using DAVID.^27^ GO terms for the highest-ranking biological processes are shown, ranked by *p* value. (E) Immunoblots confirming binding of HuR and Upf1 to the GRE RNA. Quantitation of binding enrichment for 5 replicate experiments is shown below. (F) Upf1 was depleted in 3T3^RAS^ cells overexpressing *Cebpb*^*UTR*^ (βUTR) or depleted for C/EBPβ (shβ) and expression of selected proinflammatory SASP mRNAs was analyzed by RT-qPCR. (G) Association of Upf1 with *Cebpb* or *B2m* (control) transcripts in NIH 3T3 cells. nRIP assays from 3 independent assays are shown. Bottom: immunoblots confirming IP of Upf1. (H) HuR and Upf1 associate in an RNA-dependent manner. IPs were performed using NIH 3T3 cell lysates in the absence or presence of 200 μg/mL RNase A. Data are presented as mean ± SD. Error bars indicate SD. Statistical significance was determined using Student’s *t* test. ∗*p* < 0.05, ∗∗*p* < 0.01, ∗∗∗*p* < 0.001.

Proteins from three independent pull-down experiments were analyzed by MS, and GRE binders were distinguished from non-specific proteins based on GRE:GFP peptide ratios. Using thresholds of log_2_ (GRE:GFP) ≥ 1 and −log_10_ (*p* value) ≥ 1.301, 324 GRE-interacting proteins were identified (Figure 1C and Table S1). Gene Ontology (GO) analysis (DAVID, GO TERM_BP; Gene Ontology Biological Process)^27^ revealed enrichment for RNA-associated biological processes, including translation, splicing, and RNA decay (Figure 1D and Table S1).

To identify functional UPA components, we performed a secondary RNAi screen targeting 21 candidate GRE interactors selected based on enrichment scores and prior pilot data (Table S1). As a functional readout, we measured expression of C/EBPβ-regulated SASP genes, reasoning that depletion of bona fide UPA factors would activate C/EBPβ and induce the SASP (Figure S1B). Experiments were conducted in HRAS^G12V^-transformed NIH 3T3 cells expressing a *Cebpb*^UTR^ construct (3T3^RAS^-β^UTR^), as these cells express low endogenous C/EBPβ.^28^ To confirm C/EBPβ dependence, knockdowns were also performed in NIH 3T3^RAS^ cells with additional depletion of endogenous C/EBPβ (3T3^RAS^-sh*Cebpb*).

Knockdown of Strbp and Upf1 (RENT1) increased *Il6*, *Il1α*, *Cxcl1*, and *Ccr1* expression in 3T3^RAS^-β^UTR^ cells but not in 3T3^RAS^-sh*Cebpb* cells (Figure S1B), indicating a C/EBPβ-dependent effect. Silencing of other candidates, including Elavl1 (HuR) and Fubp3, induced *Ccr1* expression but did not broadly activate SASP genes. These results implicate Upf1 and Strbp as candidate components of the UPA machinery.

Upf1 is an RNA helicase/ATPase and RNP remodeling factor that regulates nonsense-mediated mRNA decay (NMD) and related RNA degradation pathways.^29^^,^^30^^,^^31^ We prioritized Upf1 for further study based on its strong induction of SASP genes upon depletion (Figure S1B) and prior evidence of UPF1 binding to *CEBPB* mRNA in genome-wide CLIP-seq datasets^32^^,^^33^ (Table S2). Notably, several RNA-independent and RNA-dependent Upf1-interacting proteins^34^ overlapped with the GRE-binding dataset (Figure S1C and Table S3). In addition, UPF1 ablation exhibits synthetic lethality with oncogenic KRAS,^35^ consistent with our model that disruption of C/EBPβ UPA impairs proliferation or survival of RAS-transformed cells. Immunoblotting confirmed selective enrichment of Upf1 and HuR in the GRE-bound fraction relative to control RNA (Figure 1E).

We next confirmed that Upf1 depletion activates SASP genes in a C/EBPβ-dependent manner in 3T3^RAS^ cells (Figure 1F). Native RNA immunoprecipitation (nRIP) demonstrated that Upf1 associates with *Cebpb* transcripts but not a control mRNA (β_2_-microglobulin) (Figure 1G). Co-immunoprecipitation assays further showed that Upf1 and HuR interact in NIH 3T3 cells and that this interaction is disrupted by RNase treatment, indicating RNA-dependent association (Figure 1H). These findings are consistent with proteomic data identifying HuR as an RNA-dependent UPF1 interactor.^34^

### Perinuclear Upf1-dependent mRNA decay controls subcellular partitioning of *CEBPB* transcripts

To determine whether Upf1 contributes to UPA-mediated inhibition of C/EBPβ activity, we first examined the subcellular distribution of *Cebpb* transcripts and Upf1 in NIH 3T3 cells. RNA fluorescence in situ hybridization (FISH) showed that *Cebpb* mRNAs were excluded from the perinuclear region (Figure S2A), as previously reported.^22^ Consistent with this pattern, Upf1 was localized to the perinuclear area, which was largely devoid of *Cebpb* transcripts. Upon Upf1 depletion, *Cebpb* transcripts accumulated in this region, particularly in cells with efficient Upf1 knockdown.

Because NIH 3T3 cells are not transformed and do not exhibit constitutive perinuclear localization of CK2 and *p*-ERK,^18^ and because the elongated morphology of RAS-transformed NIH 3T3 cells complicates spatial analysis, we examined human A549 lung adenocarcinoma (ADC) cells (*KRAS*^*G12S*^). Immunofluorescence (IF) imaging revealed that UPF1 and CK2α were enriched in the perinuclear region and largely non-overlapping with *CEBPB* transcripts (Figure 2A). Depletion of UPF1 increased the abundance of *CEBPB* transcripts near the nucleus, as quantified by relative nuclear proximity indices (RNPIs)^36^ (Figure 2A). To assess overlap with CK2, we defined a perinuclear kinase region by thresholding CK2α fluorescence and measured the fraction of *CEBPB* FISH signals within this boundary (Figure 2B). In control cells, 25.6% of *CEBPB* transcripts overlapped with the CK2 region, increasing to 62.6% following UPF1 depletion (*p* < 0.0001).

**Figure 2:**
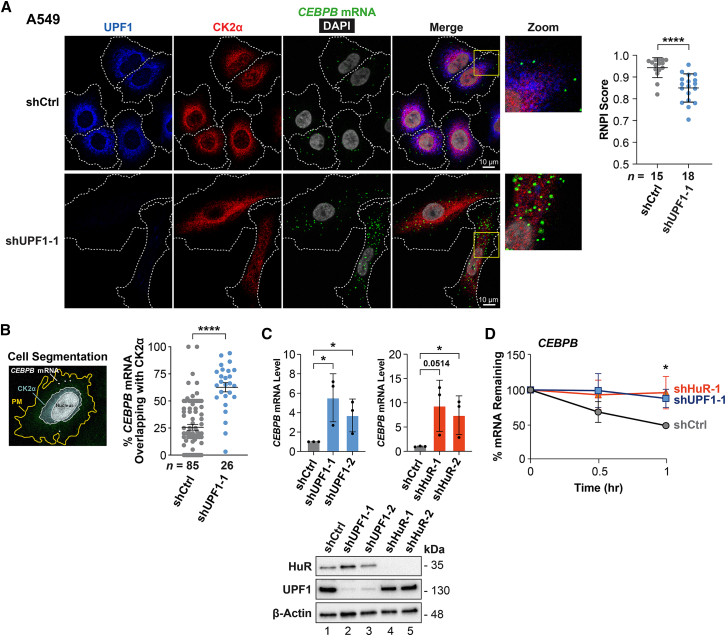
UPF1 controls perinuclear decay of *CEBPB* transcripts (A) UPF1 depletion in A549 lung adenocarcinoma cells increases the abundance of *CEBPB* transcripts in the perinuclear region containing CK2. *CEBPB* mRNA was detected using single-molecule RNA FISH in combination with UPF1 and CK2α IF staining. Right: nuclear proximity of *CEBPB* transcripts (RNPI score/cell). (B) Overlap of perinuclear CK2α and *CEBPB* mRNA increases after UPF1 silencing in A549 cells. A perinuclear CK2α domain was defined by fluorescence thresholding and the number of cytoplasmic *CEBPB* mRNA signals within this domain was determined for each cell. *n*, number of cells analyzed. (C) UPF1 or HuR depletion increases total *CEBPB* mRNA levels in A549 cells. mRNA levels were determined by RT-qPCR analysis of RNA samples, normalized to *PPIA* mRNA. Below: immunoblot confirming shRNA depletions. (D) *CEBPB* mRNA is stabilized by depletion of UPF1 or HuR. Decay rates were measured by EU pulse-chase labeling, followed by RT-qPCR analysis of EU-containing *CEBPB* transcripts, normalized to *PPIA* mRNA. Scale bar lengths are shown in the micrographs. *n*, number of cells analyzed. Data are presented as mean ± SD. Error bars indicate SD. Statistical significance was determined using Student’s *t* test. ∗*p* < 0.05, ∗∗∗∗*p* < 0.0001.

Given the established role of UPF1 in mRNA decay, its enrichment in perinuclear areas lacking *CEBPB* transcripts suggested that localized degradation prevents mRNA accumulation in this compartment. Consistent with this model, depletion of UPF1 or HuR in A549 cells increased total *CEBPB* mRNA levels, as measured by RT-qPCR (Figure 2C). Similar results were observed in RAS-transformed NIH 3T3 cells (Figure S2B). UPF1 depletion also increased *CEBPB* mRNA half-life (Figure 2D). Stabilization of *CEBPB* mRNA was likewise observed upon HuR depletion, which also promotes the perinuclear localization of *CEBPB* mRNAs.^1^ Together, these findings indicate that UPF1 and HuR mediate perinuclear mRNA decay (PMD).

During NMD, UPF1 activity is stimulated by SMG1-dependent phosphorylation of Ser1127, which stimulates recruitment of SMG6, an endonuclease that cleaves the target transcript, and other components such as SMG5/7 that initiate de-capping, de-adenylation, and exonucleolytic mRNA degradation.^29^ To assess the contribution of these factors to PMD, we depleted SMG1, SMG5, SMG6, and SMG7 in A549 cells. Loss of SMG1 or SMG5 did not affect exclusion of *CEBPB* transcripts from the CK2-rich perinuclear region, as assessed by RNPI (Figure S2D), although SMG1 depletion reduced UPF1 Ser1127 phosphorylation as expected (Figure S2E). In contrast, SMG6 or SMG7 knockdown markedly increased both nuclear proximity and overall abundance of *CEBPB* transcripts (Figure S2D). In control cells, most *CEBPB* transcripts were localized away from perinuclear SMG6/7-enriched regions. These results indicate that SMG6 and SMG7, but not SMG1 or SMG5, are key components of the PMD pathway.

### UPF1 suppresses C/EBPβ phosphorylation and senescence in tumor cells

Segregation of *CEBPB* transcripts from perinuclear CK2 in tumor cells inhibits phosphorylation of C/EBPβ on a target CK2 site (Ser222 in mouse, Ser271 in human), which promotes C/EBPβ DNA binding and pro-senescence activity.^18^ Super-resolution imaging of A549 cells depleted of UPF1 revealed increased localization of *CEBPB* transcripts adjacent to CK2α foci (Figure 3A), which correspond to perinuclear signaling endosomes.^18^^,^^36^ Nearest-neighbor analysis (16 cells/group) confirmed a significant increase in *CEBPB* mRNA proximity to CK2α upon UPF1 depletion (Figure 3A, right). Proximity ligation assays (PLAs) using C/EBPβ and CK2α antibodies further showed increased juxtaposition of these proteins in the cytoplasm of UPF1-depleted cells (Figure 3B). These results indicate that loss of UPF1 increases the spatial coupling of *CEBPB* transcripts and nascent C/EBPβ with CK2-containing endosomes, thereby facilitating phosphorylation prior to nuclear translocation.

**Figure 3:**
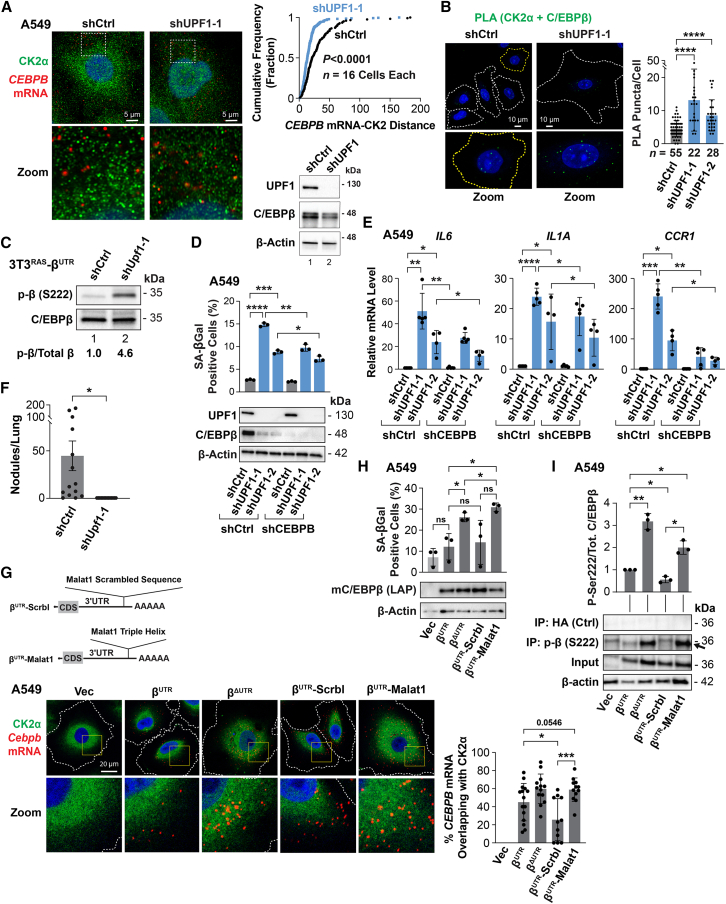
UPF1 depletion in tumor cells stimulates C/EBPβ activity and phosphorylation by CK2α (A) High-resolution imaging showing close association of *CEBPB* mRNAs and CK2α foci in the perinuclear cytoplasm of UPF1-depleted cells. Right: nearest neighbor analysis of *CEBPB* mRNA and the closest CK2α signal (cumulative frequency plot of distance in pixels). Statistical significance was determined using the Kolmogorov-Smirnov test. (B) Proximity ligation assay (PLA) demonstrating increased contiguity of C/EBPβ and CK2α proteins following UPF1 depletion. Right: PLA quantification (PLA puncta per cell). (C) Upf1 knockdown increases phosphorylation on C/EBPβ Ser222 (CK2 site). Chromatin-bound proteins from 3T3^RAS^-β^UTR^ cells were released by DNase digestion, equalized for total C/EBPβ and analyzed by immunoblotting using a p-Ser222 phospho-specific antibody. (D) UPF1 knockdown induces senescence in tumor cells. UPF1 was ablated in A549 cells with or without *CEBPB* knockdown (shCEBPB) and SA-βGal staining was used to analyze senescence (*n* ≥ 400 cells). (E) UPF1 depletion induces expression of SASP genes that is partially C/EBPβ-dependent. SASP mRNA levels were analyzed using RT-qPCR. (F) 1 × 10^6^ control or UPF1-depleted A549 cells were injected intravenously into nude mice and lung nodules were counted after 7 weeks. *n* = 14 animals per group. (G) Insertion of the *Malat1* stability element at the 3′ end of *Cebpb* mRNA disrupts peripheral localization of *Cebpb* transcripts. Mouse *Cebpb* constructs containing the *Malat1* triple helix or a scrambled version inserted at the 3′ end (diagram) were transduced into A549 cells. *Cebpb*^*UTR*^ (βUTR) and *Cebpb*^*ΔUTR*^ (*Δ*UTR) were included as controls. RNA FISH was performed using a probe specific for mouse *Cebpb*, combined with IF staining for CK2α. Right: analysis of *Cebpb* mRNA overlap with the CK2 region. (H) SA-βGal staining was used to analyze senescence in the cells described in (G) (*n* ≥ 400 cells). (I) Analysis of C/EBPβ Ser222 phosphorylation using the cells described in (G). Nuclear lysates were immunoprecipitated with p-C/EBPβ (S222) antibody followed by immunoblotting for total C/EBPβ. HA antibody was used as an IP isotype control for IPs. Data are from three independent replicates. Scale bar lengths are shown in the micrographs. *n* = number of cells analyzed. Data are presented as mean ± SD. Error bars indicate SD. Statistical significance, except for the analysis in (A), was determined using Student’s *t* test. ∗*p* < 0.05, ∗∗*p* < 0.01, ∗∗∗*p* < 0.001, ∗∗∗∗*p* < 0.0001.

Because antibodies that efficiently detect human C/EBPβ p-Ser271 were unavailable, we used an antibody specific for mouse p-Ser222^18^ to monitor CK2-mediated phosphorylation in the chromatin fraction of 3T3^RAS^-β^UTR^ cells. UPF1 depletion increased p-Ser222 levels (normalized to total C/EBPβ) by 4.6-fold (Figure 3C) and enhanced C/EBPβ DNA binding and homodimerization, with the proportion of electrophoretic mobility shift assay (EMSA) homodimer species rising 8-fold relative to controls (Figure S3A). Increased C/EBPβ DNA binding was also observed upon UPF1 knockdown in PANC-1 pancreatic ductal ADC cells and HRAS-transformed, *p19*^*Arf*^-deficient mouse embryo fibroblasts (MEFs) (Figure S3B). These findings indicate that UPF1 suppresses C/EBPβ phosphorylation and DNA-binding activity in transformed cells.

UPF1 depletion robustly induced senescence, as measured by senescence-associated β-galactosidase (SA-βGal) staining, in a partially C/EBPβ-dependent manner in A549 cells (Figure 3D) and additional human tumor cell lines (Figure S3C). Consistent with this phenotype, UPF1 ablation strongly upregulated pro-inflammatory SASP genes in a C/EBPβ-dependent manner (Figures 3E and S3D), with similar effects in PANC-1 and SW-900 cells and RAS-transformed MEFs (Figure S3E). UPF1-deficient A549 cells also failed to form lung lesions following intravenous injection into immunodeficient mice (Figure 3F), demonstrating impaired tumorigenic capacity. UPF1 was markedly downregulated in HRAS^G12V^-induced senescent human IMR90 fibroblasts and *WT* MEFs but remained expressed in RAS-transformed *p19*^*Arf−/−*^ cells (Figure S3E), consistent with its role in suppressing OIS.

To directly test whether mRNA decay is critical for subcellular partitioning of *CEBPB* transcripts, we generated mouse *Cebpb* constructs containing either the triple helical *Malat1* RNA stability element^37^ or a scrambled version (Scrbl) at the 3′ end of the 3′UTR (Figure 3G). These constructs, along with *Cebpb*^*UTR*^ and *Cebpb*^*ΔUTR*^ controls, were expressed in A549 cells. RNA FISH using a probe specific for murine *Cebpb*, combined with CK2α IF, showed that *Cebpb*^*UTR*^ and *Cebpb*^*Scrbl*^ transcripts were excluded from the perinuclear region (Figure 3G). In contrast, *Cebpb*^*ΔUTR*^ and *Cebpb*^*Malat1*^ transcripts accumulated near CK2 and showed increased overlap with the kinase compartment (Figure 3G, right). These constructs also increased the proportion of senescent cells, whereas *Cebpb*^*UTR*^ and *Cebpb*^*Scrbl*^ maintained basal levels (Figure 3H). The senescence phenotype correlated with increased phosphorylation on Ser222 (Figure 3I). These results demonstrate that stabilization of *Cebpb* mRNA overrides 3′UTR-mediated exclusion, supporting a model in which localized mRNA decay restricts C/EBPβ synthesis in kinase-rich regions to suppress its activation and pro-senescence function.

### Staufen RNA-binding proteins are essential for *CEBPB* PMD

The double-stranded RBPs, Staufen 1 and 2, were also enriched in the GRE-associated fraction (Table S1; Figure S4A). STAU proteins regulate mRNA localization and decay,^38^ and STAU-mediated mRNA decay (SMD) requires UPF1.^39^ STAU2 depletion is also synthetically lethal with mutant *KRAS* in colorectal cancer cells.^35^ These observations prompted us to investigate a potential role for STAU proteins in *CEBPB* PMD.

nRIP binding assays showed that STAU1 and STAU2 associate with *CEBPB* transcripts in A549 cells (Figure 4A). Combined IF/RNA FISH analysis revealed that STAU1/2 are concentrated in perinuclear regions that partially overlap with UPF1 and are largely devoid of *CEBPB* transcripts (Figure 4B). Thus, STAU1/2 are likely involved in perinuclear *CEBPB* mRNA decay.

**Figure 4:**
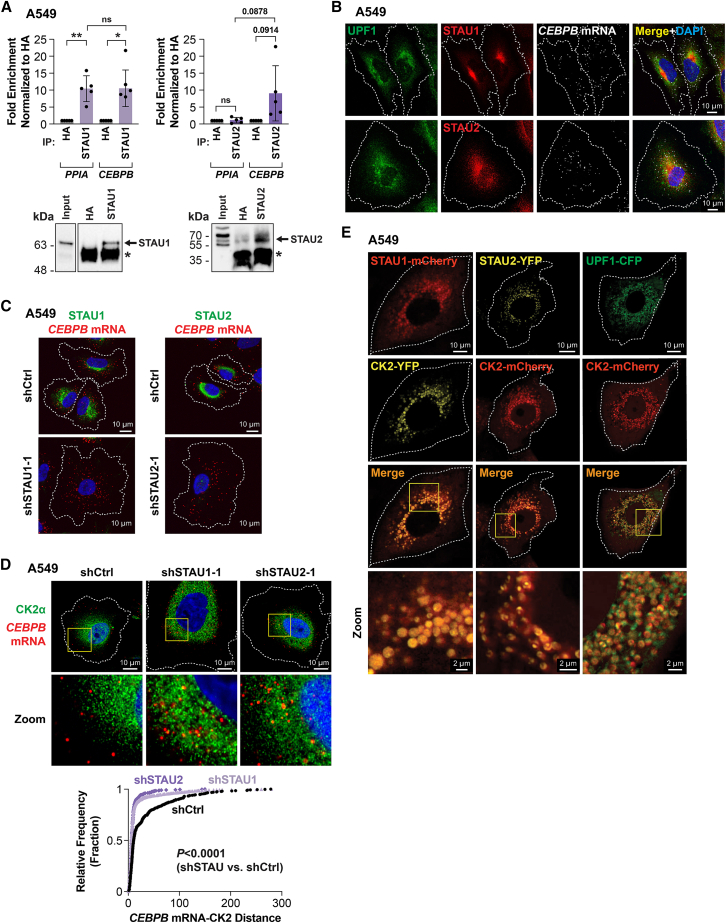
Staufen controls perinuclear exclusion of *CEBPB* mRNA in tumor cells (A) STAU1/2 associate with *Cebpb* transcripts in A549 cells. nRIP assays were performed using STAU1 and STAU2 IP antibodies followed by RT-qPCR to assess *CEBPB* and *PPIA* mRNA enrichment. Bottom: immunoblots for IP samples. ∗ denotes IgG light chain band. (B) STAU1 and STAU2 partially overlap with UPF1 in the perinuclear region lacking *CEBPB* mRNAs. IF staining in A549 cells was combined with *CEBPB* RNA FISH. (C) Depletion of STAU proteins disrupts perinuclear exclusion of *CEBPB* mRNAs. Cells were also immunostained with the appropriate antibodies to verify STAU knockdown. (D) High-resolution imaging showing close apposition of *CEBPB* mRNAs and perinuclear CK2α foci in A549 cells upon STAU1 or STAU2 depletion. Bottom: nearest neighbor analysis of CK2α and *CEBPB* mRNAs was performed as described in Figure 3A. (E) Live-cell imaging of fluorescently tagged proteins, demonstrating co-localization of STAU1, STAU2, and UPF1 with perinuclear CK2α signaling endosomes. Scale bar lengths are shown in the micrographs. *n*, number of cells or samples analyzed. Data are presented as mean ± SD. Error bars indicate SD. Statistical significance, except for the analysis in (D), was determined using Student’s *t* test. ∗*p* < 0.05, ∗∗*p* < 0.01.

Depletion of STAU1 or 2 in A549 cells using isoform-specific shRNAs (Figure S4B) increased perinuclear accumulation of *CEBPB* transcripts (Figures 4C and S4C). High-resolution imaging further showed that *CEBPB* mRNAs became more proximal to CK2α foci upon STAU1/2 silencing (Figure 4D), similar to the effect observed following UPF1 depletion (Figure 3A). Live-cell imaging of fluorescently tagged proteins demonstrated that STAU1 and STAU2 form perinuclear foci that extensively co-localize with CK2α on signaling endosomes (Figures 4E and S4D). Each protein localized perinuclearly when expressed individually, indicating that co-expression is not required for this distribution (Figure S4E). UPF1 also showed substantial overlap with CK2α, although some UPF1 foci were spatially distinct (Figure 4E).

Depletion of either STAU isoform induced senescence in a partially C/EBPβ-dependent manner (Figure S4F) and increased expression of pro-inflammatory SASP genes (Figure S4G), although to a lesser extent than observed with UPF1 depletion. Together, these findings indicate that STAU1/2 cooperate with UPF1 to promote *CEBPB* mRNA decay at or near CK2 signaling endosomes, thereby suppressing C/EBPβ activation in tumor cells.

### Identification of an STAU binding site adjacent to the GRE region

Comparison of human and mouse *CEBPB* 3′UTR sequences revealed that the region encompassing the GRE is highly conserved (Figure S5A). RNA secondary structure modeling (RNAfold^40^) predicted a stem-loop formed by pairing part of the GRE with an adjacent 5′ sequence (Figures 5A and S5B). A segment of this predicted double-stranded region is nearly identical between mouse and human homologs and shares similarity with a known STAU binding site (SBS) in the *ARF1* 3′UTR^39^ (Figure S5C). These observations suggested that a conserved SBS adjacent to the GRE may recruit STAU proteins to the 3′UTR to promote mRNA decay.

**Figure 5:**
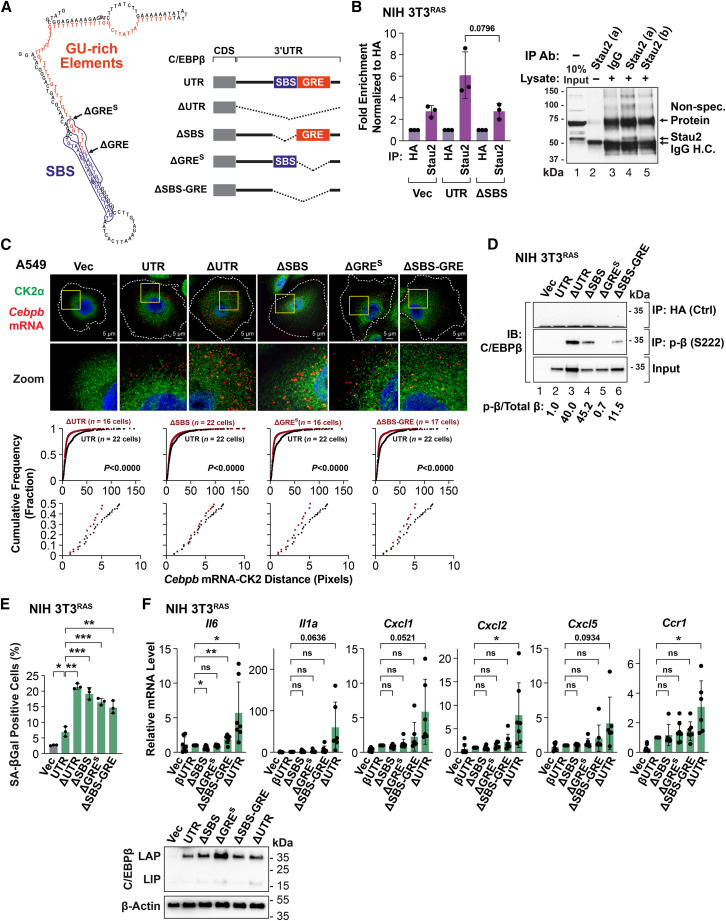
Staufen binds to an SBS element in the *Cebpb* 3′UTR that is required to suppress C/EBPβ phosphorylation by CK2 (A) An RNA secondary structure model (RNAfold^40^) of the murine *Cebpb* 3′UTR predicts a stem-loop feature formed by part of the G/U-rich sequence (red) paired with a 5′ adjacent sequence to create a putative SBS (purple). Right: diagram of *Cebpb*^*UTR*^, ΔUTR, ΔSBS, ΔGRE^S^, and ΔSBS-GRE constructs. (B) Evidence for Stau2 binding to the SBS region. nRIP assays were performed using Stau2 or HA control antibodies to assess *Cebpb* mRNA enrichment in 3T3^RAS^ cells expressing *Cebpb*^*UTR*^ or ΔSBS. Right: immunoblot confirming Stau2 IP using two different antibodies, denoted (a) and (b). (C) *Cebpb* mRNA FISH analysis of *Cebpb* 3′UTR mutants expressed in A549 cells, combined with CK2α IF imaging. The probe for murine *Cebpb* does not detect endogenous human *CEBPB* transcripts. Below: nearest neighbor analysis of *Cebpb* transcripts and CK2α IF signals, as described in Figure 3A. The lower graphs show expanded views of plot areas below 0.5 (*y* axis). (D) *Cebpb* constructs were expressed in 3T3^RAS^ cells and analysis of C/EBPβ phosphorylation on Ser222 was performed. Nuclear lysates were immunoprecipitated using the p-Ser222 antibody followed by immunoblotting for total C/EBPβ as described in Figure 3I. (E and F) Cells expressing the *Cebpb* constructs were analyzed for senescence (SA-βGal) (E) and expression of SASP genes (F). Levels of ectopically expressed C/EBPβ are shown in the immunoblot. Scale bar lengths are shown in the micrographs. *n* = number of cells or samples analyzed. Data are presented as mean ± SD. Error bars indicate SD. Statistical significance, except for the analysis in (C), was determined using Student’s paired *t* test. ∗*p* < 0.05, ∗∗*p* < 0.01, ∗∗∗*p* < 0.001; ns, not significant.

To test this hypothesis, we generated deletion mutants in murine *Cebpb* that lack the SBS, GRE, or SBS+GRE sequences (Figure 5A). The 5′ endpoint of the GRE deletion was designed to preserve the full SBS-like sequence and is referred to as ΔGRE^S^, distinguishing it from the previously described ΔARE (GRE) deletion^1^ corresponding to the mouse ΔGRE allele (see in the further section). To assess STAU binding, we expressed *Cebpb*^*UTR*^ (WT) and ΔSBS constructs in NIH 3T3^RAS^ cells and performed nRIP assays. *Cebpb*^*UTR*^ transcripts were enriched ∼6-fold with the STAU2 antibody relative to an HA antibody control (Figure 5B), whereas the ΔSBS mutation decreased STAU2 binding ∼55% (*p* = 0.08). These results indicate that STAU2 binding is at least partly dependent on the SBS element.

When expressed in A549 cells, *Cebpb*^*ΔUTR*^ mRNAs showed strong overlap with perinuclear CK2α, whereas *Cebpb*^*UTR*^ transcripts were excluded from this region (Figure 5C), consistent with Figure 3G. Deletion of the SBS and/or GRE increased localization of *Cebpb* transcripts within the CK2-rich perinuclear compartment, as quantified by nearest-neighbor analysis. In HEK293T cells co-expressing HRAS^G12V^, the *Cebpb*^*ΔUTR*^ and *Cebpb*^*ΔSBS*^ constructs showed elevated C/EBPβ Ser222 phosphorylation (Figure S5D). Similarly, in 3T3^RAS^ cells, all variants lacking the SBS—but not those lacking the GRE alone—exhibited increased p-Ser222 levels (Figure 5D).

Despite these differences, all constructs except *Cebpb*^*UTR*^ induced senescence (Figure 5E) and reduced focus formation (Figure S5E). Notably, only *Cebpb*^*ΔUTR*^ robustly activated SASP gene expression (Figure 5F). These findings indicate that the SBS element specifically suppresses CK2-dependent phosphorylation of C/EBPβ, whereas both the SBS and GRE are required to inhibit its pro-senescence activity, since deleting either motif produces a protein that induces senescence. In addition, a distinct 3′UTR element, separate from the SBS/GRE region, appears to restrain C/EBPβ activation of SASP genes.

### Oncogenic RAS induces senescence but not the SASP in *p19*^*Arf−/−*^;*Cebpb-GRE*^*Δ/Δ*^ MEFs

To investigate the relevance of UPA *in vivo*, we generated a mouse strain carrying a deletion of the *Cebpb* GRE region. This deletion was based on the original mapping of the GRE (ARE) motif^23^^,^^41^ and removes part of the sequence we now identify as an SBS (Figures S6A and 5A). Homozygous *GRE*^*Δ/Δ*^ animals were viable, fertile, and displayed no overt phenotypes. Aging studies revealed no differences in lifespan or disease susceptibility compared to *WT* animals (Figure S6B).

Because C/EBPβ UPA is inactivated during RIS but maintained in RAS-transformed tumor cells,^22^ and because loss of the *p19*^*Arf*^ tumor suppressor permits senescence bypass, we reasoned that the effects of the *GRE*Δ allele might be more evident in cells without *p19*^*Arf*^. To test this, we crossed the mutant strain to mice lacking *p19*^*Arf*^ and prepared MEFs of four genotypes: *WT*, *GRE*^*Δ/Δ*^, *p19*^*Arf−/−*^, and *p19*^*Arf−/−*^;*GRE*^*Δ/Δ*^. RAS-induced phosphorylation of C/EBPβ Ser222 was increased ∼2-fold in *p19*^*Arf−/−*^;*GRE*^*Δ/Δ*^ MEFs relative to *p19*^*Arf−/−*^ cells (Figure 6A), consistent with partial disruption of the SBS motif in the *GRE*Δ allele. These cells also formed fewer transformed foci despite comparable C/EBPβ expression (Figures 6B and 6C).

**Figure 6:**
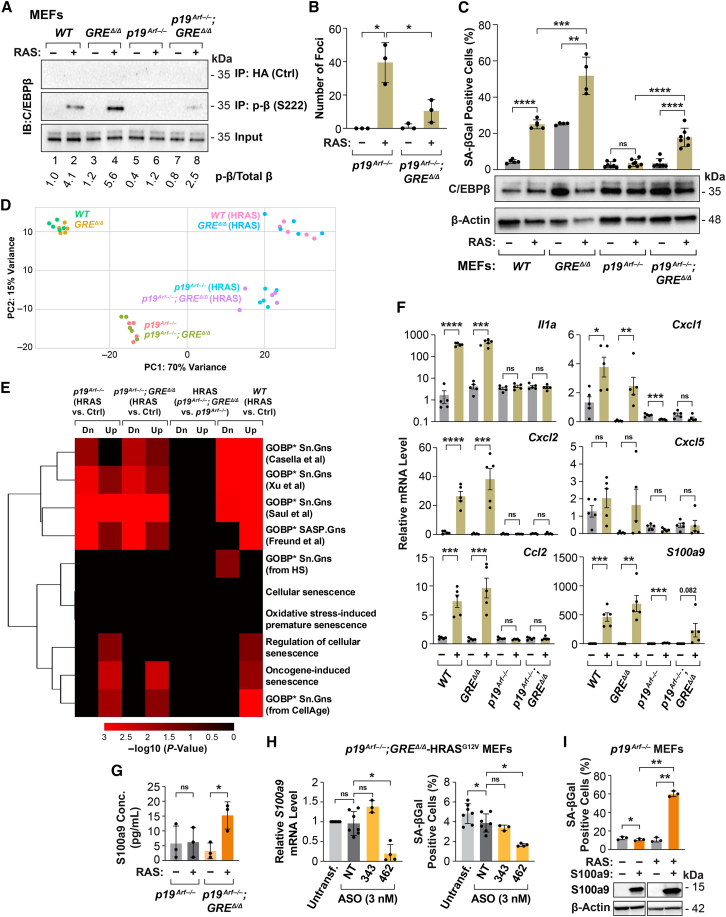
*p19*^*Arf−/−*^;*Cebpb-GRE*^*Δ/Δ*^ MEFs undergo RAS-induced senescence and express pro-senescent S100a9 but not canonical SASP genes (A) HRAS^G12V^-induced phosphorylation on C/EBPβ Ser222 is enhanced in *p19*^*Arf−/−*^;*GRE*^*Δ/Δ*^ MEFs. Nuclear lysates from low-passage *WT*, *GRE*^*Δ/Δ*^, *p19*^*Arf−/−*^, and *p19*^*Arf−/−*^;*GRE*^*Δ/Δ*^ MEFs (±HRAS^G12V^) were analyzed as described in Figure 5D. (B) Focus assays assessing HRAS^G12V^-induced neoplastic transformation of *p19*^*Arf−/−*^ and *p19*^*Arf−/−*^;*GRE*^*Δ/Δ*^ MEFs. Three independent MEF populations were analyzed for each genotype. (C) Senescence assays for each MEF population, ± HRAS^G12V^. The percentage of SA-βGal positive cells (*n* > 400 cells scored) was determined using 4–7 independent MEF cultures for each genotype. Immunoblots show C/EBPβ levels (p35 LAP isoform) in a representative image of MEFs. (D) Principal-component analysis (PCA) plot of RNA-seq data from MEF samples, ± HRAS^G12V^. Gene expression data were obtained from five independent MEF isolates per genotype. (E) RAS-induced genes in *p19*^*Arf−/−*^ and *p19*^*Arf−/−*^;*GRE*^*Δ/Δ*^ MEFs show similar associations with senescence markers. Genes up- or downregulated by HRAS^G12V^ (DESeq2-derived differentially expressed genes) in *WT* or mutant MEFs were analyzed for enrichment of senescence terms using GO biological process annotations. In some cases, GO databases were supplemented by published senescence gene signatures; these are designated GOBP∗ in the heatmap (see STAR Methods for full details and references). Red: significant enrichment; black: no enrichment. (F) HRAS^G12V^-expressing *p19*^*Arf−/−*^;*GRE*^*Δ/Δ*^ MEFs show minimal activation of canonical proinflammatory SASP genes but induce *S100a9*. RNAs from *WT*, *GRE*^*Δ/Δ*^, *p19*^*Arf−/−*^, and *p19*^*Arf−/−*^;*GRE*^*Δ/Δ*^ MEFs (±HRAS^G12V^) were analyzed by RT-qPCR for a panel of SASP genes. All values were normalized to the average of the *WT* controls (without HRAS^G12V^). Results for additional SASP genes are shown in Figure S6F. (G) S100a9 protein expression in *p19*^*Arf−/−*^ and *p19*^*Arf−/−*^;*GRE*^*Δ/Δ*^ MEFs (±HRAS^G12V^). S100a9 levels were analyzed in cell lysates using Luminex assays.^42^ (H) Depletion of *S100a9* in *p19*^*Arf−/−*^;*GRE*^*Δ/Δ*^ MEFs decreases HRAS^G12V^-induced senescence. Left: *S100a9* mRNA levels in cells treated with targeting and non-targeting ASOs (also see Figure S6G). Right: analysis of senescence in the same ASO-treated cells. (I) Ectopic expression of S100a9 in *p19*^*Arf−/−*^ MEFs induces senescence only in HRAS^G12V^-transduced cells. The immunoblot shows S100a9 protein levels. Data are presented as mean ± SD. Error bars indicate SD. Statistical significance was determined using unpaired (B, C, and F) or paired (G–I) Student’s *t* tests. ∗*p* < 0.05, ∗∗*p* < 0.01, ∗∗∗*p* < 0.001, ∗∗∗∗*p* < 0.0001; ns, not significant.

*GRE*^*Δ/Δ*^ MEFs exhibited elevated basal senescence relative to *WT* cells, indicating a predisposition to premature senescence, and showed further increases upon HRAS^G12V^ expression (Figures 6C and S6C). While *p19*^*Arf−/−*^ cells largely bypassed RIS, *p19*^*Arf−/−*^;*GRE*^*Δ/Δ*^-RAS MEFs displayed robust senescence, including SA-βGal staining and characteristic hypertrophic morphology (Figures 6C and S6C). These findings indicate that deletion of the GRE/SBS region restores the pro-senescence activity of C/EBPβ in RAS-expressing *p19*^*Arf*^-deficient cells.

To define the molecular basis of this phenotype, we performed bulk RNA-seq on MEFs of all four genotypes ± HRAS^G12V^ (Table S4). Replicates clustered tightly by genotype and treatment (Figure 6D). Notably, *GRE*^*Δ/Δ*^ samples clustered with *WT* cells, whereas the four categories determined by *p19*^*Arf*^ and HRAS^G12V^ status differed markedly. Thus, the *GRE*^*Δ*^ mutation has minimal effects on global gene expression. However, comparison of *p19*^*Arf−/−*^ and *p19*^*Arf−/−*^;*GRE*^*Δ/Δ*^ cells revealed a subset of RAS-responsive genes altered by the *GRE*^*Δ*^ mutation (Figure S6D and Table S5). GO analysis showed enrichment for senescence-associated pathways among RAS-regulated genes across all genotypes (Figure 6E and Table S6), and direct comparison between *p19*^*Arf−/−*^ and *p19*^*Arf−/−*^;*GRE*^*Δ/Δ*^ cells did not reveal distinct senescence gene signatures. Consistent with this, RIS signatures were comparable between *WT* and *GRE*^*Δ/Δ*^ cells (Figure S6E). Thus, despite divergent senescence phenotypes, *p19*^*Arf−/−*^ and *p19*^*Arf−/−*^;*GRE*^*Δ/Δ*^ cells exhibit broadly similar transcriptional responses to RAS.

Analysis of SASP gene expression revealed a striking dissociation between senescence and SASP activation. HRAS^G12V^ induced canonical SASP genes in *WT* and *GRE*^*Δ/Δ*^ MEFs but not in *p19*^*Arf−/−*^ cells (Figures S6F and S6G; Table S7). Importantly, *p19*^*Arf−/−*^;*GRE*^*Δ/Δ*^ cells also failed to activate most SASP genes despite undergoing senescence. RT-qPCR analysis of individual SASP genes confirmed that most were significantly induced by HRAS^G12V^ in *WT* and *GRE*^*Δ/Δ*^ MEFs but not in *p19*^*Arf−/−*^ or *p19*^*Arf−/−*^;*GRE*^*Δ/Δ*^ cells (Figure 6F and S6H). This phenotype parallels that observed in cells expressing *Cebpb* mutants lacking GRE and/or SBS elements (Figures 5E and 5F), supporting the existence of a distinct 3′UTR element that limits C/EBPβ-dependent activation of SASP genes.

One exception was *S100a9*, which was induced by RAS in *WT*, *GRE*^*Δ/Δ*^, and *p19*^*Arf−/−*^;*GRE*^*Δ/Δ*^ MEFs, but only weakly in *p19*^*Arf−/−*^ cells, correlating with senescence (Figures 6F and 6G). S100a9 is a non-canonical, Ca^2+^-binding pro-inflammatory cytokine that activates TLR4 and the inflammasome^43^ and has been shown to induce senescence in bone marrow mesenchymal cells.^44^ These findings suggested that S100a9 may act as a key effector of RIS in *p19*^*Arf−/−*^;*GRE*^*Δ/Δ*^ MEFs.

To test this hypothesis, we performed antisense oligo (ASO) knockdown of S100a9. One ASO candidate (ASO-462) efficiently reduced *S100a9* expression in *p19*^*Arf−/−*^;*GRE*^*Δ/Δ*^-HRAS^G12V^ MEFs (Figures S6I and 6H) and significantly decreased senescence compared to control ASOs (Figure 6H). Conversely, S100a9 overexpression increased senescence in *p19*^*Arf−/−*^-HRAS^G12V^ MEFs but not in non-transformed cells (Figure 6I). These findings indicate that S100a9 is necessary, but not sufficient, for RIS. Additional RAS-induced factors likely cooperate with S100a9 to drive senescence in *p19*^*Arf−/−*^;*GRE*^*Δ/Δ*^ cells.

### The *Cebpb GRE*^*Δ/Δ*^ mutation impedes progression to adenocarcinoma in a *Kras*^*G12D*^-driven model of lung cancer

To assess whether the *GRE*^*Δ*^ mutation affects tumorigenesis, we crossed *GRE*^*Δ/Δ*^ mice with the cancer-prone strain *Kras*^*LA2*^, which develops lung tumors with high penetrance.^45^ Pulmonary lesions in this model are predominantly benign adenomatous hyperplasias and alveolar adenomas, although a subset progresses to malignant ADCs. Cohorts of *Kras*^*LA2*^*/+* and *Kras*^*LA2*^*/+*;*GRE*^*Δ/Δ*^ mice were aged until tumor burden or other pathology necessitated euthanasia. Overall survival did not differ between genotypes (Figure 7A). Total lung tumor burden (sum of all tumor areas) showed a modest reduction in *GRE*^*Δ/Δ*^ mice (*p* = 0.09) (Figure 7B).

**Figure 7:**
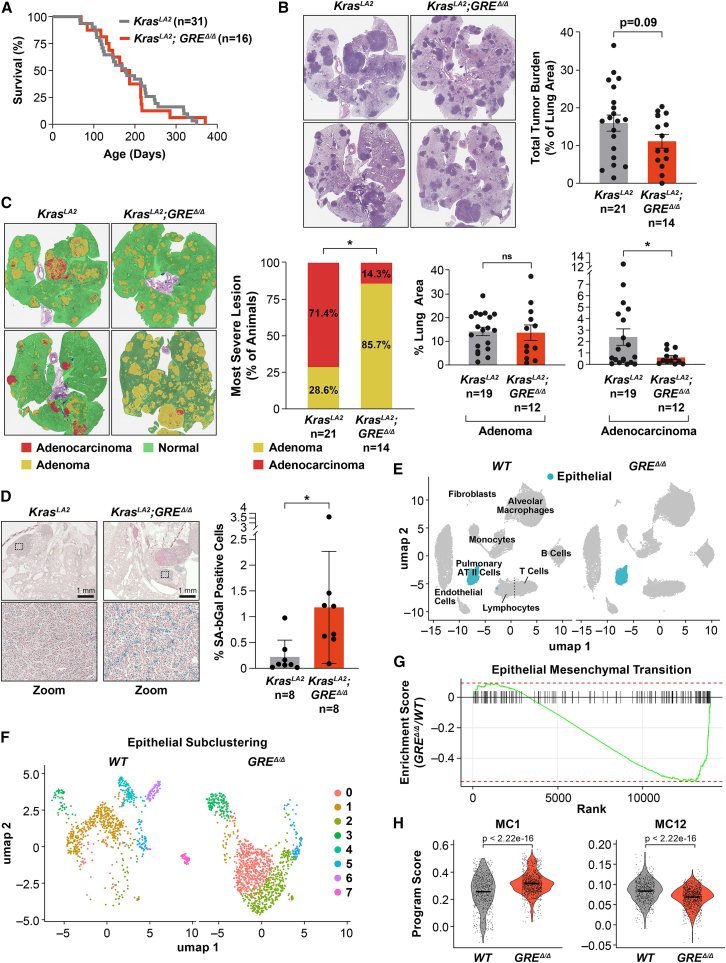
*Cebpb-GRE*^*Δ/Δ*^ mice display impaired progression to adenocarcinoma in a *Kras*^*G12D*^-driven model of lung cancer (A) Kaplan-Meier curves of *Kras*^*LA2/+*^ and *Kras*^*LA2/+*^;*GRE*^*Δ/Δ*^ mice. No significant difference in survival was observed (Log rank test; *p* = 0.84). (B) Left: two representative H&E images of tumor-bearing lungs from *Kras*^*LA2/+*^ and *Kras*^*LA2/+*^;*GRE*^*Δ/Δ*^ mice. Right: total lung tumor burdens (percent tumor area/animal) at clinical sacrifice. Tumor areas were determined using the HALO random forest tissue classifier. (C) Left: two representative HALO AI segmentations overlaid on H&E images from *WT* and *GRE*^*Δ/Δ*^ lungs (the same specimens as shown in B). HALO AI was used to identify and segment normal lung tissue (green), adenomas (yellow), and adenocarcinomas (red). Middle: Most severe tumor grades in *Kras*^*LA2/+*^ and *Kras*^*LA2/+*^;*GRE*^*Δ/Δ*^ mice from pathological examination of lesions. Data represent the percentage of animals in each group (Fisher’s exact test; *p* = 0.0016). Right: HALO AI quantification of lung tumor burdens: adenoma (left graph) and adenocarcinoma (right graph), showing percent lesion area per animal. (D) Senescent cells in *Kras*^*LA2*^ and *Kras*^*LA2*^;*GRE*^*Δ/Δ*^ tumors. Lung sections were stained for SA-βGal (left) and scanned images used to quantitate positive cells (right). Data are expressed as % positive cells in tumor areas per animal. Tumor grades could not be determined due to the limited quality of histopathological features in frozen sections. (E) Uniform manifold approximation and projection (UMAP) plots of scRNA-seq data from *Kras*^*LA2*^ and *Kras*^*LA2*^;*GRE*^*Δ/Δ*^ lung tumor samples (see Figure S7F), with annotated cell-type identities. Cells with epithelial gene signatures are highlighted in blue and map to cluster 10, corresponding to pulmonary alveolar type II (AT2) cells. (F) Sub-clustering of the AT2 epithelial compartment, split by genotype, reveals eight distinct subpopulations. (G) An epithelial-mesenchymal transition (EMT) gene signature is preferentially enriched in *WT* tumor cells. Ranked differentially expressed genes (*GRE*^*Δ/Δ*^ vs. *WT*) from the epithelial compartment were analyzed by gene set enrichment analysis (GSEA), identifying EMT as the only significantly enriched pathway. (H) Single-cell transcriptomes from epithelial populations of each genotype were compared to transcriptional programs corresponding to 12 mouse lung tumor cell states described by Marjanovic et al.^46^ Program scores for selected comparisons are shown, including Marjanovic clusters (MCs) 1 and 12, which illustrate key differences between genotypes. Scale bar lengths are shown in the micrographs. *n*, number of animals analyzed. Data are presented as mean ± SD. Error bars indicate SD. Statistical significance was determined using unpaired Student’s *t* test, except where indicated in (A) and (C). ∗*p* < 0.05; ns, not significant.

Histopathological evaluation revealed striking differences in tumor grade. ADCs were the most advanced lesions observed in 71.4% of *WT* mice but only 14.3% of *GRE*^*Δ/Δ*^ mice (*p* = 0.0016; Fisher’s exact test) (Figure 7C). Using a supervised, AI-based algorithm (HALO image analysis software; Indica Labs) trained on H&E images to distinguish benign adenomas from malignant lesions, we quantitated tumor areas by grade (Figure 7C). While the adenoma areas were similar between the two genotypes, ADC areas were nearly 4-fold greater in *WT* mice than in *GRE*^*Δ/Δ*^ animals (*p* = 0.0244). This difference was not attributable to age at sacrifice, as no correlation was observed between age and ADC burden in either genotype (Figure S7A). Similar numbers of male and female mice were analyzed in each cohort, and no sex-dependent differences were observed in survival, overall tumor burden, or ADC progression.

Ki67 staining within ADC regions was similar between the two genotypes (Figure S7B), indicating that tumors that escape the *GRE*^*Δ/Δ*^ blockade retain high proliferative capacity. Likewise, infiltration of M1 and M2 macrophages did not differ between *WT* and *GRE*^*Δ/Δ*^ tumors (Figure S7C). In contrast, quantification of SA-βGal-positive areas revealed significantly increased senescence in *GRE*^*Δ/Δ*^ lesions (Figure 7D), consistent with reduced progression to malignancy. *GRE*^*Δ/Δ*^ tumors also contained more S100a9-positive cells (Figure S7D), mirroring the phenotype observed in *p19*^*Arf−/−*^;*GRE*^*Δ/Δ*^-HRAS^G12V^ MEFs. Analysis of nuclear morphology using HALO-based segmentation showed similar nuclear size distributions in adenomas from both genotypes (Figure S7E), whereas *GRE*^*Δ/Δ*^ ADC lesions exhibited a reduced frequency of karyomegalic cells compared to *WT* tumors, supporting their impaired progression to advanced malignancy.

To further investigate the basis of the adenoma-locked *GRE*^*Δ/Δ*^ tumor phenotype, we performed single-cell RNA sequencing (scRNA-seq) of tumor-bearing lung tissue from *WT* and mutant mice. Dimensionality reduction identified 21 transcriptionally distinct clusters that were broadly conserved between genotypes (Figure S7F and Table S8). Several clusters corresponded to immune cell populations (Figure 7E), none of which differed significantly in abundance between *WT* and *GRE*^*Δ/Δ*^ samples (Table S8), indicating that the immune microenvironment is not substantially altered by the mutation.

Cluster 10 was annotated as pulmonary alveolar type II (AT2) cells, the cell of origin for most KRAS-driven lung cancers,^47^ and uniquely exhibited a strong epithelial gene signature (Figure 7E; Table S9). Transcriptome-based copy-number analysis revealed that this epithelial subset was aneuploid in both genotypes, consistent with a tumor cell population, whereas immune cell clusters remained diploid (Figure S7G). Sub-clustering of the epithelial compartment revealed eight distinct subpopulations that were largely segregated by genotype (Figure 7F and Table S10), demonstrating substantial transcriptional divergence between *WT* and *GRE*^*Δ/Δ*^ tumors.

To identify genotype-dependent differences in tumor cell transcriptional programs, we performed gene set enrichment analysis (GSEA) using ranked gene expression (*GRE*^*Δ/Δ*^ vs. *WT*) within the epithelial compartment. Among all pathways tested, epithelial-mesenchymal transition (EMT) was the only significantly enriched pathway (Figure 7G and Table S11). This program, a hallmark of advanced lung tumors,^46^ was preferentially enriched in *WT* tumor cells, consistent with increased transcriptional plasticity.

We further characterized tumor cell states by comparing epithelial populations to 12 mouse lung tumor transcriptional programs described by Marjanovic et al.^46^ (Figures 7H and S7H; Table S12). *GRE*^*Δ/Δ*^ cells showed significantly higher similarity to differentiated AT2-associated programs (Marjanovic clusters [MCs] 1 and 2). In contrast, *WT* tumor cells exhibited reduced AT2 program activity and increased similarity to more divergent transcriptional states, exemplified by MC 12. Together, these findings support a model in which *GRE*^*Δ/Δ*^ tumor cells are biased toward an AT2-like differentiation state, whereas *WT* tumors undergo partial transcriptional reprogramming characterized by reduced epithelial identity and acquisition of EMT-associated features.

## Discussion

UPA inhibits C/EBPβ activity in tumor cells through 3′UTR-mediated exclusion of *CEBPB* transcripts from the kinase-rich perinuclear region.^1^ We now identify PMD as a mechanism that eliminates *CEBPB* mRNAs from this compartment. Importantly, stabilization of *CEBPB* mRNA using the *Malat1* element was sufficient to override 3′UTR-mediated exclusion and restore perinuclear mRNA localization, C/EBPβ phosphorylation, and senescence. This provides direct evidence that localized mRNA decay is the key mechanism controlling C/EBPβ activity.

Two known mRNA decay proteins, UPF1 and STAU, play key roles in PMD. The *CEBPB* GRE and an adjacent SBS motif recruit HuR and STAU, respectively, to control mRNA localization. However, these two elements act by distinct mechanisms, as the SBS inhibits CK2-dependent phosphorylation of C/EBPβ whereas the GRE does not. PMD also appears to require SMG6 and SMG7 but not SMG5 or the UPF1 kinase, SMG1. This requirement distinguishes PMD from canonical NMD and is consistent with non-canonical UPF1-SMG6/SMG7 interactions, differentiating PMD from other UPF1-dependent decay pathways.^48^ Although PMD involves STAU, it is genetically separable from STAU-mediated decay, which requires SMG1.^49^ These findings support classification of PMD as a distinct, 3′UTR-directed perinuclear branch of UPF1-mediated mRNA decay.

The subcellular location of PMD is essential to suppress C/EBPβ phosphorylation in tumor cells, as its activating kinases CK2 and ERK reside on perinuclear endosomes.^18^ Localized *CEBPB* mRNA decay involves compartmentalization of UPF1 and STAU in this region, where they are inversely correlated with *CEBPB* transcripts. Depletion of UPF1 or STAU1/2 leads to co-localization of *CEBPB* transcripts with CK2 puncta. STAU1 and STAU2 strongly overlap with CK2, while UPF1 and CK2 partially co-localize, suggesting that *CEBPB* mRNA decay is specifically targeted to signaling endosomes. PMD could therefore be described as kinase-proximal mRNA decay (KPMD). The presence of STAU on CK2 endosomes provides a compelling explanation for increased phosphorylation on C/EBPβ Ser222 when the SBS motif is deleted, but not when the GRE alone is removed. Thus, despite their proximity, the SBS does not require the GRE for STAU2-mediated decay of *CEBPB* transcripts. The GRE likely suppresses C/EBPβ phosphorylation through a distinct perinuclear kinase, which remains to be identified.

The accumulation of *CEBPB* transcripts near CK2 endosomes following UPF1 or STAU depletion, or upon deletion of the 3′UTR, suggests the existence of an intrinsic targeting mechanism. This could involve a localization determinant within the coding region or a signal within the nascent C/EBPβ polypeptide that directs polysomes to CK2-containing endosomes. Such proximity implies a spatially coupled mechanism linking translation and phosphorylation (Figure S7I), whereby newly synthesized C/EBPβ is modified *in situ*. This model explains why C/EBPβ translated outside the perinuclear compartment remains underphosphorylated and less active. KPMD thus prevents kinase access to C/EBPβ, thereby suppressing senescence in tumor cells.

An unexpected finding was that the pro-senescence and SASP-inducing activities of C/EBPβ are inhibited by distinct 3′UTR elements. This is illustrated by the differing responses of transformed cells overexpressing *Cebpb*^*UTR*^ (neither senescence nor the SASP), *Cebpb*^*ΔUTR*^ (senescence and SASP), and ΔGRE and/or ΔSBS mutants (senescence only). Additional support comes from the senescence-prone but SASP-deficient phenotype of HRAS^G12V^-expressing *p19*^*Arf−/−*^;*GRE*^*Δ/Δ*^ MEFs. While the SBS/GRE region is required to inhibit senescence, its deletion does not activate SASP genes, implicating a separate regulatory element. Accordingly, HuR depletion induces senescence but only weakly activates the SASP (Figure S1B), whereas UPF1 depletion activates both programs, indicating that mRNA decay is central to both inhibitory mechanisms. UPF1 is downregulated in senescent fibroblasts expressing oncogenic RAS, consistent with prior observations in replicative senescence,^50^ but remains expressed in transformed *p19*^*Arf−/−*^ MEFs, supporting a key role for UPF1 in senescence bypass.

*GRE*^*Δ/Δ*^ mice exhibited no overt phenotypes, indicating that GRE-dependent UPA functions are not required for normal mouse development and physiology. However, in *Kras*^*G12D*^-driven lung tumorigenesis, the *GRE*^*Δ*^ allele strongly suppressed progression to ADC and increased senescence within tumors, paralleling observations in RAS-expressing *p19*^*Arf−/−*^;*GRE*^*Δ/Δ*^ MEFs. Formation of benign adenomas was unaffected, indicating that UPA becomes critical when tumors progress to carcinomas. Consistent with this model, scRNA-seq analysis revealed that *GRE*^*Δ/Δ*^ tumor cells are biased toward an AT2-like differentiation state, whereas *WT* tumors exhibit features of EMT-associated transcriptional reprogramming, thereby linking UPA-dependent regulation of C/EBPβ to tumor cell identity. Collectively, these findings provide the first evidence that UPA functions *in vivo*.

*Kras*^*LA2*^-driven lung ADCs exhibit elevated RAS signaling and increased p-ERK relative to adenomas.^51^ Hyperactive RAS activates C/EBPβ, which would impede progression to ADC in the absence of UPA. RAS signaling also engages pro-senescence pathways such as Arf/p53,^52^ creating selective pressure for loss of these tumor suppressors.^51^ The absence of p19^*Arf*^ may be linked to UPA function, consistent with our observation that the *GRE*^*Δ*^ mutation restores RIS in *p19*^*Arf−/−*^ MEFs (Figure 6C).

UPA likely regulates additional tumor suppressors, including p53, whose activity is also restrained by its 3′UTR in cancer cells.^53^ Thus, 3′UTR-directed mRNA decay may represent a broader mechanism by which transformed cells dampen tumor suppressor activity to evade senescence. Targeting UPA could therefore provide a novel therapeutic strategy to restore senescence in cancer cells.

### Limitations of the study

Although our study establishes KPMD as a mechanism to inhibit C/EBPβ-mediated senescence in tumors, some aspects of this process remain unresolved. While we identify UPF1 and STAU proteins as key components, the full complement of factors and 3′UTR regulatory sequences involved has yet to be defined. For example, we show that a distinct 3′UTR element suppresses SASP gene activation, but the specific sequence, the responsible RNA-binding protein(s), and the relevant downstream modifications remain to be determined. In addition, our mutational analysis focused on the murine 3′UTR and was not extended to the human transcript. Finally, while our findings suggest potential therapeutic relevance, development of strategies to selectively activate pro-senescence proteins such as C/EBPβ has not been explored and will require further investigation.

## Resource availability

### Lead contact

Further information and requests for resources and reagents should be directed to, and will be fulfilled by, the lead contact, Peter F. Johnson (johnsope@mail.nih.gov).

### Materials availability

The unique materials generated in this study will be made available upon request to the lead contact.

### Data and code availability

• Large datasets have been deposited in the appropriate repositories. Proteomics data, including raw mass spectrometry data, have been deposited in MassIVE. Raw build RNA-seq data, including individual sample information, have been deposited in the NCBI Sequence Read Archive (SRA). Raw single-cell RNA-seq data have been deposited in NCBI GEO. Accession numbers for all datasets are listed under deposited data in the key resources table.
• Stepwise instructions and ImageJ macros for executing image analysis methods (relative nuclear proximity index and nearest neighbor analysis; STAR Methods) will be provided upon request.

## Acknowledgments

We thank Kristen Lynch for insightful comments on the PMD mechanism; Eugene Valkov for suggesting the *Malat1* experiment and providing constructs; Dominic Esposito for RAS vectors; Allen Kane and Joseph Meier (Scientific Publications, Graphics and Media, Leidos Biomedical Research, Inc., Frederick National Laboratory for Cancer Research) for preparing figures; the NCI CCR Sequencing Facility lab and bioinformatics team for providing sequencing and primary analysis support; Holly Morris for animal technical support; and Andrew Warner and Leticia Arguera (Molecular Histopathology Laboratory, Laboratory Animal Sciences Program, Frederick National Laboratory for Cancer Research) for single-cell isolation from lung tumor samples. This research was supported in part by the 10.13039/100031022Center for Cancer Research, National Cancer Institute, National Institutes of Health, and federal funds from the 10.13039/100000054National Cancer Institute, National Institutes of Health, under contract no. 75N91019D00024. The contributions of NIH authors were made as part of their official duties as NIH federal employees, are in compliance with agency policy requirements, and are considered works of the United States Government. The findings and conclusions presented in this paper are those of the authors and do not necessarily reflect the views of the NIH or the U.S. Department of Health and Human Services.

## Author contributions

J.S. and P.F.J. conceived the study and designed experiments. J.S., N.A., S.B., A.D., L.H., M. Yang, B.K., N.M., S.M., and P.F.J. performed experiments and/or data analysis. J.S., S.B., and P.F.J. performed image analysis; D.A.S. analyzed microscopic images using the IMARIS software package; S.L. developed image analysis pipelines and associated Fiji macro scripts. T.A. and B.T.L. performed MS experiments and/or data analysis. K.S., N.M., and M. Yang conducted animal experiments and genotyping. B.K. performed histopathological analysis of lung tumor samples. M. Yi and M.G. performed bioinformatic analysis of bulk RNA-seq data and single-cell RNA-seq data, respectively. L.T. provided key resources. J.S. and P.F.J. wrote the manuscript with input from all authors.

## Declaration of interests

The authors declare no competing interests.

## Declaration of generative AI and AI-assisted technologies in the writing process

ChatGPT was used for final text editing to improve clarity and succinctness. AI-based HALO image analysis tools were used as described in STAR Methods.

## STAR★Methods

### Key resources table

**Table:** 

REAGENT or RESOURCE	SOURCE	IDENTIFIER
**Antibodies**		

β-Actin	Santa Cruz Biotechnology	Cat#sc-1616; RRID:AB_630836
β-Actin	Santa Cruz Biotechnology	Cat#sc-47778; RRID:AB_626632
Anti-mouse Alexa Fluor 488	ThermoFisher Scientific	Cat#A21202; RRID:AB_141607
Anti-mouse Alexa Fluor 568	ThermoFisher Scientific	Cat#A10037; RRID:AB_11180865
Anti-mouse Alexa Fluor 647	ThermoFisher Scientific	Cat#A21236; RRID:AB_2535805
Anti-mouse horseradish peroxidase-conjugated antibody	Promega	Cat#W4028
Anti-rabbit Alexa Fluor 488	Abcam	Cat#ab150077; RRID:AB_2630356
Anti-rabbit Alexa Fluor 647	Abcam	Cat#ab150075; RRID:AB_2752244
Anti-rabbit horseradish peroxidase-conjugated antibody	Promega	Cat#W4018
Anti-Rabbit IgG (Light Chain)-HRP	Jackson Laboratories	Cat#211-032-171
CD86	Cell Signaling Technology	Cat#19589; RRID:AB_2892094
CD206	Cell Signaling Technology	Cat#24595; RRID:AB_2892682
C/EBPβ	Abcam	Cat#ab32358; RRID:AB_726796
C/EBPβ	NSJ Bioreagents	Cat#R31588
C/EBPβ (Mouse mAb)	Santa Cruz Biotechnology	Cat#sc-7962; RRID:AB_626772
phospho-C/EBPβ (Ser222/223; mouse/rat sites)	Inhouse	Basu et al.^18^
CSNK2A1 (CK2a)	Abcam	Cat#ab10466; RRID:AB_297210
CSNK2A1 (CK2a)	Santa Cruz Biotechnology	Cat#sc-365762; RRID:AB_10844972
GFP	Santa Cruz Biotechnology	Cat#sc-8334; RRID:AB_641123
HuR	Santa Cruz Biotechnology	Cat#sc-5261; RRID:AB_627770
IgG XP® Isotype Control (rabbit monoclonal)	Cell Signaling Technology	Cat#3900
Ki67	Cell Signaling Technology	Cat#12202
Lamin A/C	Cell Signaling Technology	Cat#2032; RRID:AB_213627
S100A9 (Rabbit mAb)	Cell Signaling Technology	Cat#D3U8M
S100A9	Cell Signaling Technology	Cat#73425; RRID:AB_2799839
SMG1	Santa Cruz Biotechnology	Cat#sc-374557; RRID:AB_11008478
SMG5	ProteinTech	Cat#12694-I-AP
SMG6	ThermoFisher Scientific	Cat#PA5-60165; RRID:AB_2647637
SMG7	GeneTex	Cat#GTX121320; RRID:AB_11164524
STAU1	Abcam	Cat#ab137100; RRID:AB_3675485
STAU2	Sigma	Cat#HPA019155; RRID:AB_1857565
α-Tubulin	Santa Cruz Biotechnology	Cat#sc-8035; RRID:AB_628408
UPF1	Cell Signaling Technology	Cat#12040; RRID:AB_2797806
UPF1	Santa Cruz Biotechnology	Cat#sc-393594; RRID:AB_2819146
phospho-UPF1 (Ser1127)	MilliporeSigma	Cat#07-1016

**Bacterial and virus strains**		

DH5m	ThermoFisher Scientific	Cat#18265017
Stbl3	ThermoFisher Scientific	Cat#C737303

**Biological samples**		

Lung tumor samples from Kras^G12D−LA2/+^*WT* and *GRE*^*Δ/Δ*^ mice	Inhouse	This study
Mouse embryo fibroblasts (MEFs) from *WT*, *GRE*^*Δ/Δ*^, *p19*^*Arf−/−*^ and *p19*^*Arf−/−*^;*GRE*^*Δ/Δ*^ mice	Inhouse	This study

**Chemicals, peptides, and recombinant proteins**		

25 cm Acclaim PepMap C18 column	ThermoFisher Scientific	Cat#Easy nLC 1200
ACK Lysis Buffer	Gibco	Cat#A10492-01
Actinomycin D	MilliporeSigma	Cat#A9415
Bovine Serum Albumin (BSA)	MilliporeSigma	Cat#A6003
Benzonase	MilliporeSigma	Cat#E1014
Collagenase	MilliporeSigma	Cat#C6885
Dulbeccos Modified Eagle Medium (DMEM)	Life Technologies	Cat#10569-010
Dynabeads® MyOne™ Streptavidin C1	ThermoFisher Scientific	Cat#65001
Dynabeads™ Protein G for Immunoprecipitation	ThermoFisher Scientific	Cat#10004D
Fluorescent Opal520 reporter dye	Akoya Biosciences	Cat#FP1487001KT
Fluorescent Opal570 reporter dye	Akoya Biosciences	Cat#FP1488001KT
Fluorescent Opal690 reporter dye	Akoya Biosciences	Cat#FP1497001KT
G418	Invivogen	Cat#Ant-gn-5
Hyaluronidase	MilliporeSigma	Cat#H3884
Hygromycin	Invivogen	Cat#ant-hg-5
Micrococcal Nuclease (MNase)	ThermoFisher Scientific	Cat#88216
Normocin	Invivogen	Cat#ant-nr-2
μ-Slide VI 0.4	Ibidi	Cat#80606
4X Laemmli sample buffer	Bio-Rad Laboratories	Cat#1610747
Opti-MEM medium	Life Technologies	Cat#31985070
Phosphatase Inhibitor Cocktail Set I	MilliporeSigma	Cat#524624
Phosphatase Inhibitor Cocktail Set II	MilliporeSigma	Cat#524625
Protease Inhibitor Cocktail Set I	MilliporeSigma	Cat#539131
Protein A, HRP conjugate	MilliporeSigma	Cat#18-160
Primocin	Invivogen	Cat#ant-pm-2
puromycin	Invivogen	Cat#ant-pr-5b
RNase inhibitor	Life Technologies	Cat#AM2696
RNAiMAX transfection reagent	Life Technologies	Cat#13778150
RPMI medium	Life Technologies	Cat#72400120
SYBR Green	Bio-Rad Laboratories	Cat#1725275
SUPERase-In RNase Inhibitor (20 U/μL)	ThermoFisher Scientific	Cat#AM2696
Trypsin Solution	MilliporeSigma	Cat#T9935
RNase A	ThermoFisher Scientific	Cat#R1253
Yeast tRNA	ThermoFisher Scientific	Cat#AM7119

**Critical commercial assays**		

Acclaim PepMap C18 column (Easy nLC 1200)	ThermoFisher Scientific	Cat#164199
Bond Polymer Refine Detection Kit	LeicaBiosystems	Cat#DS9800
Click-iT™ Nascent RNA Capture Kit	ThermoFisher Scientific	Cat#C10365
Duolink® *In Situ* PLA® Probe Anti-Rabbit PLUS	MilliporeSigma	Cat#DUO92002-30RXN
Duolink® *In Situ* PLA® Probe Anti-Mouse MINUS	MilliporeSigma	Cat#DUO92004-30RXN
Duolink® *In Situ* Detection Reagents Green	MilliporeSigma	Cat#DUO92014-30RXN
Duolink® *In Situ* Wash Buffers, Fluorescence	MilliporeSigma	Cat#DUO82049-4L
Epitope Retrieval 1 (Citrate)	Cell Signaling Technology	Cat#12202
GeneJet RNA purification kit	ThermoFisher Scientific	Cat#K0732
Luminex Discovery Assay Kit	R&D Systems	Cat#LXSAMSM
Maxima First Strand cDNA Synthesis Kit for RT-qPCR, with dsDNase	ThermoFisher Scientific	Cat#K1672
Protein assay dye reagent	Bio-Rad Laboratories	Cat#5000006
QiaShredder	Qiagen	Cat#79656
Quanti-Gene ViewRNA ISH Cell Assay	ThermoFisher Scientific	Cat#QVC0001
Senescence detection kit	Sigma-Aldrich	Cat#QIA117
SHIELD	LifeCanvas Technology	Cat#250 mL
SuperSignal™ West Dura Extended Duration Substrate	ThermoFisher Scientific	Cat#34076
SuperSignal™ West Pico PLUS Extended Duration Substrate	ThermoFisher Scientific	Cat#34579
TranscriptAid T7 High Yield Transcription Kit	ThermoFisher Scientific	Cat#K0441
TYPE 1 MOUSE *Cebpb* CCAAT/enhancer binding protein beta RNA ISH probe	ThermoFisher Scientific	Cat#009883 VB1-10094
TYPE 1 HUMAN *CEBPB* CCAAT/enhancer binding protein beta RNA ISH probe	ThermoFisher Scientific	Cat#005194 VA1-18129
X-tremeGENE HP cell transfection kit	ThermoFisher Scientific	Cat#50-100-3368
QuantiGene ViewRNA ISH Cell Assay	ThermoFisher Scientific	Cat#QVC0001

**Deposited data**		

Proteomics data (raw mass spectrometry data)	MassIVE	MSV0000999696
Raw bulk RNA-seq data	NCBI Sequence Read Archive (SRA)	BioProject accession PRJNA1347845
Raw bulk RNA-seq data (individual sample information)	NCBI Sequence Read Archive (SRA)	BioSample accessions SAMN52874904–SAMN52874943
Raw single cell RNA-seq data	NCBI GEO	GSE328098

**Experimental models: Cell lines**		

NIH 3T3	ATCC	Cat#CRL-1658
A549	ATCC	Cat#CCL-185
PANC-1	FNLCR/RAS Initiative	N/A
SW-900	FNLCR/RAS Initiative	N/A
HEK 293T	ATCC	Cat#CRL-3216
Mouse embryonic fibroblasts (MEFs)	Inhouse	This study

**Experimental models: Organisms/strains**		

Mouse: C57Bl/6Ncr	The Jackson Laboratory	Cat#MGI:2160593
Mouse: 129/SV	The Jackson Laboratory	Cat#MGI:2161069
Mouse: *GRE*^*flox*^	Inhouse	This study
Mouse: *GRE*^*Δ*^	Inhouse	This study
Mouse: p19^Arf−/−^ (CdKn2a-Arf) B6.129	NCI-Frederick MMRRC Repository	N/A
Mouse: ACTB-Cre C57Bl/6	Mark Lewandoski Lab	Lewandoski et al.^54^
Mouse: Kras^G12D−LA2/+^ 129/SV	Tyler Jacks Lab	Johnson et al.^45^

**Oligonucleotides**		

See Table S13		

**Recombinant DNA**		

pBabe (puro)	Addgene	Cat#1764
pBabeβUTR (puro)	Inhouse	Basu et al.^1^
pBabeΔUTR (puro)	Inhouse	Basu et al.^1^
pBabeβUTR-MALAT-scrbl (puro)	Inhouse	CCTGGTGATGTGTTGTGTT CTTGTACTCTGATAGCTTC GTGCTGCTGTTGTATCTTA CTATAGTGTTGGCATCGG CACCCATGTCATGGTTAG TATTCTGATCGCTCAGGT CCTTAGATAGGATGCCTT GCACGAGACGTGATACA ATCTTCGTTCTAATACAA TCAGAAGATA
pBabeβUTR-MALAT (puro)	Inhouse	GATTCGTCAGTAGGGT TGTAAAGGTTTTTCTTT TCCTGAGAAAACAACC TTTTGTTTTCTCAGGTT TTGCTTTTTGGCCTTT CCCTAGCTTTAAAAA AAAAAAAGCAAAAGA CGCTGGTGGCTGGC ACTCCTGGTTTCCAG GACGGGGTTCAAGT CCCTGCGGTGTCTT TGCTT
pBabeΔSBS (puro) (***BamHI site*** at deletion junction)	Inhouse	CACACGTGTAAC ***GGATCC***GTTTTT GGTTTT
pBabeΔGRE (puro)	Inhouse	3′UTR nucleotides 308–409 deleted, with *LoxP* site nserted at junction
pBabeΔGRE^short^ (puro)	Inhouse	GGGGTTGTTGAT***GGATCC***TATTAT ATAAAA
pBabeΔSBS-GRE (puro)	Inhouse	CACACGTGTAAC***GGATCC***TATTAT ATAAAA
pSirenRetroQ-puro	Clontech/Takara	Cat#631526
pSirenRetroQ-puro mouse shAnxa1	Inhouse	AGCAACCATCATTGACATTCT
pSirenRetroQ-puro mouse shAnxa2	Inhouse	CTTCGATGCTGAGAGGGATGC
pSirenRetroQ-puro mouse shCelf1	Inhouse	GAGCCAACCTGTTCATCTA
pSirenRetroQ-puro mouse shDhx9	Inhouse	GCCAGAGACTTTGTTAACTATT (Luo et al.^35^)
pSirenRetroQ-puro mouse shFmr1	Inhouse	GCATGTGATGCTACGTATA
pSirenRetroQ-puro mouse shFubp1	Inhouse	ATACAGATAGCACCTGATAGT
pSirenRetroQ-puro mouse shFubp2	Inhouse	GGGACACCATGATCTGAATGT
pSirenRetroQ-puro mouse shFubp3	Inhouse	GGATTAGTCCAGAAAGAGC
pSirenRetroQ-puro mouse shHnrnpa2b1	Inhouse	CGTGCTGTAGCAAGAGAGG (Chen et al.^55^)
)pSirenRetroQ-puro mouse shHnrnpd	Inhouse	GACGCCAGTAAGAACGAGG
pSirenRetroQ-puro mouse shIgf2bp1	Inhouse	CCGGGAGCAGACCAGGCAA (Weidensdorfer et al.^56^)
pSirenRetroQ-puro mouse shIgf2bp2	Inhouse	GGCATCAGTTTGAGGACTA (Boudoukha et al.^57^)
pSirenRetroQ-puro mouse shIgf2bp3	Inhouse	GGGAAGAATTTATGGAAAA
pSirenRetroQ-puro mouse shIlf2	Inhouse	CCATTTGGACATCAAGGTG
pSirenRetroQ-puro mouse shIlf3	Inhouse	GGACGGACAGAAGTTTCAAGG
pSirenRetroQ-puro mouse shMapre1	Inhouse	GAAGAAAGTGAAATTCCAAGC
pSirenRetroQ-puro mouse shNono	Inhouse	GACCTTTACACAGCGTAGC (Yadav et al.^58^)
pSirenRetroQ-puro mouse shPlec	Inhouse	GGCAGGACCGTGACCATCT
pSirenRetroQ-puro mouse shStrbp	Inhouse	GTCTATAGGTACTTGTAAT
pSirenRetroQ-puro mouse shUpf1	Inhouse	GATGCAGTTCCGTTCCATC
pSIRIP-puro shRNA p19Arf-2	Addgene	Cat#14090
pSuperRetro-neo	OligoEngine	Cat#VEC-PRT-0004
pSuperRetro-neo shCebpb-1	Inhouse	GATGTTCCTGCGGGGTTGT (Sebastian et al.^28^)
pSuperRetro-neo shCEBPB	Inhouse	GAAGAAACGTCTATGTGTA (Sebastian et al.^28^)
pSuperRetro-neo shELAVL1	Inhouse	GAGGCAATTACCAGTTTCA (Basu et al.^1^)
CMV13p>control (hygro)	Protein Expression Lab (FNLCR)	10449-M03-665
CMV51p>Hs.HRAS G12V	Protein Expression Lab (FNLCR)	R700-M18-665
pMD2.G	Addgene	Cat#12259
pMDLg/pRRE	Addgene	Cat#12251
pRSV/Rev	Addgene	Cat#12253
pReceiver-Lv123-CSNK2A1-eYFP (puro)	Genecopoeia	Cat#EX-T0147-Lv123
pReceiver-Lv216-Csnk2a1-mCherry (puro)	Genecopoeia	Cat#EX-Mm01978-Lv216
pReceiver-Lv155-STAU1-mCherry (*neo*)	Genecopoeia	Cat#EX-U1331-Lv155
pReceiver-Lv123-STAU2-eYFP (puro)	Genecopoeia	Cat#EX-Z7308-Lv123
pReceiver-Lv130-STAU2-mCherry (puro)	Genecopoeia	Cat#EX-Z7308-Lv130
pReceiver-Lv127-UPF1-eCFP (puro)	Genecopoeia	Cat#EX-Z0991-Lv127
pReceiver-Lv130-UPF1-mCherry (puro)	Genecopoeia	Cat#EX-Z0991-Lv130
S100A9 ORF expression clone (puro)	Genecopoeia	Cat#EX-Mm05072-Lv181
MISSION® pLKO.1-puro eGFP shRNA Control Plasmid	MilliporeSigma	Cat#SHC005
MISSION® pLKO.1-puro ELAVL1 shRNA Plasmid (1)	MilliporeSigma	Cat#TRCN0000276186
MISSION® pLKO.1-puro ELAVL1 shRNA Plasmid (2)	MilliporeSigma	Cat#TRCN0000276129
MISSION® pLKO.1-puro SMG1 shRNA Plasmid (1)	MilliporeSigma	Cat#TRCN0000196274
MISSION® pLKO.1-puro SMG1 shRNA Plasmid (2)	MilliporeSigma	Cat#TRCN0000244939
MISSION® pLKO.1-puro SMG5 shRNA Plasmid (1)	MilliporeSigma	Cat#TRCN0000421114
MISSION® pLKO.1-puro SMG5 shRNA Plasmid (2)	MilliporeSigma	Cat#TRCN0000130800
MISSION® pLKO.1-puro SMG6 shRNA Plasmid (1)	MilliporeSigma	Cat#TRCN0000040014
MISSION® pLKO.1-puro SMG6 shRNA Plasmid (2)	MilliporeSigma	Cat#TRCN0000419520
MISSION® pLKO.1-puro SMG7 shRNA Plasmid (1)	MilliporeSigma	Cat#TRCN0000292264
MISSION® pLKO.1-puro SMG7 shRNA Plasmid (2)	MilliporeSigma	Cat#TRCN0000292266
MISSION® pLKO.1-puro STAU1 shRNA Plasmid (1)	MilliporeSigma	Cat#TRCN0000159875
MISSION® pLKO.1-puro STAU1 shRNA Plasmid (2)	MilliporeSigma	Cat#TRCN0000164920
MISSION® pLKO.1-puro STAU2 shRNA Plasmid (1)	MilliporeSigma	Cat#TRCN0000219963
MISSION® pLKO.1-puro STAU2 shRNA Plasmid (2)	MilliporeSigma	Cat#TRCN0000102356
MISSION® pLKO.1-puro UPF1 shRNA Plasmid (1)	MilliporeSigma	Cat#TRCN0000413098
MISSION® pLKO.1-puro UPF1 shRNA Plasmid (2)	MilliporeSigma	Cat#TRCN0000022254
MISSION® pLKO.1-puro UPF1 shRNA Plasmid (3)	MilliporeSigma	Cat#TRCN0000022257
MISSION® pLKO.1-puro UPF1 shRNA Plasmid (4)	MilliporeSigma	Cat#TRCN0000022255
MISSION® pLKO.1-puro Upf1 shRNA Plasmid (1)	MilliporeSigma	Cat#TRCN0000274484
MISSION® pLKO.1-puro Upf1 shRNA Plasmid (2)	MilliporeSigma	Cat#TRCN0000009664

**Software and algorithms**		

Cell Ranger (v9.0.1)	10x Genomics	
clusterProfiler package (v 4.12.6)	Xu et al.^59^	
CopyKAT (v1.1.0)	https://github.com/navinlabcode/copykat	Gao et al.^60^
DAVID (2021 Release)	Place et al.^43^	
DESeq2 (v1.38.3)	R Bioconductor package	Love et al.^61^
DoubletFinder (v2.0.4)	McGinnis et al.^62^	
FindAllMarkers, FindNeighbors, FindClusters	Seurat package (v5.4.0)	Hao et al.^63^
fgsea package (v1.30.0)	Korotkevich et al.^64^	
Gene Ontology databases	www.geneontology.org/	
ggplot2 (v3.5.0)	R Project for Statistical Computing	https://www.r-project.org/
GraphPad Prism version 10.2.1 for Windows	GraphPad Software, Inc.	
HALO imaging analysis software v3.3.2541.300	Indica Labs	
Harmony (v1.2.4)	Korsunsky et al.^65^	
ImageJ	Gilles et al.^66^	
Imaris software version 9.8	Bitplane	
pheatmap (v1.0.12)	R Project for Statistical Computing	https://www.r-project.org/
PPEP	Inhouse pathway analysis method	Yi et al.^67^
Presto package (v1.0.0)	https://github.com/immunogenomics/presto	Korsunsky et al.^68^
Relative nuclear proximity index	Inhouse ImageJ-based tool	Basu et al.^36^
scType	Ianevsky et al.^69^	
Seurat package (v5.4.0)	RStudio (v4.4.0)	Hao et al.^63^
Zen Black and Zen Blue imaging software	Zeiss	

### Experimental model and study participant details

#### Animal studies

All animals were maintained in compliance with National Institutes of Health animal guidelines, and experimental procedures were approved by the Animal Care and Use Committee, National Cancer Institute at Frederick, under protocol numbers 21–306 and 21–319. *p19*^*Arf−/−*^ (*CdKn2a-Arf*) mice were obtained from the NCI-Frederick repository on a mixed background (B6.129). The strain was continuously backcrossed to C57Bl/6Ncr and maintained as homozygous stocks. *Cebpb-GRE*^*flox*^ mice were generated inhouse by injection of ES cells containing the targeted *GRE*^*flox*^ allele (*LoxP* sites flanking the 102-nt mouse *Cebpb* GRE region; 3′UTR nucleotides 308–409) into C57BL/6Ncr mouse blastocysts. The germline *GRE*^*Δ*^ allele was generated by mating *GRE*^*flox*^ mice with *ACTB-Cre* mice.^54^ *GRE*^*Δ*^ mice were then backcrossed to *WT* C57BL/6Ncr animals and maintained as a heterozygous stock. *p19*^*Arf−/−*^;*GRE*^*Δ/+*^ strain (C57BL/6Ncr) was obtained by intercrossing *p19*^*Arf−/−*^ and *GRE*^*Δ/+*^ mice and maintained by *p19*^*Arf−/−*^;*GRE*^*Δ/+*^ crosses. *Kras*^*G12D-LA2/+*^ mice^45^ were maintained by crossing to *WT* (129/SV). To obtain the *Kras*^*LA2/+*^;*GRE*^*Δ/+*^ strain, *Kras*^*LA2/+*^ (129/SV) and *GRE*^*Δ/+*^ (C57BL/6Ncr) mice were crossed. The mixed B6.129 background F1 progeny were bred (*Kras*^*LA2/+*^;*GRE*^*Δ/+*^ x *Kras*^*+/+*^;*GRE*^*Δ/+*^) to produce experimental animals. Mouse embryonic fibroblasts (MEFs) were prepared from embryonic day 13.5 (E13.5) embryos of the indicated genotypes. Male and female mice were used for lung tumor studies. Approximately equal numbers of animals of each sex were included in both experimental groups. Survival, total tumor burden, and adenocarcinoma progression were analyzed by sex, and no significant sex-dependent differences were observed. Data from male and female mice were therefore pooled for all subsequent analyses.

#### Cell line-derived xenografts (CDX)

Six-to eight-week-old Balb/c athymic nude mice were randomly grouped. The mice were warmed with an incandescent lamp to induce vasodilation, placed in a commercial restraint device, and injected via the lateral tail vein with 1 × 10^6^ A549 cells or derivatives, suspended in 100 μL of phosphate buffer saline (PBS). Animals were euthanized at 7 weeks post-injection using CO_2_ inhalation. The entire lung and bronchi were harvested and lung nodules were counted and measured. The lungs were inflated using 10% Neutral Buffered Formalin (NBF) before placing them into the same fixative. To ensure proper fixation stability, after five days in fixative, the tissues were placed into 70% ethanol.

### Method details

#### Cell lines

Mouse NIH 3T3 and NIH 3T3^RAS^ fibroblasts (embryonic mouse, sex not determined) were cultured in DMEM supplemented with 10% calf serum. Mouse embryonic fibroblasts (MEFs), human A549 (male), PANC-1 (male), SW-900 (male), GP2 and HEK 293T (both female) cell lines were cultured in DMEM supplemented with 10% fetal bovine serum (FBS). Primary MEFs were generated from littermate embryos without selection for sex. All cell lines were cultured with 100 μg/mL Primocin, maintained at subconfluency and grown in a humidified incubator at 37 °C with 5% CO_2_. Cells infected with retroviral/lentiviral plasmids were selected with 2 μg/mL puromycin for 2 days, 100 μg/mL hygromycin for 4 days or 800 μg/mL G418 for 7 days, as appropriate.

#### Plasmids

Oligonucleotides encoding short hairpin transcripts targeting mouse *Anxa1*, *Anxa2*, *Celf1*, *Dhx9*, *Fmr1*, *Fubp1*, *Fubp2*, *Fubp3*, *Hnrnpa2b1*, *Hnrnpd*, *Igf2bp1*, *Igf2bp2*, *Igf2bp3*, *Ilf2*, *Ilf3*, *Mapre1*, *Nono*, *Plec*, *Strbp* and *Upf1* were inserted between the BamHI and EcoRI sites of the knockdown vector pSirenRetroQ-puro. The knockdown vectors for mouse *Cebpb* (Cebpb1 shRNA) and human *CEBPB* have been described.^28^ The expression vectors pcDNA3.1(−)-β^UTR^, pcDNA3.1(−)-β^ΔUTR^, pBabe-β^UTR^, pBabe-β^ΔUTR^, and knockdown vector pSuperRetro-shElavl have been described.^1^ Lentiviral plasmids CMV51p-HRAS^G12V^ (hygro) and control CMV13p (hygro) were used for RAS transduction experiments. The triple helix sequence from mouse noncoding RNA *Malat1*^37^ and its control scrambled sequence were inserted into the 3′ end of pBabe-βUTR using synthetic oligos harboring PacI and SbfI restriction sites. The *Δ*SBS, *Δ*GRE^S^, *Δ*SBS-GRE deletions were generated by replacing the appropriate sequence (key resources table) with a BamHI site. Briefly, two PCR products, containing NheI/BamHI 5′ fragment and BamHI/HindIII 3′ fragment, were cloned into pcDNA3.1(−)-β^ΔUTR^ by three-way ligation. Each sequence was transferred from pcDNA to pBabe retroviral vector using EcoRI and SalI sites.

#### Retroviral and lentiviral infections

GP2 packaging cells (for retroviral plasmids) or HEK 293T cells (for lentiviral plasmids) were seeded at 3 × 10^6^ cells per 100 mm culture dish for 24 h before transfection with 20 μg of the viral expression plasmid containing the insert of interest. Plasmids were co-transfected by calcium phosphate precipitation together with the following packaging and/or envelope plasmids: 4 μg of pVSVG (for retroviral plasmids) or 6 μg of pMD2.G, 10 μg of pMDL g/p RRE, 5 μg of pRSV-Rev (for lentiviral plasmids). Supernatants containing infectious viral particles were harvested at 24, 36 and 48 h post-transfection and filtered through 0.45 μm PVDF filters. Cells were cultured in 100 mm culture dishes for 24 h before infection with viral supernatants in the presence of 8 μg/mL polybrene.

#### Transient transfection

HEK 293T cells were transfected with 100 ng of pcDNA 3.1(−)-HRAS^G12V^ and 1 μg of pcDNA 3.1(−) empty vector or C/EBPβ deletion mutant constructs in 100 mm dishes using X-tremeGENE HP (Roche) according to manufacturer’s instructions. After transfection, cells were cultured for 48 h before harvesting.

#### Protein lysates

For cytoplasmic and nuclear fractionation, cells were rinsed twice with ice-cold PBS, resuspended in hypotonic Buffer A (20 mM HEPES pH 7.9, 1 mM EDTA, 10 mM NaCl, 1 mM DTT, 0.1% NP-40 substitute, 1X protease and phosphatase inhibitor sets) and incubated on ice for 10 min. Nuclei were pelleted by centrifugation for 10 min at 1,200×*g* at 4 °C. Supernatants (cytoplasmic extract) were frozen at −80°C. Nuclear pellets were rinsed with Buffer A (without NP-40) to remove residual cytoplasmic contaminants. Nuclear proteins were extracted by incubation of nuclei in hypertonic Buffer C (25 mM HEPES [pH 7.9], 0.2 mM EDTA, 700 mM NaCl, 0.2 mM DTT, 25% glycerol, 1X protease and phosphatase inhibitor sets) for 30 min at 1,300 rpm at 4 °C. Nuclear debris was pelleted by centrifugation for 10 min at 12,000×*g* at 4 °C, and the supernatant (nuclear extract) was stored at −80°C. Chromatin fractionation was performed as described with minor modifications.^70^^,^^71^ Briefly, cells were rinsed twice with ice-cold PBS, resuspended in Buffer A∗ (10 mM HEPES [pH 7.9], 10 mM KCl, 1.5 mM MgCl_2_, 340 mM sucrose, 10% glycerol, 0.1% NP-40, 1 mM DTT, 1X protease and phosphatase inhibitor sets) and incubated on ice for 10 min. Nuclei were pelleted by centrifugation for 5 min at 1,200×*g* at 4 °C. Obtained supernatant was labeled as cytoplasmic extract and frozen at −80°C. Nuclear pellets were rinsed with Buffer A∗ (without NP-40) to remove residual cytoplasmic contaminants. Nuclear pellet was incubated with Buffer B (3 mM EDTA, 0.2 mM EGTA, 1 mM DTT, 1X protease and phosphatase inhibitor sets) for 30 min at 1,300 rpm (thermomixer) at 4 °C. Insoluble chromatin fraction was collected by centrifugation for 4 min at 1,300×*g* at 4 °C, and the supernatant was designated as nucleoplasmic extract and stored at −80°C. Insoluble chromatin fraction was gently rinsed three times with ice-cold Buffer B and collected by centrifugation for 4 min at 1,300×*g* at 4 °C. Chromatin-bound proteins were obtained by incubation of insoluble chromatin fraction with DNase I solution (0.1 U/μL DNase I, 10 mM Tris-HCl pH 7.5, 2.5 mM MgCl_2_, 0.1 mM CaCl_2_, 1X protease and phosphatase inhibitor sets) at 37 °C for 1 h. Soluble chromatin fraction was cleared by centrifugation at 16,000×*g*, 10 min, 4 °C and supernatant was frozen at −80°C. For whole cell lysates, cells were rinsed twice with ice-cold PBS, harvested in RIPA buffer (10 mM Tris pH 7.4, 150 mM NaCl, 1 mM EDTA, 0.5% sodium deoxycholate, 0.1% SDS, 1% Triton X-, 1X protease and phosphatase inhibitors sets), kept on ice for 10 min, and centrifuged for 10 min at 12,000×*g* at 4 °C. Supernatant was stored at −80°C. Protein concentrations were determined by Bradford protein assay (Bio-Rad).

#### RNA affinity purification of GRE interacting proteins

The RNA pull-down method was modified from Butter et al.^26^ The GRE and GFP sequences were fused to the S1 aptamer tag, which has high affinity for streptavidin.^72^ The GRE and GFP templates for *in vitro* transcription (TranscriptAid T7 High Yield Transcription Kit) were purified PCR products made with oligonucleotides containing the T7 RNA polymerase promoter sequence at the 5′ end and the aptameric tag at the 3′ end (key resources table). The RNA was purified using standard phenol/chloroform protocols and stored in RNase-free water at −80°C. The S1-tagged RNA was refolded by heating for 3 min at 95 °C, cooling on ice for 2 min, adding the RNA buffer (10 mM Tris-HCl [pH 8.0], 150 mM NaCl, 1 mM MgCl_2_, 0.1 mM EDTA) and incubating for 20 min at 20 °C. The RNA was bound to RNase-free Dynabeads MyOne Streptavidin C1 in RNA binding buffer (RBB: 50 mM HEPES·[pH 7.4], 150 mM NaCl, 0.5% NP-40 substitute, 10 mM MgCl_2_) by incubation with gentle rotation for 30 min at 4 °C, followed by three washes with RBB. Beads were incubated with 400 μg NIH 3T3 cytoplasmic extract (in Buffer A with NaCl concentration adjusted to 150 mM and prepared with RNase inhibitor), 40 U of RNase inhibitor, and 20 μg of yeast tRNA for 30 min at 4 °C (final volume 100 μL). After three RBB washes with gentle rotation for 10 min each at 4 °C, the RNA was competed from the beads with elution buffer (16 mM biotin, 50 mM HEPES [pH 7.4], 250 mM NaCl, 0.5% NP-40 substitute, 10 mM MgCl_2_). Eluates were frozen at −80°C until used for immunoblotting or MS analysis.

#### Mass spectrometry (LC-MS/MS) to identify GRE interacting proteins

Coomassie stained gel bands were chopped to small pieces and destained using 50% CAN, 25 mM NH_4_HCO_3_ [pH 8.4]. After removal of organic solvent, the gel pieces were vacuum dried for 45 min. The dried gel pieces were rehydrated in 50 μL of trypsin (20 ng/μL) resuspended in 25 mM NH_4_HCO_3_ and incubated on ice for 30 min. Excess trypsin was removed and replaced with 30 μL of 25 mM NH_4_HCO_3_ [pH 8.4]. The samples were incubated at 37 °C for 16–18 h. Peptides were extracted from the gel bands with 70% (v/v) CAN, 0.1% TFA. The extracted peptides were lyophilized, desalted using C18 columns and reconstituted in 0.1% TFA. The peptides were analyzed using mass spectrometry as described below. The samples were subjected to nanoflow liquid chromatography on a 25 cm Acclaim PepMap C18 column (Easy nLC 1200) coupled to high resolution tandem mass spectrometer (Q Exactive HF). MS precursor scans were performed at a resolution of 60,000 with an ion accumulation target set at 1e^6^, maximum IT at 120 ms, over a mass range of 380–1580 m/z. MS2 analysis was performed at a resolution of 15,000 with an ion accumulation target set at 2e^5^, maximum injection time at 50 ms, at an isolation with of 1.4 m/z over a scan range of 200–2000 m/z. Top 20 precursor ions were selected for fragmentation at a normalized collision energy of 27, charge state 1 and >8 were excluded with a 25 s dynamic exclusion. Acquired MS/MS spectra were searched against the mouse Uniprot protein database using SEQUEST and the Percolator validator algorithms in Proteome Discoverer 2.2. The precursor ion tolerance was set at 10 ppm and the fragment ions tolerance at 0.02 Da. Maximum 2 missed cleavage side were allowed, a minimum peptide length was set at 6 amino acids with methionine oxidation as dynamic modification. A false discovery rate of 0.01 was used for the decoy database search for peptide identification.

##### MS data analysis

The MS results identified 25,188 PSMs bound to GRE representing 1107 proteins, and 13,352 PSMs bound to GFP representing 763 proteins (the sum of PSM counts from three independent affinity purification experiments was used). Overall, there were 4616 identified proteins that bind to either GPF or GRE. Since the GRE and GFP pull down experiments were performed independently, the null hypothesis is that there should be no preference for binding to one or the other. By determining the number of PSMs from a protein that bound to GRE and the total number that were bound to either GRE or GFP, a Binomial test was used to determine if the number of GRE-bound PSMs is significantly different from that expected by chance. Using a threshold *p*-value of 0.05, all proteins whose ratio of GRE-bound PSMs to GFP-bound PSMs was at least 2.0 were selected. In cases where there were no GFP-bound PSMs, this was increased to 1 to produce a valid ratio. The 324 proteins that show a significant preference for binding to GRE were submitted to DAVID^27^ for functional analyses.

#### Immunoblotting

Equal amounts of total proteins were separated on 4–15% SDS-PAGE and blotted to PVDF membranes. Membranes were blocked for 1 h in TBS-T buffer (20 mM Tris [pH 7.6], 150 mM NaCl, 0.1% Tween 20) containing 5% nonfat milk and were then incubated with primary antibodies for 16–18 h at 4 °C. Membranes were rinsed three times in TBS-T, incubated for 1 h at 20 °C with the appropriate secondary antibodies in 5% nonfat milk (in TBS-T), then rinsed three times in TBS-T before imaging using chemiluminescent ECL substrate.

#### Immunoprecipitation (IP)

400 μg of cytoplasmic extract (where noted, with 200 μg/mL RNase A treatment for 10 min at 20 °C) or 200–500 μg nuclear proteins were incubated with primary antibodies with gentle rotation for 3 h at 4 °C in IP buffer (20 mM Tris pH 8.0, 137 mM NaCl, 10% glycerol and 1% NP-40 substitute, 1X protease and phosphatase inhibitor sets). Dynabeads Protein G were added to the samples and incubated with gentle rotation for 1 h at 4 °C. Immunocomplexes were washed four times in IP wash buffer (20 mM Tris pH 8.1; 137 mM NaCl; 0.5% NP-40; 1 mM EDTA) and eluted in 2X Laemmli sample buffer with β-mercaptoethanol for 10 min at 95 °C. Eluted samples were analyzed by immunoblotting.

To detect phospho-C/EBPβ, 500 μg nuclear lysate was incubated with *p*-Ser222 C/EBPβ antibody^18^ for 3 h at 4 °C with gentle rotation in IP buffer (20 mM Tris [pH 8.0], 137 mM NaCl, 10% glycerol and 1% NP-40 substitute, 1X protease and phosphatase sets). Dynabeads Protein G were added to the samples and incubated with gentle rotation for 1 h at 4 °C. Immunocomplexes were washed four times in IP wash buffer (20 mM Tris [pH 8.1]; 137 mM NaCl; 0.5% NP-40; 1 mM EDTA) and eluted in 2X Laemmli sample buffer with β-mercaptoethanol for 10 min at 95 °C. Eluted samples were analyzed by immunoblotting using a total C/EBPβ mouse antibody.

#### Native RNA immunoprecipitation (nRIP)

400 μg of cytoplasmic extracts (in Buffer A with 150 mM NaCl, 1 mM MgCl_2_ and prepared with 400 U/mL RNase inhibitor) were incubated with primary antibodies with gentle rotation for 3 h at 4 °C in NT-2 buffer (50 mM Tris [pH 7.4], 150 mM NaCl, 1 mM MgCl_2_, 0.05% NP-40 substitute, 400 U/mL RNase inhibitor, 1X protease and phosphatase sets). Dynabeads Protein G were added to the samples and incubated with gentle rotation for 1 h at 4 °C. Immunocomplexes were washed four times in NT-2 buffer, processed for RNA extraction/purification, and analyzed by RT-qPCR with sequence-specific primers. For confirmation of immunoprecipitated proteins, the same protocol was performed in parallel except that after washes, beads were eluted in 2X Laemmli sample buffer with β-mercaptoethanol for 10 min at 95 °C. Eluted samples were analyzed by immunoblotting.

#### Electrophoretic mobility shift assay (EMSA)

Nuclear extracts were incubated with double-stranded oligonucleotide probe containing a consensus C/EBP binding site sequence labeled with [γ-^32^P]ATP and complexes separated by native PAGE, as previously described.^20^

#### Quantitative real-time PCR

Total RNA was extracted and purified using QIAshredder and GeneJet RNA purification kit and reverse transcribed using Maxima first strand cDNA synthesis kit for RT-qPCR with dsDNase according to the manufacturers’ protocols. Relative gene expression was measured by quantitative PCR using SsoAdvanced SYBR Green Supermix and mouse or human gene-specific primers. *Ppia/PPIA* or *B2m/B2M* were used as normalization reference genes.

#### RNA FISH and immunofluorescence (IF) staining

For RNA FISH, Quanti-Gene ViewRNA ISH Cell Assay was used according to the manufacturer’s protocol with the following modifications. Cells were plated at 1–2 × 10^4^ cells per chamber on μ-slides VI0.4 (Ibidi). After 1–2 days, cells were rinsed in ice-cold Cytoskeleton Buffer (CB; 10 mM MES pH 6.1, 150 mM NaCl, 5 mM MgCl_2_, 5 mM EGTA, and 5 mM glucose),^73^ permeabilized and fixed in ice-cold pre-fixative mix in CB (4% paraformaldehyde, 0.01% glutaraldehyde, 0.05% saponin)^74^ for 10 min at 4 °C. Cells were then incubated with ice-cold fixative mix in CB (4% paraformaldehyde, 0.01% glutaraldehyde) for 2 h at 4 °C. To better preserve localization of mRNA and proteins, cells were treated with SHIELD mix (SHIELD epoxy/SHIELD OFF buffer, 3:1 v/v) for 30 min at 37 °C.^75^ Cells were rinsed with CB twice and quenched with 50 mM NH_4_Cl (in CB) for 5 min at 20 °C. After FISH hybridization steps, cells were rinsed in PBS-S (0.05% Saponin, in PBS) and blocked with 5% albumin in PBS-S for 1 h at 20 °C. Cells were then incubated with the indicated primary antibodies for 16–18 h at 4 °C in 5% albumin in PBS-S. Cells were rinsed three times for 5 min at 20 °C with PBS-S and incubated for 1 h at 20 °C with AlexaFluor-conjugated secondary antibodies in 5% albumin in PBS-S. Cells were rinsed twice with PBS-S, co-stained with 0.1 mg/mL DAPI (in PBS) for 2 min at 20 °C, and then rinsed three times with PBS. Slides were kept in PBS at 4 °C for 1–3 days until imaging. Images were acquired using either a Zeiss LSM 780 or 880 confocal microscopes or a Zeiss LSM 880 AiryScan super-resolution microscope. Images were processed and analyzed using Zen black and Zen blue software.

#### Live cell imaging

Cells were transduced with supernatants of lentiviral constructs expressing fluorescently tagged proteins. After selection with the appropriate marker, cells were plated at 2 × 10^4^ cells per chamber on μ-slides VI0.4. The cells were observed by confocal microscopy the following day.

#### Image analysis

##### Relative nuclear proximity index (RNPI)

RNPI is an inhouse ImageJ-based tool that measures the average distance of cytoplasmic signals (in this case *CEBPB* mRNA labeled by RNA FISH) to the nuclear boundary per cell, normalized to a uniform distribution of the same signals. Details of the method have been described.^36^

##### mRNA distribution in cell areas

To quantify mRNA distribution in different areas of the cell, Imaris software version 9.8 was used to fit mRNA signals to the spots module and protein signals to the surface module. The identified surfaces and spots were combined in the cells module to further elucidate the specific numbers of mRNA spots within a defined region of the cell. One spots module, three surface modules, and two cell modules were used to conduct the analysis. The mRNA signals were defined setting a defined size for the spot of 0.400 μm in XY and using a manual quality threshold value according to visual inspection of the image. There were multiple surfaces defined nuclei, kinase region, and cell boundaries. Nuclei were defined using the surface module and DAPI staining to identify individual nuclei. The protein signal from immunofluorescent staining of the CK2 kinase was used to define the kinase region using a manual threshold of high intensity signals and smoothing to find a defined region for CK2. The cell boundaries were determined by both oversaturating the kinase signal and using a DIC image of the cells. Two cell modules were built. Cell module one identified the cell boundary, nuclei, and mRNA spots. Cell module one was used to find mRNA spots in the cytoplasm and total mRNA spots within the entire cell. Cell module two identified the kinase region, nuclei, and mRNA spots. Cell module 2 was used to define mRNA spots in the kinase region. Number of *CEBPB* mRNA molecules per cell and % of *CEBPB* mRNA overlapping the kinase region per cell were calculated.

##### Nearest neighbor analysis

DiAna is an ImageJ plugin used to measure distances between objects of two different species, such as objects with distinct fluorescence labels in optical microscope images.^66^ In this application, DiAna is used to measure in the cytoplasm the shortest distance from one punctate species to another, in this case *CEBPB* transcripts labeled by RNA-FISH and CK2α foci labeled by immunofluorescence. Prior to executing DiAna, punctate signals in each channel were detected and the borders of the cytoplasm and nuclei for each cell were drawn. Punctate signals were detected by first choosing a minimum threshold intensity for puncta in the image. Then the puncta were detected by the 3D Maxima Finder plugin that is part of the 3D Suite in ImageJ. The settings for the Finder were the minimum threshold, an XY radius of 5, a Z radius of 1 and the noise level set to the square root of the minimum threshold. Only detected puncta inside the cytoplasm border and outside the nucleus border were used. Each cell in an image was analyzed separately. DiAna was implemented using the “Center-to-Center” and “Distance” options to measure the Euclidean distance from the centers of puncta of one species to the nearest center of puncta of the other species. The first punctate species was selected by having fewer puncta than the other punctate species to minimize the number of measurements. Stepwise instructions and an ImageJ macro for executing the method will be provided upon request.

#### mRNA decay assay

mRNA decay rates were determined using 5-ethynyluridine (EU) labeled mRNA chase assay. A549 cells were plated at 10^5^ cells/well in 6-well dishes. After 24 h, 0.1 mM EU was added to the media for 16 h. After incubation, cells were either harvested (time 0) or media was replaced without EU and the cells were cultured for the indicated time points before RNA harvesting to allow degradation of the labeled RNA (chase). The isolated EU-labeled RNA was used with the Click-iT Nascent RNA Capture Kit according to the manufacturer’s instructions.

#### Proximity ligation assay (PLA)

A549 cells were plated at 4 × 10^4^ cells per chamber on μ-slides VI0.4. The next day, cells were rinsed in ice-cold PBS and fixed with methanol for 15 min at −20°C, permeabilized with 0.05% Saponin in PBS for 5 min at 20 °C and rinsed three times with PBS. After fixation, Duolink *in situ* detection system was used according to the manufacturer’s instructions (volumes were adjusted accordingly for μ-slides). Images were acquired using a Zeiss LSM 780 confocal microscope.

#### Colony forming assays

Cells were plated at 2000 cells per 100 mm culture dish. After 10–14 days in culture, colonies were fixed in 4% formaldehyde in PBS for 10 min, stained with 0.1% crystal violet for 30 min and counted.

#### Focus formation assays

Cells were plated at 1000 cells per 100 mm culture dish together with 1.5 × 10^5^ nontransformed NIH 3T3 or *p19*^*Arf−/−*^ MEFs (lawn). After 10–14 days, cells were fixed in 4% formaldehyde in PBS for 10 min, stained with 0.1% crystal violet for 30 min and foci were counted.

#### Senescence-associated β-galactosidase (SA-βGal) assays for cultured cells

Cells were plated at 2.5 × 10^4^ cells per well in 6-well plates. After 2–3 days, cells were stained using a senescence detection kit with minor modifications of the manufacturer’s instructions. For mouse cell lines, the pH of the staining solution mix was lowered by adding 1.54 μL of 2 M HCl per mL.

#### Bulk RNA sequencing

Five independent, low passage cultures for *WT*, *GRE*^*Δ/Δ*^, *p19*^*Arf−/−*^, and *p19*^*Arf−/−*^;*GRE*^*Δ/Δ*^ MEFs were transduced with HRAS^G12V^ or control lentiviruses and selected with hygromycin for four days. Eight days later RNA was prepared and analyzed by RNA-seq.

##### Library preparation and sequencing

The Illumina stranded Total RNA Prep and ligation with ribo zero Plus kit was used for library prep. This protocol involves the removal of ribosomal RNA (rRNA) using biotinylated, target-specific oligos combined with Ribo-Zero rRNA removal beads. The RNA is fragmented into small pieces and the cleaved RNA fragments are copied into first strand cDNA using reverse transcriptase and random primers, followed by second strand cDNA synthesis using DNA Polymerase I and RNase H. The resulting double-strand cDNA is used as the input to a standard Illumina library prep with end-repair, adapter ligation and PCR amplification. The final purified product is then quantitated by qPCR. The sequencing run was set up using paired end (2 × 100 cycle) sequencing on NovaSeq S1 flowcell.

##### Data preprocessing and analysis

The Illumina bcl2fastq2.20 was used to demultiplex and convert binary base calls and quality scores to fastq format. The sequencing reads were trimmed of adapters and low-quality bases using Cutadapt (version 1.18). The trimmed reads were mapped to mouse reference genome (mm10) and Gencode annotation M21 using STAR aligner (version 2.7.0f) with two-pass alignment option. RSEM (version 1.3.1) was used for gene and transcript quantification based on GENCODE annotation file.

##### Analysis of differentially expressed genes

Differentially-expressed genes (DEGs) from the RNAseq data were identified using the Bioconductor package DESeq2 to evaluate robustness and consistency of results (we report results from DESeq2, although similar results were observed using the edgeR method). Paired samples were used to set up the models for differential analysis. Built-in utility functions such as plotPCA as well as customized plot functions were used to assess overall behaviors of the data. For DESeq2 we followed all default settings and lfcShrink function was used for Log fold change shrinkage of the DEGs lists. DEGs were extracted using cutoffs at log2(fold change) > 0.58 (fold change above 1.5) and adjusted *p*-value < 0.05 (DESeq2) or FDR < 0.05 (edgeR). The corresponding DEG gene lists were collected and applied to customized R scripts that implemented the in-house pathway pattern analysis method, PPEP.^67^ This analysis was modified by supplementing the annotated Gene Ontology databases with extra gene sets derived from published senescence gene signatures (Casella et al.,^76^ Xu et al.^77^ Saul et al.^78^ Hernandez-Segura et al.,^79^ and Freund et al.^80^) and from CellAge.^81^ Briefly, gene lists were subjected to Fisher’s exact test-based pathway or gene set enrichment analysis using annotated Gene Ontology databases supplemented with the published senescence gene sets mentioned above. The derived *p*-values were transformed by using the formula (−1)∗log10(*p*-value). All *p*-values less than 0.05 and *p*-values with the number of “hit” genes from the gene list for the corresponding pathway/gene set less than 2 were converted to 0. The resulting transformed *p*-value data matrix was used to derive pathway-level heatmaps using either customized R scripts that used the pheatmap or gplots R package. The “hit” genes for specific pathways or gene sets from selected DEG lists were retrieved using PPEP-based customized R scripts, and gene-level heatmaps were generated similarly to the pathway-level heatmaps.

#### S100a9 depletion and overexpression in MEFs

S100a9 depletion using antisense oligonucleotides (ASOs) was performed in HRAS^G12V^-expressing *p19*^*Arf−/−*^;*GRE*^*Δ/Δ*^ MEFs, as follows. MEFs were first transduced with CMV51p-HRAS^G12V^ lentivirus and selected for 5 days with 100 μg/mL hygromycin. After selection, on day 1, 1 × 10^6^ MEFs were plated in 10 cm dishes and 5 × 10^4^ MEFs in 6 well plates to achieve 30–50% confluence at the time of transfection. Transfection of S100a9 ASOs was performed on day 2. Two tubes were prepared for 10 cm dishes, one containing 1 mL of Opti-MEM media and 20 μL of RNAiMAX reagent and another containing 1 mL of Opti-MEM media and 20 μL of 3 nM ASO solution. Two other tubes were prepared (for 6 well plates), one containing 50 μL of Opti-MEM media and 3 μL of RNAiMAX reagent and another containing 50 μL of Opti-MEM media and 2 μL of 3 nM ASO solution. The tubes were incubated at room temperature for 5 min without vortexing, after which the contents were mixed and gently vortexed followed by a 15 min incubation at room temperature. Meanwhile, the media from MEFs cultures was removed, a single PBS wash was performed and 8 mL of media was added to 10 cm dishes and 900 μL to 6 well plates. The ASO-RNAiMAX mixtures were added to the plates, which were placed in a CO_2_ incubator for 6–7 h. The media was removed and fresh media was added. The ASO transfection was repeated on day 4. On day 6, the cells were re-plated to analyze gene knockdown (10 cm dishes) and senescence (SA-βGal staining; 6 well plates). Relative *S100a9* gene expression was measured by quantitative PCR using SsoAdvanced SYBR Green Supermix and mouse gene-specific primers, with *Ppia* as a reference gene.

S100a9 overexpression experiments were performed in *p19*^*Arf−/−*^ MEFs ± HRAS^G12V^. Cells were transduced with CMV51p-HRAS^G12V^ or control CMV13p lentiviruses and selected with hygromycin for 5 days. After selection, stable MEF cultures were established and subsequently transduced with lentiviral vectors containing *S100a9* ORF or eGFP (control) and selected with 2 μg/mL puromycin for 2 days. After selection, whole cell lysates were prepared for immunoblot analysis. Some cells were further cultured for 5-6 days to analyze senescence (SA-βGal staining).

##### S100A9 Luminex assay

Cell lysates were diluted to an equivalent concentration of total protein, then analyzed for levels of S100A9 using the Luminex Discovery Assay kit. Briefly, lysates were added to a 96-well plate and incubated with magnetic microparticles pre-coated with antibodies to murine S100A9. A magnetic plate was then used to immobilize the microparticles and wash away any unbound material. A biotinylated antibody to S100A9 was added, followed by a streptavidin-phycoerythrin (PE) conjugate. Following a final wash, the microparticles were resuspended in buffer and PE fluorescence levels were measured using an FLEXMAP 3D reader. The data was analyzed using Bio-Plex Manager software, calculating the S100A9 levels in each cell lysate sample based on the S100A9 standards.

#### Histological examination of lung tumor tissue

In the *Kras*^*LA2*^ model, expanding and replacing pulmonary parenchyma are multifocal, variably-sized, up to 7 mm diameter, well-demarcated, unencapsulated neoplastic foci composed of cuboidal to columnar epithelial cells arranged in either packets, solid sheets, and glands or forming papillary structures, supported by a fine fibrovascular stroma. Neoplastic cells in adenomatous nodules have variably-distinct cell borders, moderate amounts of eosinophilic cytoplasm, and round to oval nuclei with finely-stippled chromatin, and indistinct nucleoli. The mitotic rate is less than 1 per 20 high-power field (HPF). Multifocally, within one or more nodules, neoplastic cells forming adenocarcinomas display anisocytosis, which arranges in disorganized glands or solid sheets. Neoplastic cells have either scant to moderate amount of eosinophilic to amphophilic cytoplasm or have clear cytoplasm. Nuclei are moderately pleomorphic with fine to coarse chromatin and have prominent nucleoli. Mitotic rate is 0–2 per 20 HPF. Within the adjacent parenchyma, multifocally alveolar spaces often contain alveolar macrophages.

#### Image analysis of tissue samples

All image analysis was performed using HALO imaging analysis software and image annotations were performed in a blinded fashion. Lung sections were annotated with HALO manual annotation tools. Nonspecific regions such as esophagus, thymus, and trachea and areas of artifact such as folds and tears were excluded from analysis. The HALO random forest tissue classifier was used to automatically differentiate neoplastic cells from normal lung parenchyma to determine total tumor burdens. Ki67 image analysis was performed using cytonuclear algorithm in HALO version 3.3 to determine percentage of positive cells. HALO MiniNet AI tissue classifier was used to automatically differentiate tumor cells from normal lung parenchyma and to differentiate benign adenomas form malignant adenocarcinomas. The size of nuclei was measured from Ki67 IHC stained slides. Multiple nodules (adenomas and adenocarcinomas) were selected in two different layers. HALO Nuclei Seg (plugin) classifier was used to segment nuclei.

#### SA-βGal staining of tumor-bearing lung sections

Mice were euthanized following the approved ACUC protocol. Lung lobes were dissected and individually frozen in OCT on dry ice. Frozen sections were cut at 10 microns and stored at −80°C until staining. Immediately prior to staining, slides were fixed in 1% formaldehyde/0.2% glutaraldehyde/0.2% Igepal for 10 min. Slides were then rinsed and washed in PBS for 10 min. Several drops of β-gal staining solution (1 mg/mL Xgal, 40 mM citric acid/Na phosphate buffer pH 5.8, 5 mM K_3_[Fe(CN)_6_], 5 mM K_4_Fe(CN)_6_, 150 mM NaCl, 2 mM MgCl_2_) were placed on the slides. The slides were then placed in a dark water bath at 37 °C overnight. After staining, slides were rinsed and washed in PBS for 10 min, followed by two 5 min washes in distilled water. Slides were counterstained with a 0.1% Neutral Red solution for 0.5 to 2 min. Finally, slides were dehydrated in 100% ethanol, cleared through four changes of xylene, mounted with Permount, and cover-slipped. All slides were digitally scanned at 20× magnification utilizing an Aperio AT2 whole-slide scanner. Lung nodules were annotated, and the percentage of positive cells was quantified using the HALO software, employing a cytonuclear algorithm and area quantification. Areas of artifact and background staining, such as those found in papillary nodules, were excluded from the analysis.

#### Immunohistochemical (IHC) staining of tissue sections

For Ki67 IHC staining, slides generated from paraffin blocks (5 μm sections) were dewaxed using xylene and then hydrated using a series of graded ethyl alcohols. Slides were stained with Hematoxylin and Eosin-Y (H&E) following standard protocols using the Sakura Tissue-Tek Prisma automated stainer. A regressive staining method was used. This method intentionally overstains tissues and then uses a differentiation step (Clarifier/Bluing reagents) to remove excess stain. After staining was completed, the slides were coverslipped using the Sakura Tissue-TekGlass automatic coverslipper, dried and were ready for review. IHC staining was performed on LeicaBiosystems’ BondRX autostainer with the following conditions: Epitope Retrieval 1 (Citrate) for 20 min, Ki67 antibody (1:200 for 30 min), and the Bond Polymer Refine Detection Kit, with omission of the PostPrimary Reagent. Rabbit monoclonal IgG XP Isotype Control was used in place of Ki67 for the negative control. Slides were removed from the Bond autostainer, dehydrated through ethyl alcohols, cleared with xylenes, and coverslipped. H&E and IHC slides were scanned at 20x using an Aperio AT2 scanner into whole slide digital images.

For S100a9, CD206 and CD86 staining, FFPE blocks were sectioned at 5 microns in preparation for multiplex IHC. Staining was performed using a Leica Bond RX autostainer. Initial antigen retrieval was performed using EDTA buffer for 20 min at 100 °C on the Bond autostainer. The primary antibody for S100a9 was diluted 1:500, with a 30-min incubation time. S100a9 antibody detection was accomplished using the Bond Polymer Refine Detection kit with the Post-Primary reagent, DAB, and Hematoxylin removed from Leica’s default staining protocol. Fluorescent Opal570 reporter dye was added to the tissue per the manufacturer’s instructions.

Following application of the Opal570 reagent, EDTA antigen retrieval solution was applied again to the tissues for 20 min at 95 °C. CD206 antibody was applied at a 1:400 dilution for 30 min. CD206 antibody detection was accomplished using the Bond Polymer Refine Detection kit with the Post-Primary reagent, DAB, and Hematoxylin removed from Leica’s default staining protocol. Fluorescent Opal520 reporter dye was added to the tissue per the manufacturer’s instructions. After application of the Opal520 reagent, EDTA antigen retrieval solution was applied again to the tissues for 20 min at 95 °C. CD86 antibody was applied at a 1:50 dilution for 60 min. CD86 antibody detection was accomplished using the Bond Polymer Refine Detection kit with the Post-Primary reagent, DAB, and Hematoxylin removed from Leica’s default staining protocol. Fluorescent Opal690 reporter dye was added to the tissue per the manufacturer’s instructions. All slides were digitally scanned at 20x using an Aperio FL whole-slide scanner. Normal mouse spleen was used as a positive control for all stains. The scanned slides were analyzed using HALO. Initially, benign and carcinomatous lesions were annotated separately. Subsequently, the percentage of positive cells was quantified using HighPlex FL (v3.2.2) algorithm.

#### Single cell RNA sequencing of lung tumor samples

4 *Kras*^*LA2/+*^;*WT* and 4 *Kras*^*LA2/+*^;*GRE*^*Δ/Δ*^ mice were sacrificed when signs of respiratory distress were observed. Lungs were removed and tumor-bearing sections (∼1 cm^3^) containing mixed populations of lesion sizes were dissected and placed into Petri dishes with 5 mL enzyme cocktail: 4 mg/mL collagenase, 1 mg/mL hyaluronidase, 1,000 U/mL benzonase, and 10 mg/mL bovine serum albumin in RPMI medium. Tissue was finely minced using a sterile razor blade and mechanically dissociated by trituration with a 10 mL serological pipette followed by a 5 mL pipette. The resulting cell suspension was centrifuged at 400*g* for 5 min at 4°C and the supernatant discarded. The cell pellet was further dissociated by incubation in 5 mL 3 mg/mL trypsin solution for 7 min at room temperature. The suspension was sequentially filtered through 70 μm and 40 μm cell strainers and rinsed with RPMI containing 10% FBS. Cells were centrifuged at 400*g* for 5 min at 4°C, and red blood cells were lyzed by incubation in 1 mL ACK lysis buffer for 3 min at room temperature. Cells were washed with 5 mL RPMI containing 10% FBS, centrifuged at 400*g* for 5 min at 4°C, and resuspended in 1 mL phosphate-buffered saline (PBS). Cell concentration and viability were determined by Trypan Blue exclusion using a hemocytometer. Cells were pelleted and resuspended in 1 mL cryopreservation medium consisting of 10% dimethyl sulfoxide in FBS, then stored at −80°C until single-cell sequencing was performed.

scRNA-seq libraries were sequenced on an Illumina NovaSeq X Plus instrument. Raw sequencing reads were demultiplexed and aligned to the human reference genome using Cell Ranger. The resulting count matrices were processed using the Seurat package^63^ in RStudio. Low-quality cells were excluded based on the following criteria: cells with fewer than 1,000 or more than 6,000 detected genes, more than 10% mitochondrial gene expression, or more than 30,000 UMI counts were removed. Data were normalized, scaled, and reduced to principal components following Seurat pipeline. Doublets were identified and removed at the sample level using DoubletFinder^62^ with tuned parameters, according to documentation. To correct for batch effects across samples, embeddings were integrated using Harmony.^65^ Corrected embeddings were used to construct a shared nearest-neighbor graph with FindNeighbors, and unsupervised clustering was performed using the Louvain algorithm implemented in FindClusters (using a resolution of 0.5). Cell type annotation was performed using ScType.^69^ Clusters composed exclusively of cells from a single sample were considered sample-specific noise or technical artifacts and were removed from downstream analyses. Aneuploidy was assessed using CopyKAT, which was run on all epithelial cells from cluster 10 and 500 cells from each immune-related cluster. Differentially expressed genes between cell populations were identified using the Wilcoxon rank-sum test implemented in the Presto package, retaining genes expressed in at least 10% of cells within a group.

##### Epithelial subset

Epithelial cells were isolated from the original dataset by retaining cells expressing Epcam, Krt8, and Krt18. Cells expressing immune cell markers (Cd79a, Cd3d, Ptprc) were subsequently removed, with the resulting population showing 100% aneuploidy. Y chromosome genes were removed from the expression matrix to prevent sex-driven transcriptional variation from confounding cell clustering. The resulting epithelial population was re-clustered using the Seurat pipeline. Differential gene expression analysis was performed using the FindAllMarkers function. Gene Set Enrichment Analysis (GSEA) was conducted using the fgsea package (v1.30.0),^64^ with genes ranked by average log2 fold-change and restricted to genes detected in at least 10% of cells. Enrichment analysis for Hallmark pathways was performed using clusterProfiler package (v 4.12.6).^59^ Transcriptional scores for each Marjanovic subcluster signature^46^ were computed using the AddModuleScore function from the Seurat R package.

#### Statistical analysis

Quantitative data are presented as means ± standard deviation with the number of measurements (“n”) indicated in the figure legends, representing biological replicates in cell culture experiments and the number of mice in animal studies. Where appropriate, data were examined for sex-dependent effects. No statistically significant sex-specific differences were detected, and data from male and female animals were pooled. Statistical analysis was performed in GraphPad Prism using paired or unpaired Student’s *t* test, unless otherwise indicated in the figure legends. *p*-values less than 0.05 were considered significant.

## Supplemental information

Document S1. Figures S1–S7

Table S1. Mass spectrometry identification and functional annotation of proteins associated with the *Cebpb* GRE RNA element (A) Table description and abbreviations. (B) Raw peptide-spectrum match (PSM) data by replicate. (C) GRE versus GFP comparisons for all identified proteins. (D) GRE-enriched proteins (GRE:GFP ratio ≥2.0; binomial *p* value <0.05). (E) DAVID functional annotation of GRE-enriched proteins. (F) Raw PSM data from the pilot experiment. (G) GRE-enriched proteins identified in the pilot experiment

Table S2. Published CLIP datasets reporting association of UPF1 with *CEBPB* mRNA

Table S3. Comparison of GRE-associated proteins with published UPF1-interacting proteins (A) Table description. (B) GRE-enriched proteins compared to reported RNA-independent UPF1 binders (Flury et al.). (C) GRE-enriched proteins compared to RNA-dependent UPF1 binders (Flury et al.). (D) All UPF1 interactors reported by Flury et al.

Table S4. Differential gene expression analysis of bulk RNA-seq data from *WT*, *GRE*^*Δ/Δ*^, *p19Arf*^−/−^, and *p19Arf*^−/−^;*GRE*^*Δ/Δ*^ MEFs with or without HRAS^G12V^ expression (A) Table description and abbreviations. (B) All data, *WT* ± HRAS^G12V^. (C) All data, *GRE*^*Δ/Δ*^ ± HRAS^G12V^. (D) All data, *p19*^*Arf−/−*^ ± HRAS^G12V^. (E) All data, *p19*^*Arf−/−*^; *GRE*^*Δ/Δ*^ ± HRAS^G12V^

Table S5. Differentially expressed HRAS^G12V^-responsive genes in *p19Arf*^−/−^ and *p19Arf*^−/−^;*GRE*^*Δ/Δ*^ MEFs (A) Table description and abbreviations. (B) List of HRAS^G12V^-induced DEGs plotted in the Venn diagram of Figure S6D

Table S6. Gene ontology enrichment analysis of senescence-associated transcriptional programs (A) Table description and abbreviations. (B) Enrichment scores for custom GO senescence terms and all other GOBP terms. (C) Enrichment scores for all senescence related pathways

Table S7. Expression of senescence- and SASP-associated genes in HRAS-regulated transcriptomes from *WT*, *p19Arf*^−/−^, and *p19Arf*^−/−^;*GRE*^*Δ/Δ*^ MEFs (A) Table description and abbreviations. (B) Enrichment scores for individual genes across the three genotypes

Table S8. Single-cell RNA-seq analysis of *WT* and *GRE*^*Δ/Δ*^ lung tumors (A) Differentially-expressed genes for each unsupervised cluster. (B) Cluster abundance comparisons (*GRE*^*Δ/Δ*^ vs. *WT*). (C) Immune cell abundance comparisons (*GRE*^*Δ/Δ*^ vs. *WT*)

Table S9. Marker genes defining epithelial subclusters

Table S10. Differentially expressed genes (*GRE*^*Δ/Δ*^ vs. *WT*) within each epithelial subcluster; each tab corresponds to one epithelial subcluster

Table S11. fgsea analysis of differential gene expression in epithelial cells (*GRE*^*Δ/Δ*^ vs. *WT*) (A) Description. (B) Ranked genes list. (C) fgsea results

Table S12. Comparison of epithelial cell transcriptional programs with Marjanovic lung tumor signatures (A) Per cell comparisons for each genotype. (B) Mean scores per genotype

Table S13. Oligonucleotides
