# Supplementary material for: 3′UTR-directed, kinase-proximal mRNA decay inhibits C/EBPβ phosphorylation/activation to suppress senescence in tumor cells
Source: iScience. 2026 Aug 4;29(8):117039. doi: 10.1016/j.isci.2026.117039 (PMC13469816; doi:10.1016/j.isci.2026.117039)

iScience, Volume *29*

Supplemental information

3′UTR-directed, kinase-proximal mRNA decay inhibits C/EBPβ phosphorylation/activation to suppress senescence in tumor cells

Jacqueline Salotti, Nida Asif, Srikanta Basu, Aniruddha Das, Linshan Hu, Mei Yang, Baktiar Karim, Karen Saylor, Nancy Martin, David A. Scheiblin, Sweta Misra, Brian T. Luke, Thorkell Andresson, Ming Yi, Mélissa Galloux, Stephen Lockett, Lino Tessarollo, and Peter F. Johnson

Supplemental Data

Figure S1

A

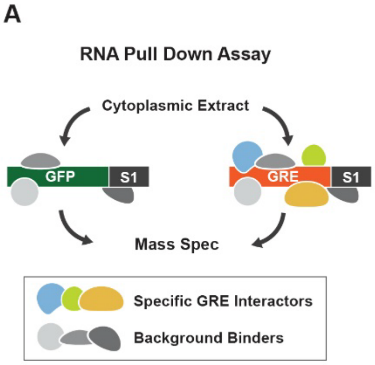

B

SASP mRNA Expression Relative to shCtrl Vector

Knockdown	*Il6*		*Il1a*		*Cxcl1*		*Ccr1*	
	β^{UTR}	shβ	β^{UTR}	shβ	β^{UTR}	shβ	β^{UTR}	shβ
Upf1	300.6	15.4	490.3	11.0	62.9	13.2	21.5	3.8
Strbp	22.0	0.6	108.8	1.0	65.4	3.3	6.7	1.8
Elavl1	6.8	0.6	0.7	0.8	3.2	5.2	29.8	1.4
Ilf3	3.7	2.8	4.3	1.2	0.7	0.5	1.5	0.6
Igf2bp3	3.0	1.2	4.8	0.9	3.4	2.8	0.9	0.2
Fubp3	2.7	0.5	3.6	0.7	4.3	4.2	11.8	2.4
Anxa1	2.3	0.9	1.3	0.5	2.6	1.5	8.9	0.6
Igf2bp1	1.8	0.8	8.1	0.6	1.6	0.6	0.9	0.3
Mapre1	1.6	0.3	2.5	0.0	0.9	0.4	0.8	0.2
Igf2bp2	1.5	0.3	17.4	0.3	1.6	0.7	0.9	0.2
Hnrnpd	1.4	0.7	1.5	0.1	1.0	0.7	0.6	0.3
Dhx9	1.3	0.3	1.4	0.1	1.4	0.8	1.4	0.4
Celf1	1.2	0.5	6.2	1.7	1.7	1.5	7.9	0.9
Fmr1	1.0	0.4	0.7	0.0	0.9	0.6	0.7	0.3
Hnrnpa2b1	0.8	0.5	0.4	0.1	1.2	0.7	1.1	0.4
Anxa2	0.8	1.0	1.1	1.0	1.6	1.0	2.3	1.0
Plec	0.8	0.3	2.3	0.5	1.4	2.2	3.6	0.7
Nono	0.5	0.2	1.1	0.3	1.2	1.2	0.5	0.1
Fubp2	0.5	0.2	1.3	0.6	1.1	3.9	2.3	0.5
Fubp1	0.4	0.3	2.2	1.0	1.6	2.4	4.3	1.3
Ilf2	0.3	0.2	0.5	0.0	0.5	0.1	4.2	1.7

C

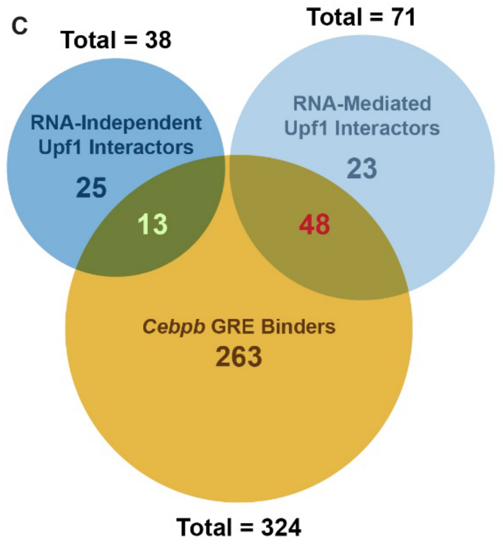

Figure S1. Upf1 interacts with the *Cebpb* GRE to inhibit C/EBPβ-dependent SASP gene expression, Related to Figure 1.

(A) An RNA affinity purification scheme to identify novel GRE-interacting proteins. The procedure is based on the method of Butter *et al.*[1] **(B)** Screening of selected C/EBPβ UPA candidate genes. Each gene was ablated in NIH $3T3^{RAS}$ cells overexpressing $Cebpb^{UTR}$ (βUTR) or depleted for C/EBPβ (shβ), and expression of proinflammatory SASP mRNAs was measured by RT-qPCR. **(C)** Overlap between GRE binding proteins and known RNA-dependent and RNA-independent UPF1 interactors (see Table S3).[2]

Figure S2

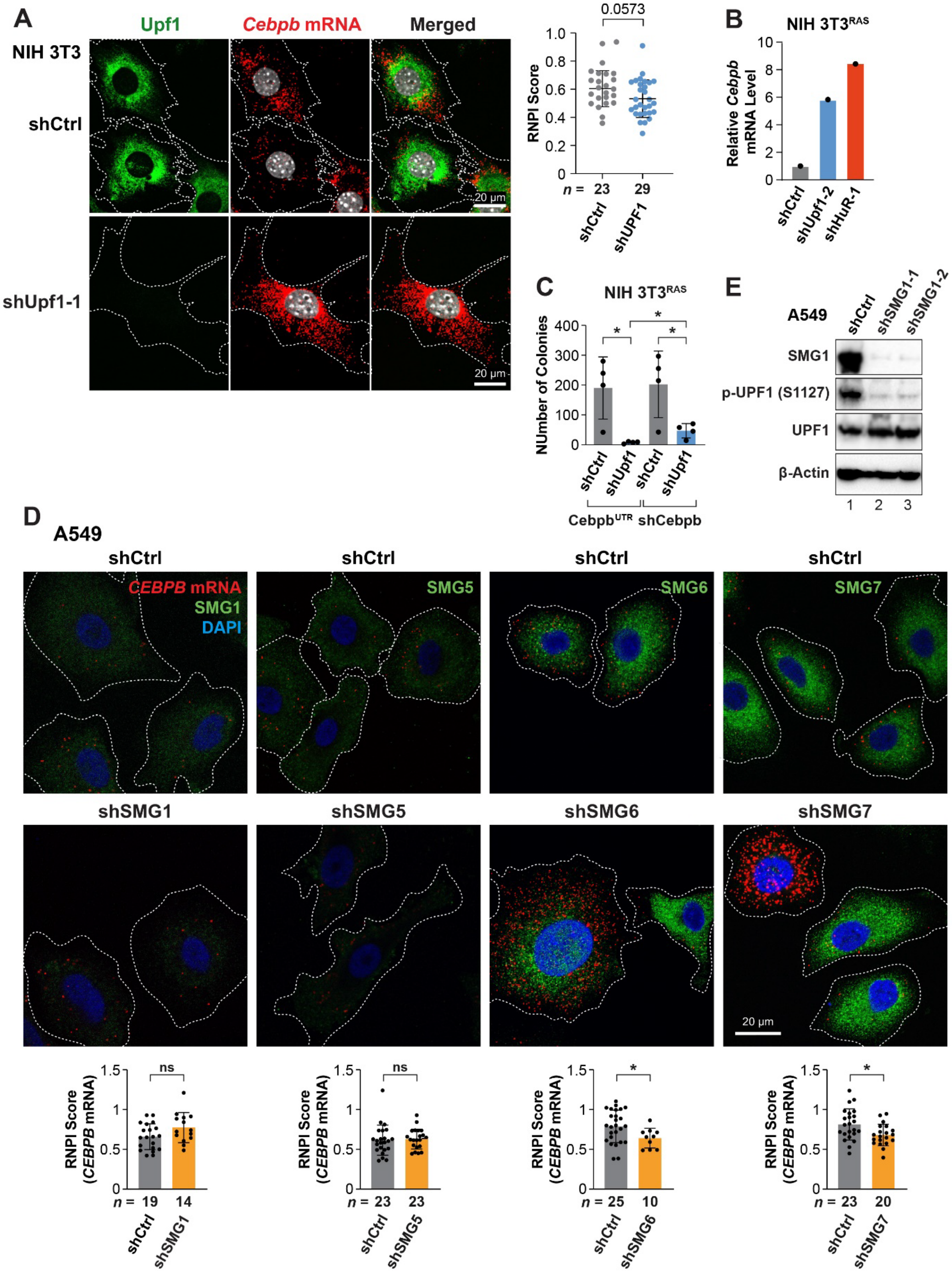
A
Upf1
Cebpb mRNA
Merged
NIH 3T3
shCtrl
shUpf1-1
20 μm
0.0573
RNPI Score
n = 23 29
shCtrl
shUPF1
B
NIH 3T3RAS
Relative Cebpb mRNA Level
shCtrl
shUpf1-2
shHuR-1
C
NIH 3T3RAS
NUmber of Colonies
shCtrl
shUpf1
CebpbUTR
shCebpb
E
A549
shSMG1-1
shSMG1-2
SMG1
p-UPF1 (S1127)
UPF1
β-Actin
1 2 3
D
A549
shCtrl
CEBPB mRNA
SMG1
DAPI
SMG5
SMG6
SMG7
shSMG1
shSMG5
shSMG6
shSMG7
20 μm
RNPI Score (CEBPB mRNA)
ns
*
n = 19 14
n = 23 23
n = 25 10
n = 23 20

Figure S2. Silencing of UPF1, SMG6 and SMG7 increases perinuclear abundance of *CEBPB* transcripts, Related to Figure 2.

(A) Upf1 knockdown in NIH 3T3 cells increases the perinuclear segregation of *Cebpb* transcripts. *Cebpb* mRNA was detected using single molecule RNA FISH together with immunostaining for Upf1. Right: a relative nuclear proximity index (RNPI) score[3] was determined for each cell. n = number of cells analyzed. **(B)** Upf1 depletion increases total *Cebpb* mRNA levels in NIH $3T3^{RAS}$ cells. mRNA levels were quantified by RT-qPCR, normalized to *Ppia*. **(C)** Upf1 depletion in NIH $3T3^{RAS}$ cells impairs clonogenic growth in a manner that is partially C/EBPβ-dependent. **(D)** Silencing of SMG6 or SMG7, but not SMG1 or SMG5, disrupts peripheral segmentation of *CEBPB* transcripts in A549 cells. RNPI scores were determined in control and shSMG images. Only cells with visible reduction of SMG levels were scored in the knockdown groups. n = number of cells analyzed. **(E)** SMG1 depletion reduces UPF1 phosphorylation on Ser1127.

Scale bar dimensions are shown in the micrographs.

Statistical significance was determined using paired (**C**) or unpaired (**D**) Student's t tests. $*p < 0.05$; ns, not significant.

Figure S3

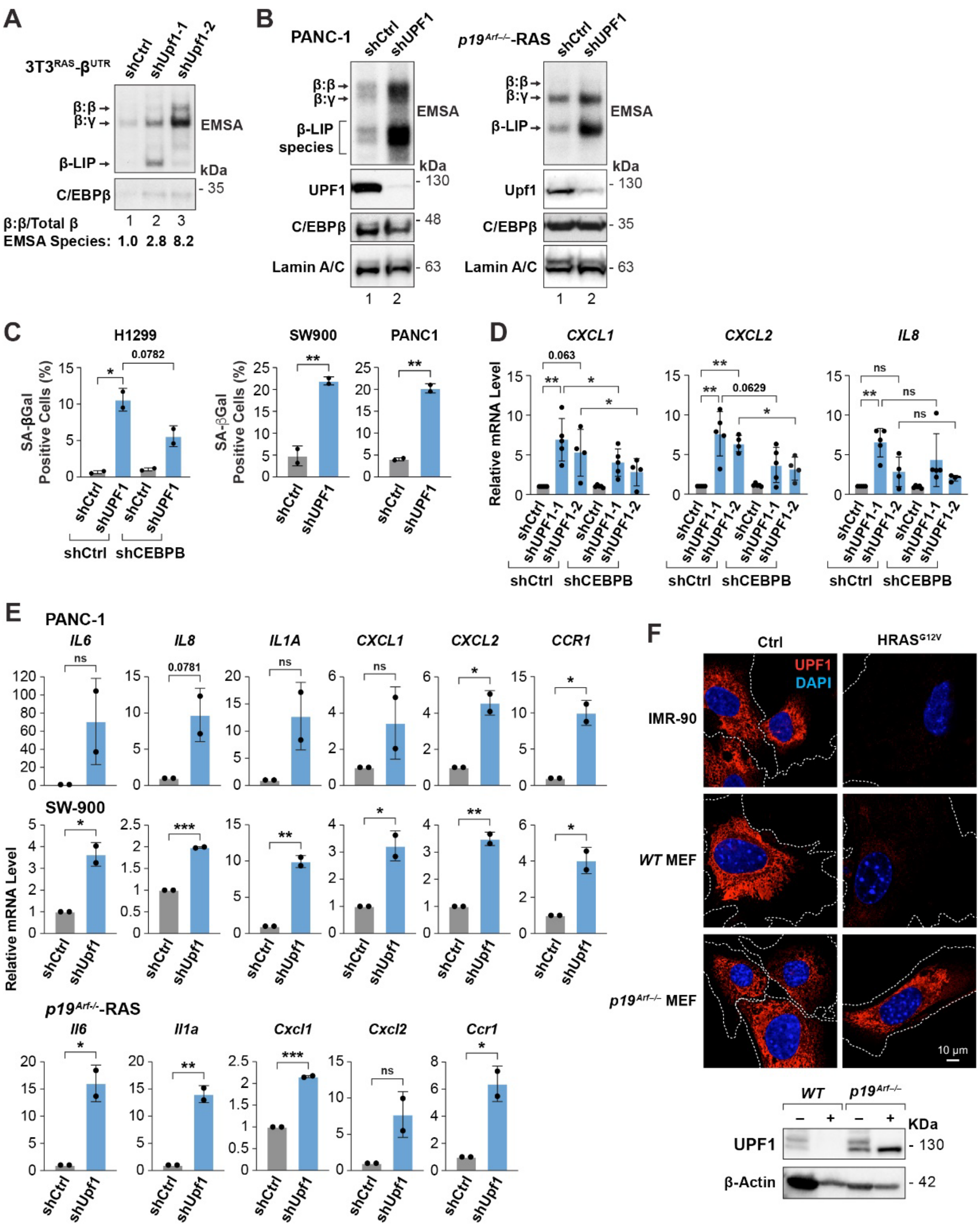
A
3T3RAS-βUTR
shCtrl
shUpf1-1
shUpf1-2
β:β
β:γ
β-LIP
EMSA
kDa
C/EBPβ
35
β:β/Total β
EMSA Species: 1.0 2.8 8.2
B
PANC-1
shCtrl
shUPF1
p19Arf–/–-RAS
β-LIP species
UPF1
Upf1
Lamin A/C
C
H1299
SW900
PANC1
SA-βGal Positive Cells (%)
shCEBPB
D
CXCL1
CXCL2
IL8
Relative mRNA Level
E
PANC-1
SW-900
IL6
IL8
IL1A
CCR1
Il6
Il1a
Cxcl1
Cxcl2
Ccr1
shUpf1
F
Ctrl
HRASG12V
UPF1
DAPI
IMR-90
WT MEF
p19Arf–/– MEF
10 µm
WT
KDa
β-Actin

Figure S3. UPF1 depletion activates the pro-senescence functions of C/EBPβ in tumor cells, Related to Figure 3.

(A) Upf1 silencing stimulates C/EBPβ DNA-binding activity in $3T3^{RAS}$-β^{UTR} cells. Nuclear extracts were analyzed by EMSA using a C/EBP binding site probe. The C/EBPβ homodimer EMSA band (β:β) was quantitated and normalized to total C/EBPβ binding (all species). **(B)** Depletion of UPF1 stimulates C/EBPβ DNA-binding activity in PANC-1 cells and $HRAS^{G12V}$-transformed *p19*$^{Arf-/-}$ MEFs. **(C)** Analysis of senescence (SA-βGal staining) in a panel of human tumor cell lines depleted for UPF1. **(D)** Activation of SASP genes in UPF1-depleted A549 cells, without and with *CEBPB* knockdown. The indicated genes were analyzed by RT-qPCR. **(E)** Effect of UPF1 silencing on SASP gene expression in a subset of the cell lines shown in **C** and in $HRAS^{G12V}$-transformed *p19*$^{Arf-/-}$ MEFs. The indicated genes were analyzed by RT-qPCR. **(F)** RAS-induced senescence is associated with UPF1 down-regulation. IF images show UPF1 expression and localization in human IMR-90 fibroblasts and primary *WT* and *p19*$^{Arf-/-}$ MEFs, without or with $HRAS^{G12V}$. Bottom: immunoblot confirming changes in UPF1 levels in *WT* and *p19*$^{Arf-/-}$ MEFs, without or with $HRAS^{G12V}$.

Scale bar dimensions are shown in the micrographs.

Statistical significance was determined using Student's t test. *$p < 0.05$,**$p < 0.01$,***$p < 0.001$; ns, not significant.

Figure S4

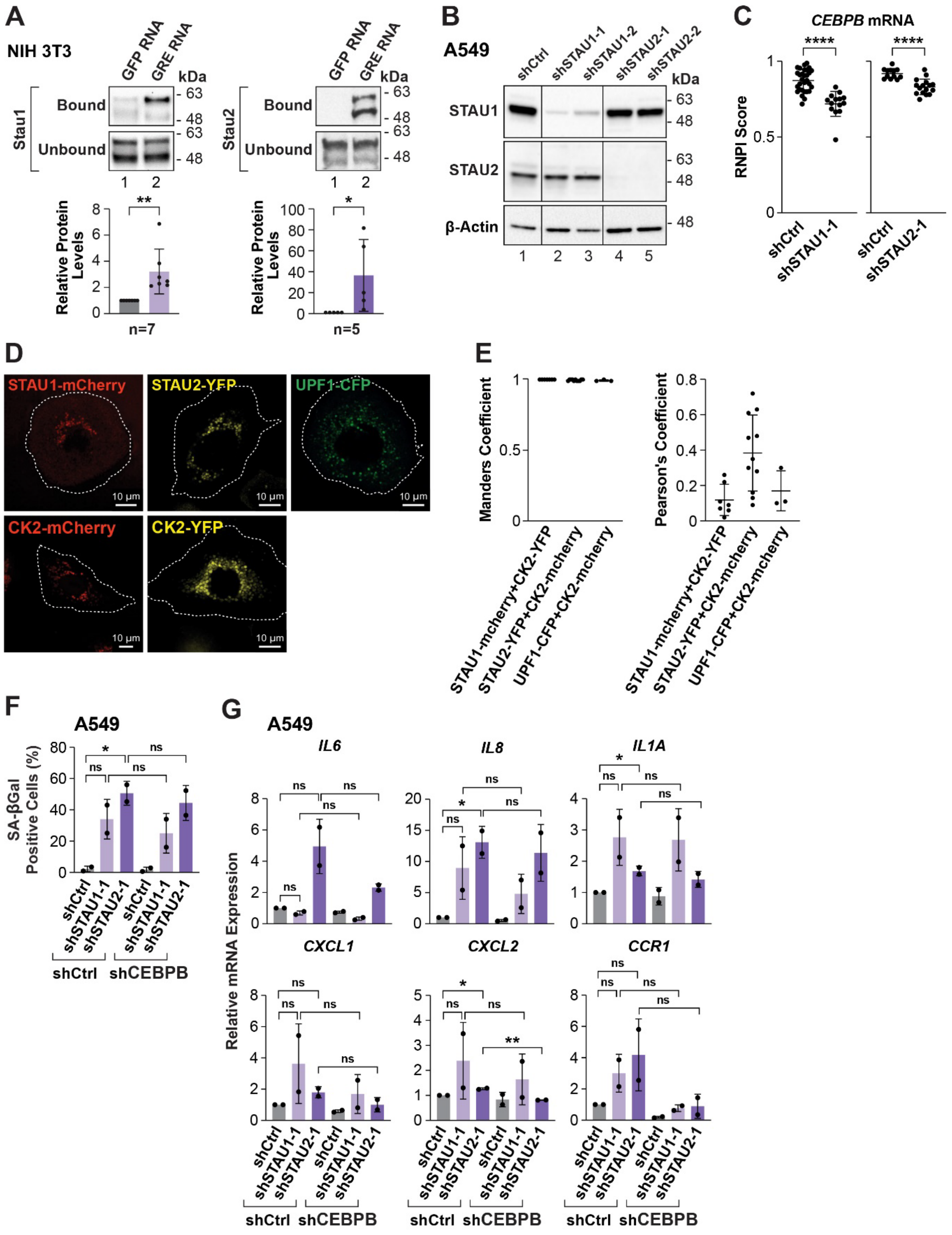

A
NIH 3T3
GFP RNA
GRE RNA
kDa
Stau1
Bound
Unbound
Stau2
Relative Protein Levels
n=7
n=5
B
A549
shCtrl
shSTAU1-1
shSTAU1-2
shSTAU2-1
shSTAU2-2
STAU1
STAU2
β-Actin
C
CEBPB mRNA
RNPI Score
shCtrl
shSTAU1-1
shSTAU2-1
D
STAU1-mCherry
STAU2-YFP
UPF1-CFP
CK2-mCherry
CK2-YFP
10 µm
E
Manders Coefficient
Pearson's Coefficient
STAU1-mcherry+CK2-YFP
STAU2-YFP+CK2-mcherry
UPF1-CFP+CK2-mcherry
F
A549
SA-βGal Positive Cells (%)
shCtrl
shCEBPB
G
A549
IL6
IL8
IL1A
CXCL1
CXCL2
CCR1
Relative mRNA Expression

Figure S4. STAU depletion in tumor cells induces senescence and weakly activates SASP genes, Related to Figure 4.

(A) Stau1 and Stau2 are enriched in the GRE-bound fraction. Samples described in Fig. 1E were analyzed by immunoblotting for Stau1 or Stau2. **(B)** Knockdown efficiency and specificity of STAU1 and STAU2 shRNAs in A549 cells as assayed by immunoblotting. **(C)** Relative nuclear proximity index (RNPI) scores for *CEBPB* mRNA FISH signals in A549 cells, without and with STAU1/2 depletion (analysis of the experiment in Fig. 4D). **(D)** Images of fluorescently tagged STAU1, STAU2, UPF1 and CK2α expressed individually in A549 cells. STAU1, STAU2 and UPF1 display perinuclear localization, demonstrating their intrinsic perinuclear targeting independent of co-expressed CK2α. **(E)** Manders' overlap and Pearson's correlation coefficients for fluorescently tagged STAU1, STAU2 and UPF1 co-expressed with CK2α (analysis of experiment in Fig. 4F). **(F)** Senescent cells (SA-βGal staining) in control or *CEBPB*-depleted A549 cells without and with STAU1/2 depletion ($n \geq 400$ cells). **(G)** STAU1 or STAU2 depletion in A549 cells modestly upregulates SASP genes in a manner that is partially dependent on C/EBPβ. Scale bar dimensions are shown in the micrographs.

Statistical significance was determined using Student's t test. $^{*}p < 0.05$, $^{**}p < 0.01$, $^{****}p < 0.0001$; ns, not significant.

A Homology Between Mouse and Human *CEBPB* 3'UTRs

```
mCebpb CGCGGC----GCGGGTGGCGTGGGGGGCGCCGCGGCC---ACCCTGGGCACCGT------ 47
hCEBPB CGCGGCCCCCGCGCGCGTCCCCCTGCCGGCCGGGGCTGAGACTCCGGGGAGCGCCCGCGC 60
       ******    *** * * *      *    **** ***     ** * *** * **

mCebpb --GCGCCCT-GCCCC-----------GCGC-------GCTCCGGCCCCGCG--------- 77
hCEBPB CCGCGCCCTCGCCCCCGCCCCCGGCGGCGCCGGCAAAACTTTGGCACTGGGGCACTTGGC 120
         ******* *****           ****         **  *** * * *

mCebpb --CGCG----------------------------------------------------CG 83
hCEBPB AGCGCGGGGAGCCCGTCGGTAATTTTAATATTTTATTATATATATATATCTATATTTTTG 180
         ****                                                    *

mCebpb CCCGGACCA-CCGTGCGTGC----------CCTGCGCGC-------------------AC 113
hCEBPB TCCAAACCAACCGCACATGCAGATGGGGCTCCCGCCCGTGGTGTTATTTAAAGAAGAAAC 240
        **  **** ***  * ***          ** ** **                   **

mCebpb CTGCACCTGCACCGAGGG--GACA---CCGCGGGCACACCGCGGGCACCGCGCGCACGCA 168
hCEBPB GTCTATGTGTACAGATGAATGATAAACTCTCTGCTTCTCCCTCTGCCCCTCTC-CAGGCG 299
        *  *  ** ** ** *   ** *    * * *   * **    ** ** * * ** **

mCebpb CCTGCACAGCGCACCGGGTTTCGGGACTTGATGCAATCCGGATCAAACGTGGCTGAGCGC 228
hCEBPB CCGGCG-GGCGGGCCGG-TTTCGA-AGTTGATGCAATC-GGTTTAAACATGGCTGAACGC 355
       ** **   ***  **** *****  * *********** ** * **** ******* ***

mCebpb GTGTGGACACGGGACTGACGCAACACACGTGTAACTGTCAGCCGGGCCCTGAGTAATCAC 288
hCEBPB GTGTGTACACGGGACTGACGCAACCCACGTGTAACTGTCAGCCGGGCCCTGAGTAATCGC 415
       ***** ******************* ********************************* *

mCebpb TTAAAGATGTTCCTGCGGGGTTGTTGATGTTTTTGGTTTTGTTTTTGTTTTTTGTTTTGT 348
hCEBPB TTAAAGATGTTCCTACGGGCTTGTTGCTGTT---GAT-----------------GTTTTGT 456
       ************** **** ****** ****   * *                *******

mCebpb TTTGTTTTTTTTTTTTGGTCTTATTATTTTTTTTTGTATTATATAAAAAAGTTCTATTTCTA 408
hCEBPB TTTGTTTTGTTTTTTGGTCTT-------TTTTTGTATTATA-AAAAATAATCTATTTCTA 508
       ******** ************       ************** *****   **********

mCebpb TGAGAAAAGAGGCGTATGTATATTTTGAGAACCTTTTCCGTTTCGAGCATTAAAGTGAAG 468
hCEBPB TGAGAAAAGAGGCGTCTGTATATTTTGGGAATCTTTTCCGTTTCAAGCATTAA---GAAC 565
       *************** *********** *** ************ ********   ***

mCebpb ACATTTTAATAAACTTTTTTG-GAGAATGTTTA-AAAGCC--------------- 506
hCEBPB AC-TTTTAATAAACTTTTTTTGAGAATGGTTACAAAGCCTTTTGGGGGCAGTAA 619
     ** ****************** ******* *** ******
```

B

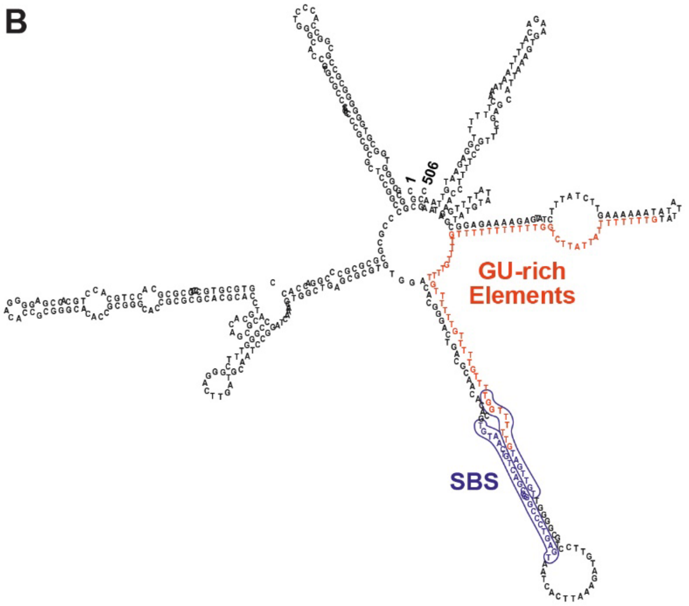

C Alignment of Mouse and Human *CEBPB* 3'UTRs with Intramolecular *ARF1* SBS

```
mCebpb   GACTGACGCAACACACGTGTAACTGTCAGCCGGGCCCTGAGTAATCACTTAAAGATGT   298
hCEBPB   GACTGACGCAACCCACGTGTAACTGTCAGCCGGGCCCTGAGTAATCGCTTAAAGATGT   425
hARF1                     TGTGAGTGCCAGAAGCTGCCTCCGT
                          *** * ** ***  *   ***  **
                                 ½ SBS(1)

mCebpb   TCCTGCGGGGTTGTTGATGTTTTTGGTTTTGTTTTTGTTTTTTGTTTTGTTTTGTTTT   356
hCEBPB   TCCTACGGGCTTGTTGCTGTT---GAT----------------GTTTTGTTTTGTTTT   464
hARF1           AATGAGGCAGTTTCTGGTACTCCTA
                 **  *  ***   * *
                    ½ SBS(2)

mCebpb   TTTTTTTGGTCTTATTATTTTTTTTTGTATTATATAAAAAAGTTCTATTTCTATGAGAA   414
hCEBPB   GTTTTTTGGTCTT-------TTTTTGTATTATA-AAAAATAATCTATTTCTATGAGAA   514
```

D

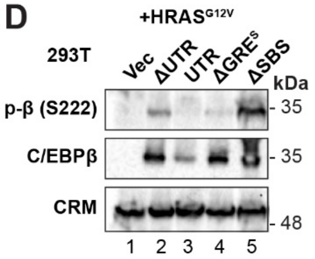

E

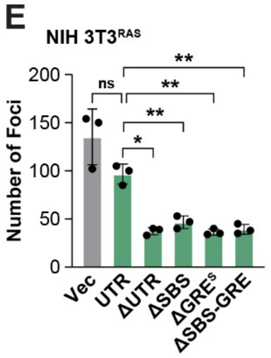

Figure S5. Conservation of the SBS/GRE region in mouse and human *CEBPB* 3′UTRs and analysis of SBS/GRE mutants, Related to Figure 5.

(A) Alignment of mouse and human *CEBPB* 3′UTRs. The strongest conservation occurs in the 3' regions surrounding the GU-rich sequence (orange). **(B)** An RNA secondary structure prediction model, RNAfold[4], shows a stem-loop formed by part of the GU-rich sequence (orange) paired with a 5' adjacent sequence containing a predicted STAU binding site (SBS) (purple). **(C)** A putative SBS adjacent to the GRE region is identified by sequence homology to an intramolecular SBS identified in the *ARF1* 3′UTR[5]. Diagram shows conservation of the potential SBS element (purple, boxed) in mouse and human *CEBPB*, its sequence similarity to the *ARF1* SBS and its position relative to the GRE (orange). Note the partial overlap between the GRE and SBS elements. **(D)** $HRAS^{G12V}$-induced phosphorylation on C/EBPβ Ser222 (CK2 site) in 293T cells, comparing several 3′UTR deletion mutants. **(E)** Focus formation assays using NIH $3T3^{RAS}$ cells expressing *Cebpb* 3′UTR mutant constructs. $n = 3$ independent replicates.

Statistical significance was determined using Student's paired t test. *$p < 0.05$, **$p < 0.01$; ns, not significant.

Figure S6

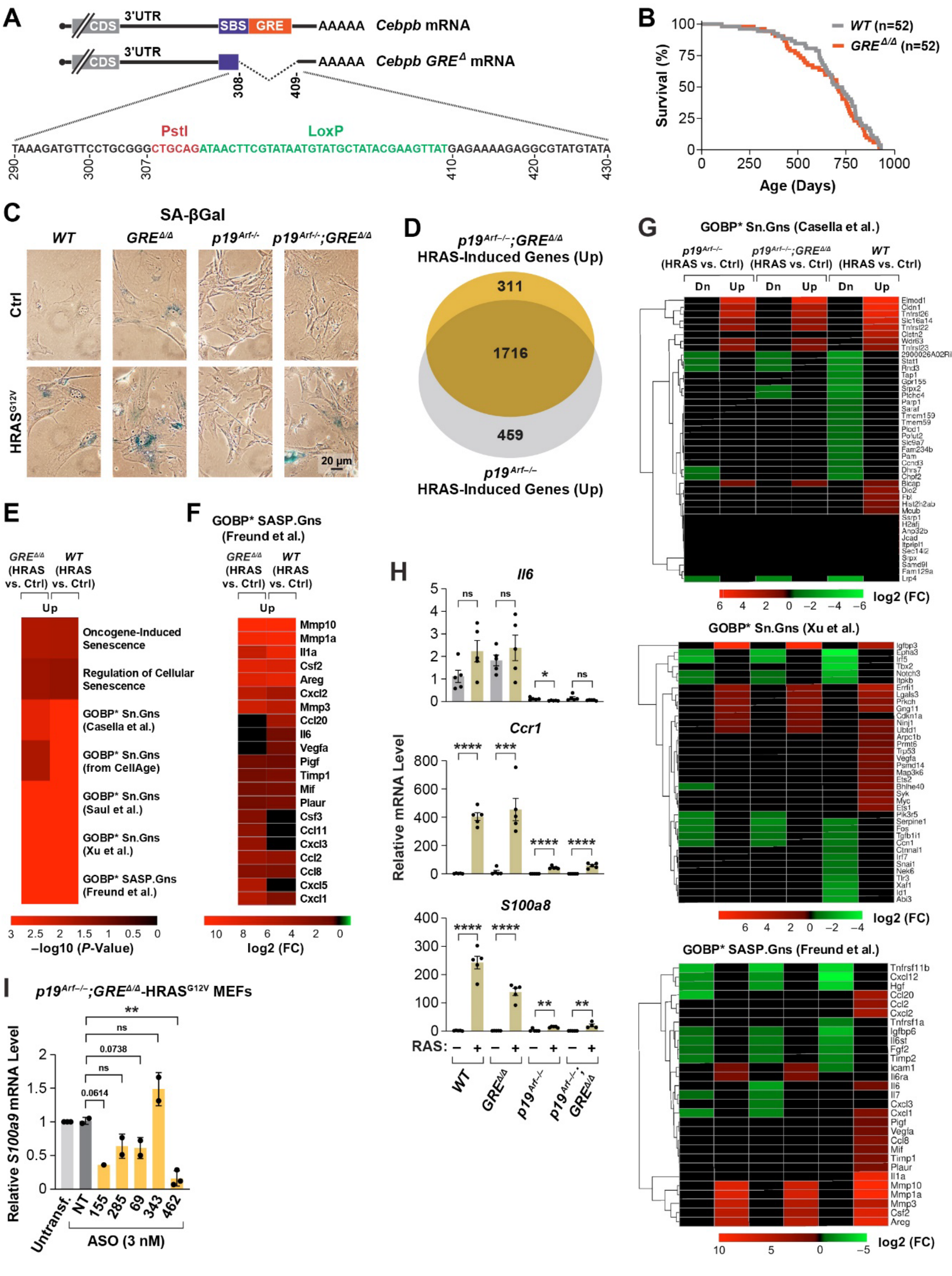
A
CDS
3'UTR
SBS
GRE
AAAAA
Cebpb mRNA
Cebpb GRE^Δ mRNA
308-
409-
PstI
LoxP
TAAAGATGTTCCTGCGGGCTGCAGATAACTTCGTATAATGTATGCTATACGAAGTTATGAGAAAAGAGGCGTATGTATA
290-
300-
307-
410-
420-
430-
B
WT (n=52)
GRE^Δ/Δ (n=52)
Survival (%)
Age (Days)
C
SA-βGal
WT
GRE^Δ/Δ
p19^Arf-/-
p19^Arf-/-;GRE^Δ/Δ
Ctrl
HRAS^G12V
20 µm
D
p19^Arf–/–;GRE^Δ/Δ
HRAS-Induced Genes (Up)
311
1716
459
p19^Arf–/–
HRAS-Induced Genes (Up)
E
GRE^Δ/Δ (HRAS vs. Ctrl)
WT (HRAS vs. Ctrl)
Up
Oncogene-Induced Senescence
Regulation of Cellular Senescence
GOBP* Sn.Gns (Casella et al.)
GOBP* Sn.Gns (from CellAge)
GOBP* Sn.Gns (Saul et al.)
GOBP* Sn.Gns (Xu et al.)
GOBP* SASP.Gns (Freund et al.)
–log10 (P-Value)
F
GOBP* SASP.Gns (Freund et al.)
Mmp10
Mmp1a
Il1a
Csf2
Areg
Cxcl2
Mmp3
Ccl20
Il6
Vegfa
Pigf
Timp1
Mif
Plaur
Csf3
Ccl11
Cxcl3
Ccl2
Ccl8
Cxcl5
Cxcl1
log2 (FC)
G
GOBP* Sn.Gns (Casella et al.)
p19^Arf–/– (HRAS vs. Ctrl)
p19^Arf–/–;GRE^Δ/Δ (HRAS vs. Ctrl)
WT (HRAS vs. Ctrl)
Dn
Up
log2 (FC)
GOBP* Sn.Gns (Xu et al.)
GOBP* SASP.Gns (Freund et al.)
H
Il6
Ccr1
S100a8
Relative mRNA Level
ns
*
****
***
**
RAS:
WT
GRE^Δ/Δ
p19^Arf–/–
p19^Arf–/–; GRE^Δ/Δ
I
p19^Arf–/–;GRE^Δ/Δ-HRAS^G12V MEFs
Relative S100a9 mRNA Level
0.0614
0.0738
ns
**
Untransf.
NT
155
285
69
343
462
ASO (3 nM)

Figure S6. RAS induces senescence in *p19^Arf-/-^;GRE^Δ/Δ^* MEFs, Related to Figure 6.

(A) Diagram of the mouse *Cebpb* 3′UTR and GRE^{Δ} allele. GRE^{Δ} was generated by Cre-mediated recombination of a floxxed allele containing LoxP sites flanking the GRE motif. **(B)** Kaplan-Meier survival curves of *WT* and $GRE^{\Delta/\Delta}$ mice, showing no significant difference in life spans (Log-rank test; p = 0.35). *n*, number of animals. **(C)** Representative SA-βGal staining images of *WT*, $GRE^{\Delta/\Delta}$, $p19^{Arf-/-}$, and $p19^{Arf-/-};GRE^{\Delta/\Delta}$ MEFs, ± $HRAS^{G12V}$. Quantification of SA-βGal positive cells in several independently-derived MEF isolates is shown in Fig. 6C. **(D)** Venn diagram comparing $HRAS^{G12V}$-induced (up-regulated) genes in $p19^{Arf-/-}$ and $p19^{Arf-/-};GRE^{\Delta/\Delta}$ MEFs. The plot is based on RNA-seq data from five MEF isolates of each genotype, ± $HRAS^{G12V}$. **(E,F)** Comparison of $HRAS^{G12V}$-induced senescence genes **(E)** and SASP genes **(F)** in *WT* and $GRE^{\Delta/\Delta}$ MEFs. See Figs. 6E and S6G for details. **(G)** Gene-level heat maps of senescence-associated or SASP genes that are significantly up- or down-regulated by $HRAS^{G12V}$ in MEFs of the indicated genotypes. DEGs were subjected to GOBP analysis using customized gene sets representing the indicated reference gene signatures for senescence[6,7] or the SASP[8]. These genes collectively showed enrichment in the corresponding published senescence signatures (see Fig. 6E). The enriched genes for each contrast were retrieved to create gene-level heatmaps showing fold changes in log2 scale. Red, up-regulation; green, down-regulation. **(H)** Analysis of SASP genes induced by $HRAS^{G12V}$ in *WT* and mutant MEFs. See legend to Fig. 6F for details. **(I)** Knockdown efficiency of five candidate *S100a9* ASOs tested at 3 nM in $p19^{Arf-/-};GRE^{\Delta/\Delta}$-$HRAS^{G12V}$ MEFs. *S100a9* mRNA levels were normalized to those in untransfected cells. NT, universal non-targeting ASO.

Statistical significance was determined using unpaired **(F)** or paired **(G)** Student's t tests, or Log-rank test **(B)**. *p < 0.05, **p < 0.01, ***p < 0.001, ****p < 0.0001; ns, not significant.

Figure S7

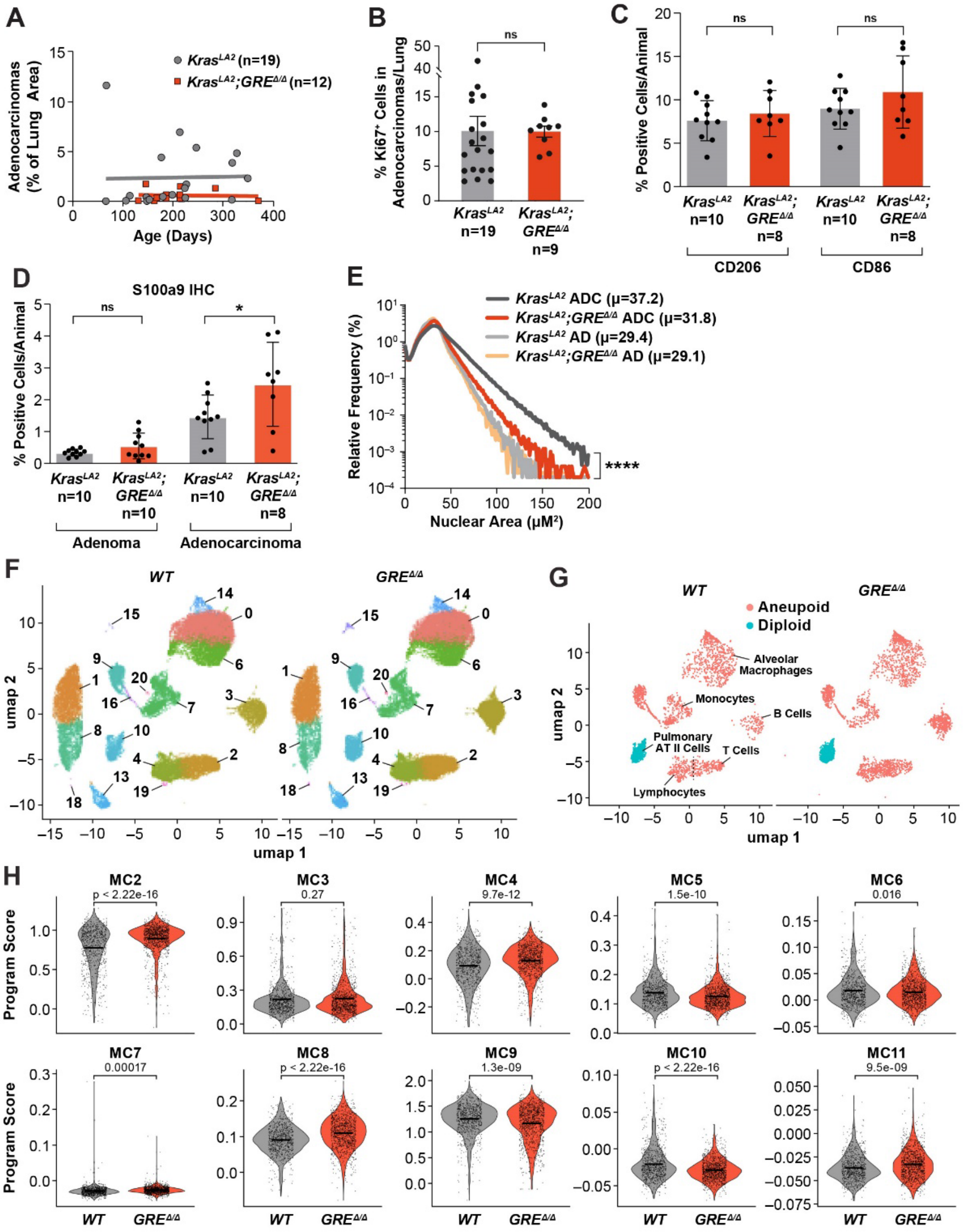

A
Adenocarcinomas (% of Lung Area)
Kras^LA2 (n=19)
Kras^LA2;GRE^Δ/Δ (n=12)
Age (Days)
B
% Ki67+ Cells in Adenocarcinomas/Lung
ns
Kras^LA2 n=19
Kras^LA2; GRE^Δ/Δ n=9
C
% Positive Cells/Animal
ns
Kras^LA2 n=10
Kras^LA2; GRE^Δ/Δ n=8
CD206
CD86
D
S100a9 IHC
% Positive Cells/Animal
ns
*
Kras^LA2 n=10
Kras^LA2; GRE^Δ/Δ n=10
Kras^LA2 n=10
Kras^LA2; GRE^Δ/Δ n=8
Adenoma
Adenocarcinoma
E
Kras^LA2 ADC (μ=37.2)
Kras^LA2;GRE^Δ/Δ ADC (μ=31.8)
Kras^LA2 AD (μ=29.4)
Kras^LA2;GRE^Δ/Δ AD (μ=29.1)
Relative Frequency (%)
Nuclear Area (μM²)
****
F
WT
GRE^Δ/Δ
umap 1
umap 2
G
WT
GRE^Δ/Δ
Aneupoid
Diploid
Alveolar Macrophages
Monocytes
B Cells
Pulmonary AT II Cells
T Cells
Lymphocytes
umap 1
umap 2
H
Program Score
MC2 p < 2.22e-16
MC3 0.27
MC4 9.7e-12
MC5 1.5e-10
MC6 0.016
MC7 0.00017
MC8 p < 2.22e-16
MC9 1.3e-09
MC10 p < 2.22e-16
MC11 9.5e-09
WT
GRE^Δ/Δ

Figure S7

I

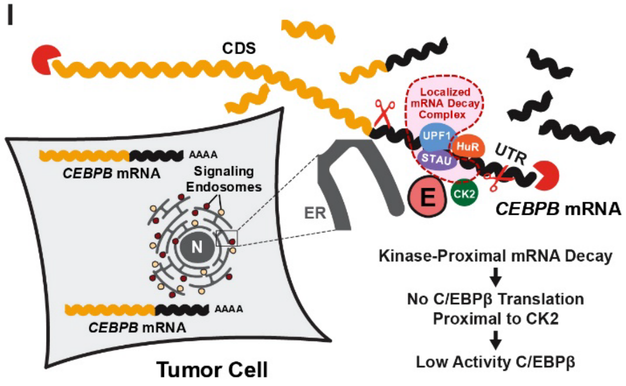

Figure S7. Analysis of tumors in *Kras*$^{LA2/+}$*;Cebpb-GRE*$^{\Delta/\Delta}$ mice and summary model for kinase-proximal mRNA decay, Related to Figure 7.

(A) Lack of correlation between age at clinical sacrifice and adenocarcinoma burdens in *Kras*$^{LA2/+}$and *Kras*$^{LA2/+}$*;GRE*$^{\Delta/\Delta}$ mice. The adenocarcinoma data of Fig. 7C is presented as a scatter plot versus age. **(B)** Percent Ki67 positive cells within adenocarcinoma lesions for each genotype. **(C)** Macrophage infiltration in *Kras*$^{LA2/+}$and *Kras*$^{LA2/+}$*;GRE* $^{\Delta/\Delta}$ tumors. Lung sections were stained for CD206 (M2-type) or CD86 (M1-type) macrophages; data are shown as % positive cells in tumor areas per animal. **(D)** S100a9 positive cells in *Kras*^{LA2} and *Kras* ^{LA2}*;GRE* $^{\Delta/\Delta}$ tumors. Lung sections were analyzed by IHC staining for S100a9. Data are expressed as % positive cells in tumor areas per animal, separated by tumor grade. **(E)** Frequency distribution ($\log_{10}$ scale) of nuclear size (area) in adenomas (AD) and adenocarcinomas (ADC) of both genotypes. Population means (μ) are shown. Number of nuclei analyzed by HALO: *Kras*^{LA2} ADC (n = 1,834,153); *Kras*^{LA2}*;GRE*$^{\Delta/\Delta}$ ADC (n = 498,806); *Kras*^{LA2} AD (n = 494,181); *Kras* ^{LA2}*;GRE*$^{\Delta/\Delta}$ AD (n = 380,307). **(F)** Primary clustering of scRNA-seq data from lung tumor samples, separated by genotype. **(G)** Aneuploidy analysis of the epithelial subset and major immune cell types. Aneuploid cells were identified using CopyKAT. **(H)** Program scores for single-cell transcriptomes of each genotype compared to Marjanovic clusters (MC) 2-11. See Fig. 7H for details. **(I)** Model depicting 3′UTR-directed, perinuclear decay of *CEBPB* transcripts in tumor cells. *CEBPB* mRNA decay occurs on perinuclear CK2α-containing endosomes, where STAU proteins also co-localize. These signaling endosomes are embedded within the perinuclear ER network.[3] Local mRNA decay also requires

UPF1—which may be recruited to endosomes through STAU proteins[5] and other RNA-binding proteins—as well as SMG6 and 7 (not shown). Kinase-proximal *CEBPB* mRNA decay at signaling endosomes prevents CK2-mediated phosphorylation of C/EBPβ, maintaining it in a low-activity state that facilitates senescence bypass.

Supplemental references

1. Butter, F., Scheibe, M., Morl, M., and Mann, M. (2009). Unbiased RNA-protein interaction screen by quantitative proteomics. Proc. Natl. Acad. Sci. USA *106*, 10626–10631. 10.1073/pnas.0812099106.
2. Flury, V., Restuccia, U., Bachi, A., and Muhlemann, O. (2014). Characterization of phosphorylation- and RNA-dependent UPF1 interactors by quantitative proteomics. Journal of proteome research *13*, 3038–3053. 10.1021/pr5002143.
3. Basu, S., Luke, B.T., Karim, B., Martin, N., Lockett, S., Das, S., Andresson, T., Saylor, K., Kozlov, S., Bassel, L., et al. (2022). CK2 signaling from TOLLIP-dependent perinuclear endosomes is an essential feature of KRAS mutant cancers. bioRxiv, 2022.2004.2005.487175. 10.1101/2022.04.05.487175.
4. Gruber, A.R., Lorenz, R., Bernhart, S.H., Neubock, R., and Hofacker, I.L. (2008). The Vienna RNA websuite. Nucleic Acids Res. *36*, W70–74. 10.1093/nar/gkn188.
5. Kim, Y.K., Furic, L., Desgroseillers, L., and Maquat, L.E. (2005). Mammalian Staufen1 recruits Upf1 to specific mRNA 3'UTRs so as to elicit mRNA decay. Cell *120*, 195–208. 10.1016/j.cell.2004.11.050.
6. Casella, G., Munk, R., Kim, K.M., Piao, Y., De, S., Abdelmohsen, K., and Gorospe, M. (2019). Transcriptome signature of cellular senescence. Nucleic Acids Res. *47*, 11476. 10.1093/nar/gkz879.
7. Xu, P., Wang, M., Song, W.M., Wang, Q., Yuan, G.C., Sudmant, P.H., Zare, H., Tu, Z., Orr, M.E., and Zhang, B. (2022). The landscape of human tissue and cell type specific expression and co-regulation of senescence genes. Mol Neurodegener *17*, 5. 10.1186/s13024-021-00507-7.
8. Freund, A., Orjalo, A.V., Desprez, P.Y., and Campisi, J. (2010). Inflammatory networks during cellular senescence: causes and consequences. Trends Mol Med *16*, 238–246. 10.1016/j.molmed.2010.03.003.

Western Blots from Main Figures

Fig 1B

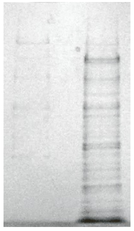

Fig 1E

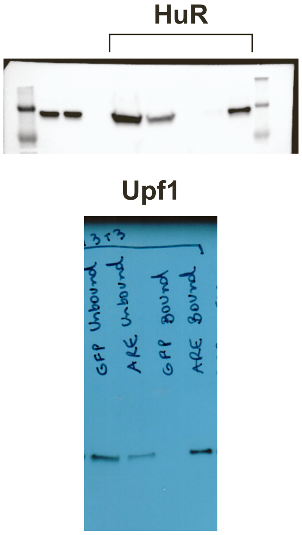

Fig 1F

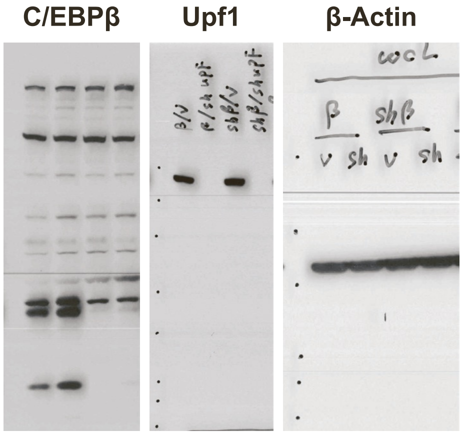

Fig 1H

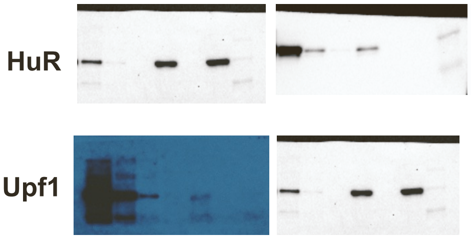

Fig 1G

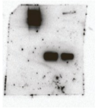

Fig 2C

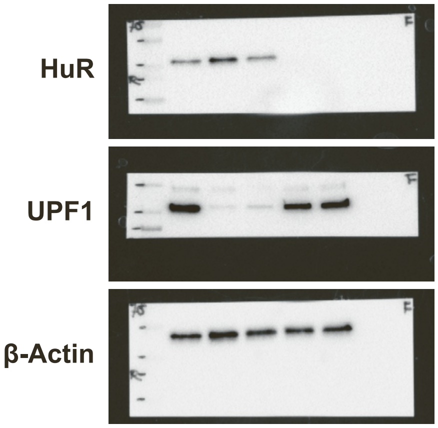

Fig 3A

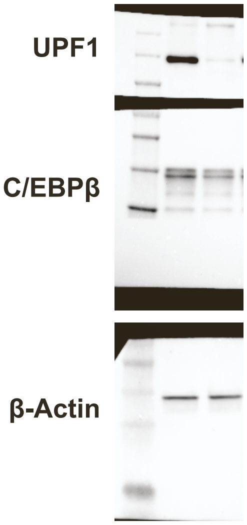

Fig 3C

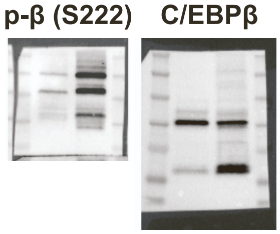

Fig 3D

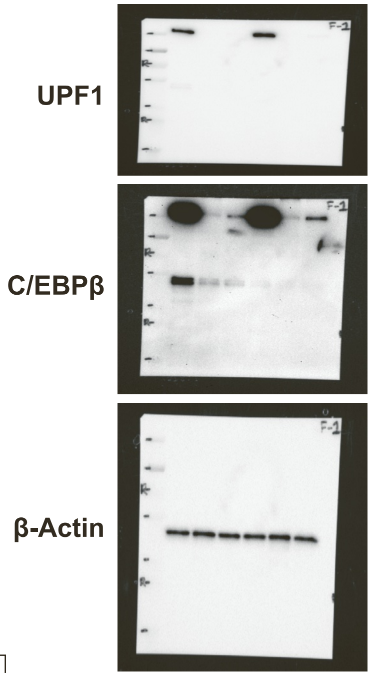

Fig 3H

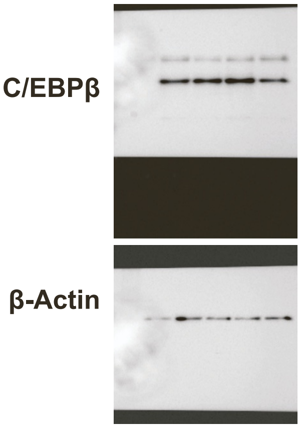

Fig 3I

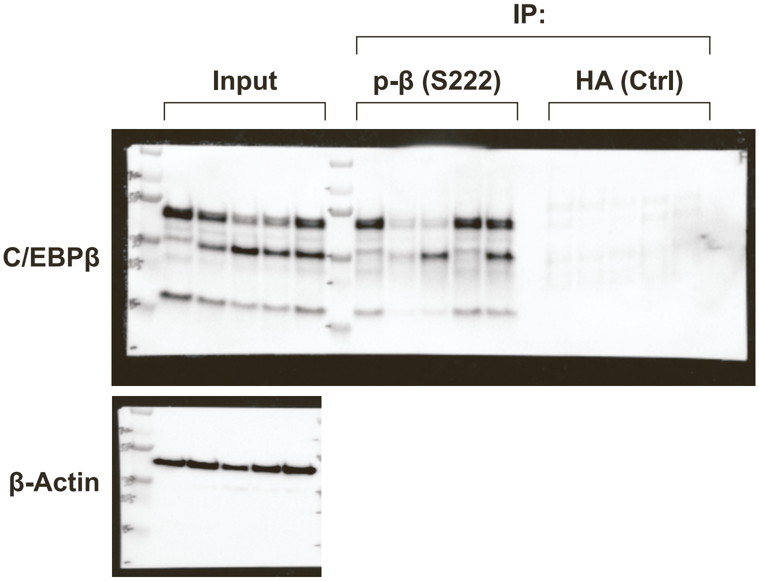

Western Blots from Main Figures

Fig 4A

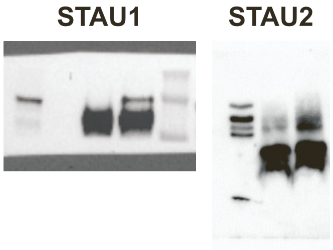

Fig 5B

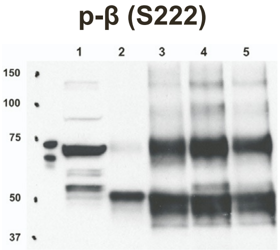

Fig 5D

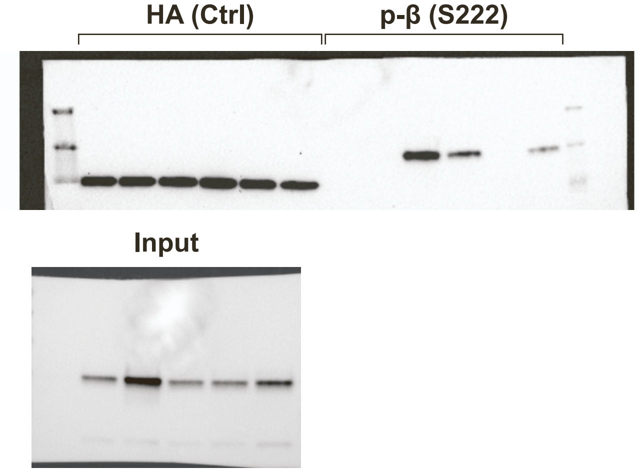

Fig 5F

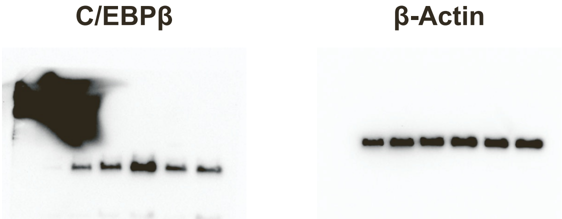

Fig 6A

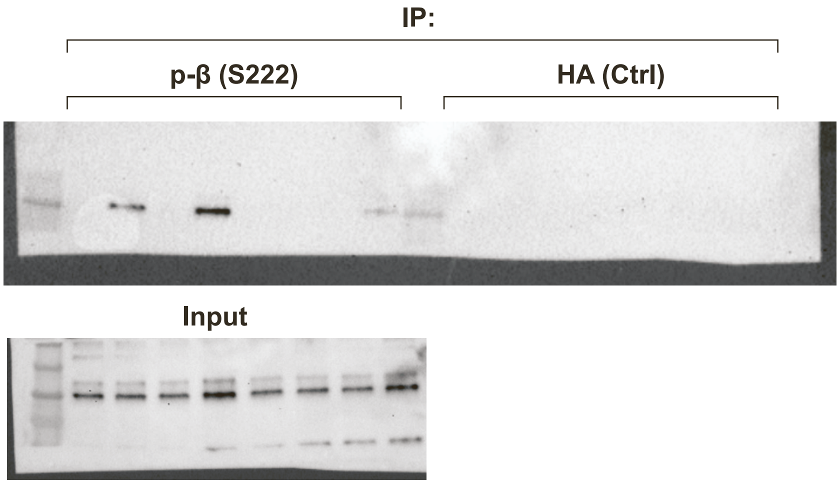

Fig 6C

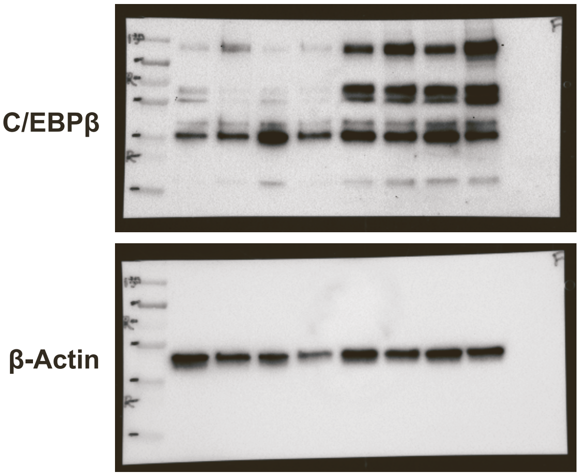

Fig 6I

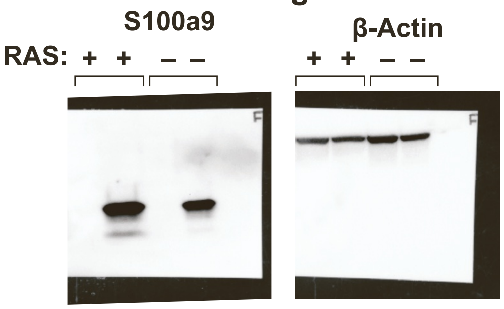

Western Blots from Supplementary Figures

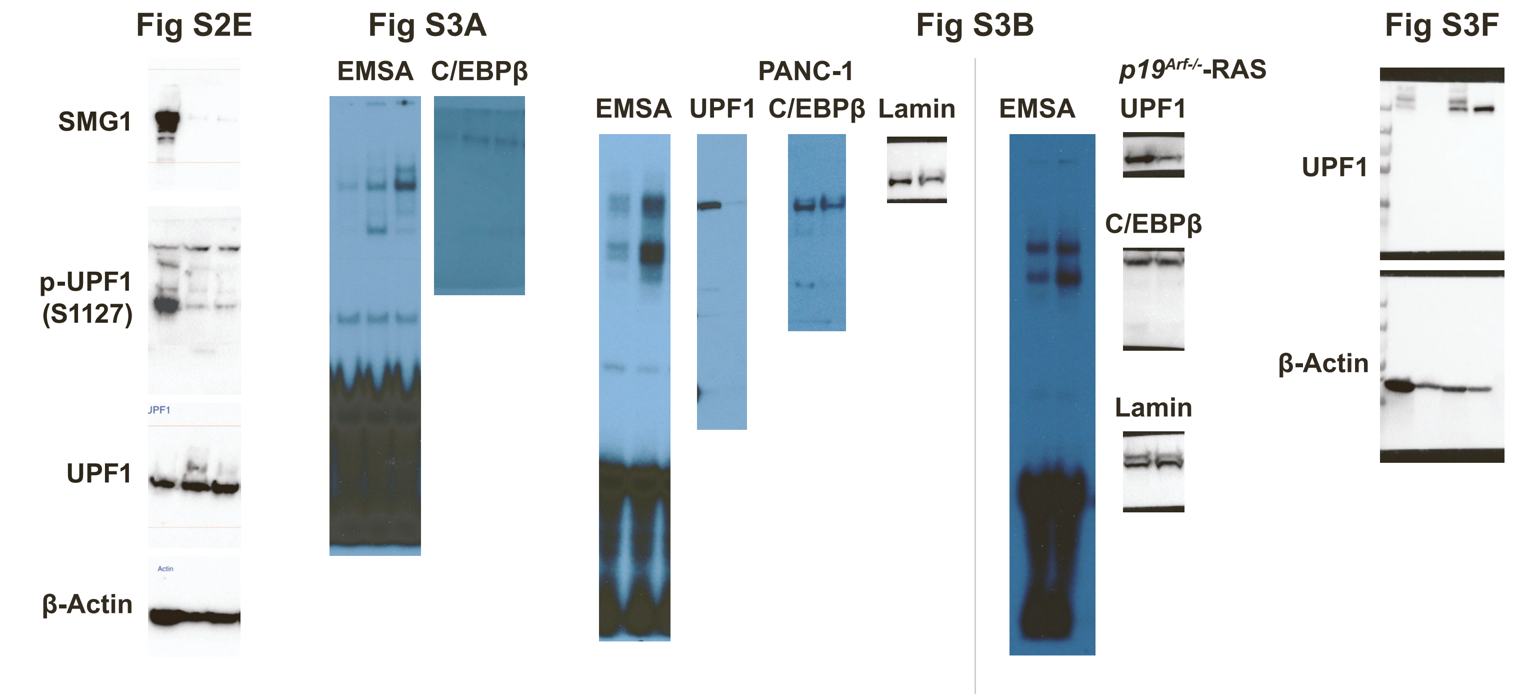

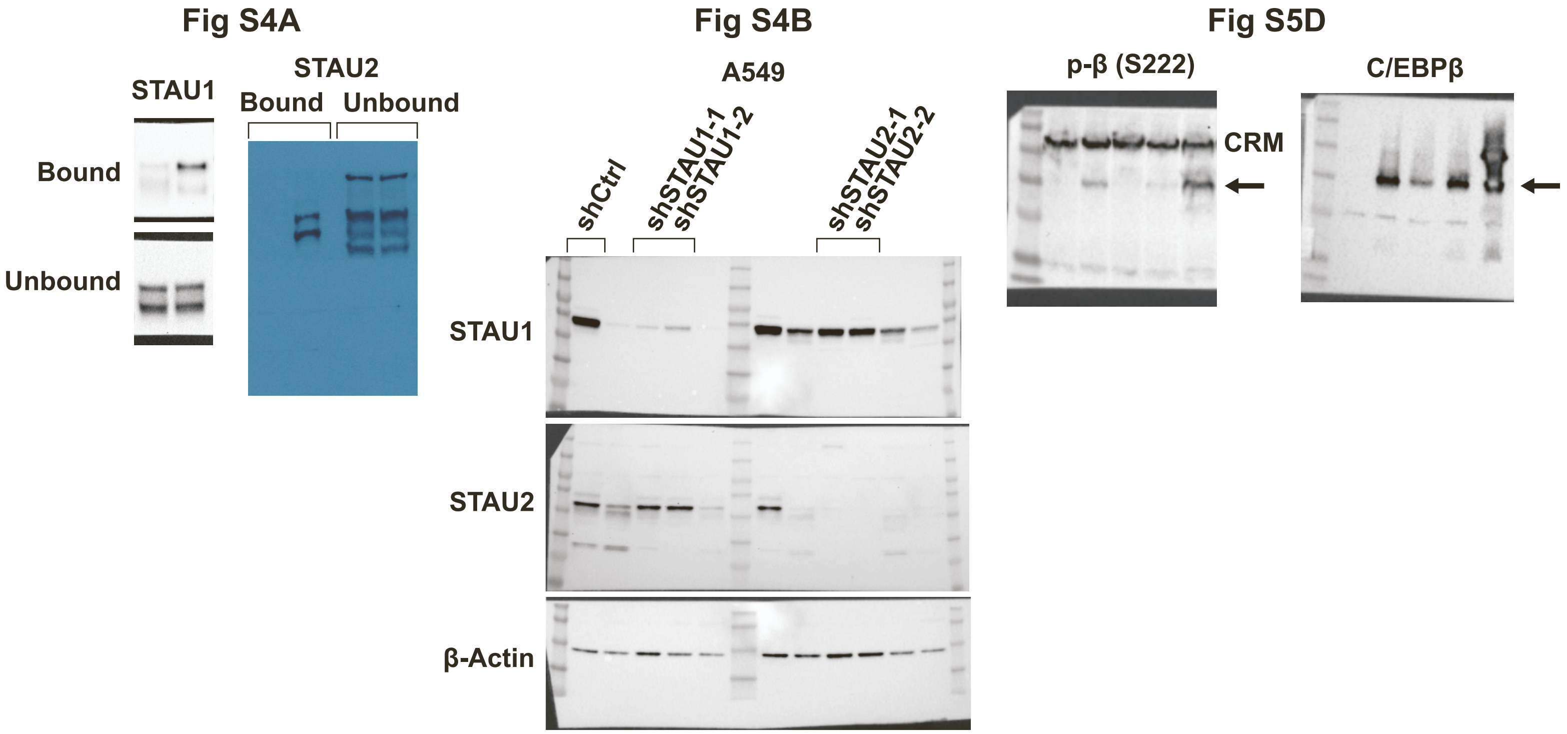

Supplement: Supplementary file 1 [file mmc1.pdf]
